# Detecting Genotype-Population Interaction Effects by Ancestry Principal Components

**DOI:** 10.3389/fgene.2020.00379

**Published:** 2020-04-21

**Authors:** Chenglong Yu, Guiyan Ni, Julius van der Werf, S. Hong Lee

**Affiliations:** ^1^Australian Centre for Precision Health, University of South Australia Cancer Research Institute, University of South Australia, Adelaide, SA, Australia; ^2^College of Medicine and Public Health, Flinders University, Bedford Park, SA, Australia; ^3^South Australian Health and Medical Research Institute, Adelaide, SA, Australia; ^4^Institute for Molecular Bioscience, The University of Queensland, Brisbane, QLD, Australia; ^5^School of Environmental and Rural Science, University of New England, Armidale, NSW, Australia

**Keywords:** genotype-phenotype relationship, complex traits, SNP-based heritability, genetic heterogeneity, UK Biobank, selection bias

## Abstract

Heterogeneity in the phenotypic mean and variance across populations is often observed for complex traits. One way to understand heterogeneous phenotypes lies in uncovering heterogeneity in genetic effects. Previous studies on genetic heterogeneity across populations were typically based on discrete groups in populations stratified by different countries or cohorts, which ignored the difference of population characteristics for the individuals within each group and resulted in loss of information. Here, we introduce a novel concept of genotype-by-population (G × P) interaction where population is defined by the first and second ancestry principal components (PCs), which are less likely to be confounded with country/cohort-specific factors. We applied a reaction norm model fitting each of 70 complex traits with significant SNP-heritability and the PCs as covariates to examine G × P interactions across diverse populations including white British and other white Europeans from the UK Biobank (*N* = 22,229). Our results demonstrated a significant population genetic heterogeneity for behavioral traits such as age at first sexual intercourse and academic qualification. Our approach may shed light on the latent genetic architecture of complex traits that underlies the modulation of genetic effects across different populations.

## Introduction

Most human traits are polygenic and their phenotypes are typically influenced by numerous genes and environmental factors, and possibly by their interactions, e.g., genotype-environment (G × E) interaction (Plomin et al., 1977; Mackay, 2001; Reddon et al., 2016). These traits have been termed as “complex traits,” which are distinguished from Mendelian traits that are shaped by a single or few major genes (Lander and Schork, 1994). Genome-wide association studies (GWAS) have successfully discovered thousands of associations between single-nucleotide polymorphisms (SNPs) and complex traits, which have revolutionized our understanding of the polygenic architecture of complex traits (Stranger et al., 2011; Goddard et al., 2016; Visscher et al., 2017). Subsequently, in order to increase the power and precision to identify more causal variants, there have been numerous follow-up studies using meta-analyses of GWAS summary statistics or mega-analyses of multiple GWAS by combining diverse data sources that usually span across different nations or populations (Torgerson et al., 2011; Nagel et al., 2018). However, many human complex traits [e.g., height and body mass index (BMI)] are substantially different between diverse populations (Guo et al., 2018). For instance, the mean height across European nations generally increases with latitude (Robinson et al., 2015). Although across-population differences in the mean values are often observed for the phenotypes of complex traits, the underlying genetic and environmental bases remain largely unknown (Robinson et al., 2015).

One way to understand such phenotypic heterogeneity lies in uncovering genetic differentiation for the traits captured by common variants across populations (Falconer and Mackay, 1996). Some studies (Maier et al., 2015; Robinson et al., 2015; Yang et al., 2015; Tropf et al., 2017) have focused on examining population genetic differentiation for several anthropometric, behavioral and psychiatric phenotypes, using whole-genome statistical methods such as applying bivariate genomic restricted maximum likelihood (GREML) (Lee et al., 2012) to estimate genetic correlations between populations from the United States and Europe for height and BMI (Yang et al., 2015) or determining interaction of genotype by seven sampling populations for behavioral traits by a GREML approach (Tropf et al., 2017). They reported significant evidence for interaction of genotype by populations in behavioral phenotypes (education and human reproductive behavior) and BMI (Tropf et al., 2017). The analytical method and designs used in their studies were based on discrete groups, which ignored the difference of population characteristics for the individuals within each group. Furthermore, the population groups used in their studies were classified according to their country of origin, thus the results were likely to reflect heterogeneity across countries due to country-specific factors (e.g., trait definition and measurement (van der Sluis et al., 2010; Evangelou et al., 2011; Manchia et al., 2013), cultural and societal difference and socio-economic status). In addition, genetic measurement errors (e.g., due to the genotyping platform or imputation quality) across different cohorts within country may further cause confounding with genuine genetic heterogeneity across populations (Tropf et al., 2017).

Principal component (PC) analysis provides a powerful tool to characterize populations and the first few PCs are typically used to control population stratifications in large-scale GWAS (Novembre and Stephens, 2008). PCs allow us to cluster individuals that are genetically similar to each other. Unlike discrete variables such as cohort and country, PCs are continuous variables that can differentiate individuals even within a cohort or a country according to their underlying genetic characteristics. Here, we introduce a novel concept of genotype-by-population (G × P) interaction where population is defined by the first and second PCs. It is of interest to test if different genotypes respond differently to the gradient of the first or second PC for complex traits using a whole-genome reaction norm model (RNM) (Ni et al., 2019), which has been recently introduced and allows fitting continuous environmental covariates, i.e., PCs in this study. RNM has been well established to estimate G × E interaction in agriculture (Gregorius and Namkoong, 1986; Jarquín et al., 2014) and ecology (Nussey et al., 2007). Furthermore, in this study we used the data source of UK BioBank (UKBB), which is a prospective cohort study with deep genetic and phenotypic data collected on approximately 500,000 individuals across the United Kingdom, aged between 40 and 69 at recruitment (Sudlow et al., 2015; Bycroft et al., 2018). Therefore, in our G × P interaction model applied to UKBB, the population characteristics for individuals are fully utilized and the findings are less likely to be confounded with country-specific factors or genetic measurement errors as mentioned above.

The aim of the study is to explore if there exists significant G × P interaction, which is also referred as genetic heterogeneity (heterogeneous genetic effects) across populations, for a wide range of complex traits. To do so, we applied the whole-genome RNM with PCs as continuous covariates to investigate G × P interactions for more than one hundred phenotypes using the UKBB data. The significant G × P interaction detected in this study may shed light on the latent genetic architecture of complex traits that underlies the modulation of genetic effects across different population backgrounds.

## Materials and Methods

### Data and Quality Control (QC)

**FIGURE 1:**
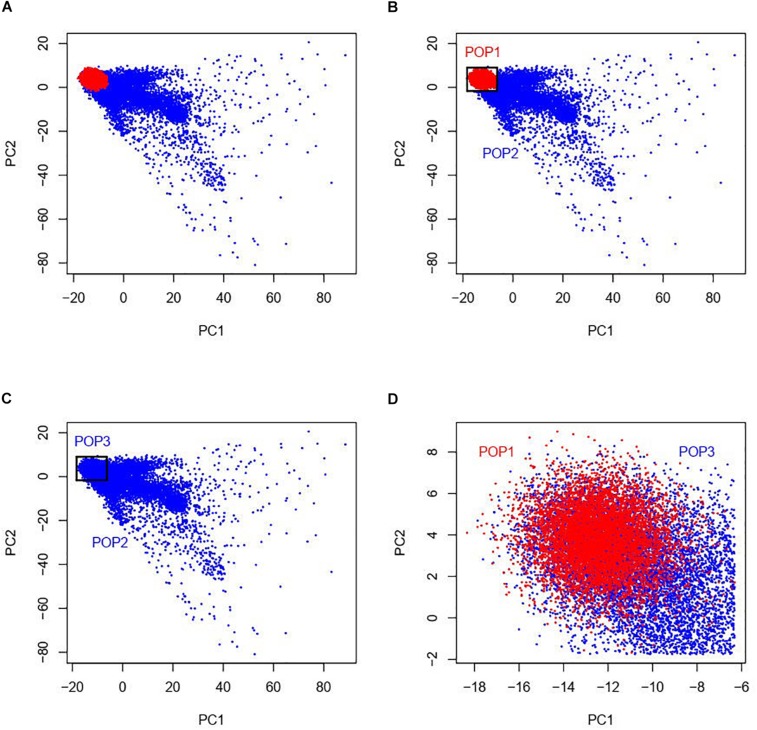
Two-dimensional scatter plots of PC1 and PC2 with red points representing white British individuals and blue points representing other white ethnic individuals from the UKBB. The white British group named as POP1 is situated within the group of the other white Europeans (see panel **A**). As shown in panels **(B,C)**, we used a geometric method by which we constructed a rectangle with maximums and minimums of PC1 and PC2 of POP1 as four sides and then group the individuals of the other white Europeans inside this rectangle, named as POP3. The rest of the other white Europeans except POP3 were named as POP2. As POP1 and POP3 are very close in terms of PCs, the individuals in the two data designs POP1 + POP2 and POP2 + POP3 have similar population structures while POP1 + POP3 was a negative control as there was little population difference among this combination (see panel **D**).

Our study was based on the UKBB data which contains approximately 500,000 individuals sampled across the United Kingdom (Bycroft et al., 2018). UKBB’s scientific protocol and operational procedures were reviewed and approved by the North West Multi-centre Research Ethics Committee (MREC), National Information Governance Board for Health & Social Care (NIGB), and Community Health Index Advisory Group (CHIAG). Research Ethics approval was obtained from University of South Australia Human Research Ethics Committee (HREC). According to the ethnic background (data field 21000), there are currently 472,242 individuals of the white British ancestry and 17,038 individuals of any other white ethnic background (not with British or Irish ethnicity) in the UKBB participants. In order to match the sample size between the white British and the other white ethnic individuals, we randomly selected 17,000 individuals from the white British group, totaling 34,038 admixed European populations considered in this study. As the information of the first and second ancestry PCs is efficient to infer genetic ancestry and geographical origin with a high accuracy in Europeans (Novembre et al., 2008), we examined a two-dimensional scatter plot of PC1 and PC2 provided by the UKBB of the 17,000 white British and the 17,038 other white ethnic subjects (Figure 1A). It is shown that the white British group is situated within the group of the other white Europeans and we named the white British group as POP1 (*N* = 17,000). As shown in Figures 1B,C, we used a geometric method by which we constructed a rectangle with maximums and minimums of PC1 and PC2 of the white British group as four sides and then group the individuals of the other white Europeans inside this rectangle, named as POP3 (*N* = 9,809). The rest of the other white Europeans except POP3 were named as POP2 (*N* = 7,229).

Our primary interest was to investigate G × P interaction where population was classified by ancestry PCs. For this purpose, we used three designs of combinations of the three groups, i.e., POP1 + POP2 (Figure 1B), POP2 + POP3 (Figure 1C), and POP1 + POP3 (Figure 1D). To avoid any potential bias due to an imbalance in the sample size across populations, we made sample size consistent across POP1 and POP2 in the design of POP1 + POP2 by randomly selecting 7,500 individuals from the 17,000 white British individuals, which were used as POP1 in the downstream analyses.

We extracted genetic data including around 92 million imputed SNPs across autosomes from the UKBB for all the individuals of POP1, POP2 and POP3. Stringent QC was applied to the combined data across POP1, POP2 and POP3. The QC criteria were to exclude (1) all duplicated and non-autosomal SNPs, (2) SNPs with INFO score <0.6, (3) SNPs with call rate <0.95, (4) individuals with missing rate >0.05, (5) SNPs with Hardy-Weinberg equilibrium *p*-value < 0.0001, (6) SNPs with minor allele frequency <0.01, and (7) ambiguous SNPs with A/T or G/C alleles. We also retained HapMap3 SNPs only as they are reliable and robust to bias in estimating SNP-heritability and genetic correlation (The International HapMap 3 Consortium, 2010; Bulik-Sullivan et al., 2015; Tropf et al., 2017). Hereafter, 1,133,957 common SNPs remained for the G × P analyses. Moreover, we excluded one individual randomly selected from any pair with a genetic relationship >0.05 (see section “Statistical Models”) to avoid bias due to confounding by shared environment among close relatives. After the QC, the sample sizes of POP1, POP2, and POP3 were reduced to 7,487, 6,913 and 7,829.

### UKBB Phenotypes

For current UKBB resource, we have access to 496 variables whose data types are categorical (multiple), categorical (single), continuous, integer, date, text and time. Here, we focused on the variables of categorical (multiple), categorical (single), continuous and integer types, and categorized each variable as one of four value types: continuous, binary, ordered categorical and unordered categorical variable (Millard et al., 2019; Supplementary Table S1). Where a data field is measured at several time points we use the first occurrence only. It was noted that qualifications (data field 6138), a categorical (multiple) trait, was reorganized according to the underlying system (Guggenheim and Williams, 2016). Briefly, the original and unordered seven categories were reclassified and ordered as (1) none, (2) O-levels or CSEs, (3) A-levels, NVQ, HND, HNC or other professional qualification, and (4) college or university degree. Then the continuous, binary and ordered categorical variables were selected and used as the main phenotypes in G × P interaction analyses.

Among the 199 variables, we selected 128 variables as the main phenotypes (Supplementary Table S2) in our proposed model to estimate G × P interactions where population difference was inferred from the first and second PCs. The other variables were used to control confounding effects owing to sex, age, year of birth, genotype batch, and assessment center (basic confounders adjusted for all the main phenotypes; the first 20 PCs were also used as basic confounders to account for population stratification) and Townsend deprivation index, smoking status, alcohol consumptions and many other variables (additional cofounders adjusted for some relevant phenotypes) or excluded if they were not likely to affect any of the main phenotypes (see the note of Supplementary Table S2). It is noted that in this paper, for the covariates used as fixed effects in the models to correct mean difference across confounding factors, we used the term “confounders” to distinguish the covariates used in the RNM methods, i.e., PC1 and PC2 (see Section “Statistical Models”).

### Statistical Models

#### A Linear Mixed Model Without Considering G × P Interaction (Baseline Model)

A standard linear mixed model assuming no G × P interaction can be written as:

y=μ+g+e,

where **y** is an *n* × 1 vector of phenotypes with *n* being the sample size, **μ** is an *n* × 1 vector for fixed effects, **g** is an *n* × 1 vector of total genetic effects of the individuals with $g∼N\left(0,\text{A}\sigma_{g}^{2}\right)$ and **e** is an *n* × 1 vector of residual effects with $\sigma_{g}^{2}$, where $\sigma_{g}^{2}$ is the variance explained by all common SNPs and $\sigma_{e}^{2}$ is the residual variance. In the GREML context (Yang et al., 2010, 2011), **A** is a genomic relationship matrix (GRM) and **I** is an identity matrix. GRM can be estimated based on common SNPs across the genome and the elements of GRM can be defined as (VanRaden, 2008; Yang et al., 2010; Lee and Van der Werf, 2016):

$$A_{ij}=\frac{1}{L}\sum_{l=1}^{L}\frac{\left(x_{il}-2p_{l}\right)\left(x_{jl}-2p_{l}\right)}{var\left(x_{l}\right)},$$

where *L* is the number of all common SNPs (*L* = 1,133,957 in this study), *x*_*il*_ denotes the number of copies of the reference allele for the *l*th SNP of the *i*th individual, *x*_*l*_ denotes all the numbers of copies of the reference allele across all the individuals, and*p*_*l*_ denotes the reference allele frequency of the *l*th SNP.

The variance-covariance matrix of the observed phenotypes **(V)** is:

$$\text{V}=\text{A}\sigma_{g}^{2}+\text{I}\sigma_{e}^{2}.$$

The SNP-based heritability, the proportion of the additive genetic variance explained by the genome-wide SNPs over the total phenotypic variance, is then referred as:

$$h_{SNP}^{2}=\frac{\sigma_{g}^{2}}{\sigma_{y}^{2}}=\frac{\sigma_{g}^{2}}{\sigma_{g}^{2}+\sigma_{e}^{2}}.$$

The phenotypes with significant SNP-based heritability from this baseline model will subsequently be investigated for G × P interaction.

#### G × P RNM Method

In cases where G × P interaction exists across populations, the baseline model cannot account for heterogeneous genetic effects. We therefore applied RNM methods to detect heterogeneity across populations using the UKBB data. RNM and multivariate RNM (MRNM) have been demonstrated to perform better than the current state-of-the-art methods when detecting genotype-covariate and residual-covariate interactions in terms of simulation studies on type I error rate and power analyses (Ni et al., 2019). Here, we focus on G × P interaction by considering PCs as covariates in the RNM:

$$\text{y}=\mu +\text{g}+\text{e}=\mu +{\text{g}}_{0}+{\text{g}}_{1}\cdot \text{c}+\text{e},$$

where **y**, **μ**, **g** and **e** are the same defined in the baseline model above, **g**_0_ and **g**_1_ are *n* × 1 vectors of the zero- and first-order random regression coefficients, respectively, **c** is an *n* × 1 vector of covariate values of the *n* individuals (for which we used PC1 and PC2 values in this study). In the RNM, the random genetic effects, **g**, are regressed on the covariate gradient (reaction norm), which can be modeled with random regression coefficients, **g**_0_ and **g**_1_. This G × P RNM accounts for phenotypic plasticity and norms of reaction in response to different populations (represented by PC values) among samples.

The mathematical properties of variance-covariance structure between **g**_0_ and **g**_1_ allow us to verify whether estimates of the parameters are reasonable or not. Specifically, estimated values should be within a valid parameter space:

(1) $var\left({\hat{\text{g}}}_{0}\right)\geq 0$;
(2) $var\left({\hat{\text{g}}}_{1}\right)\geq 0$;
(3) $-\sqrt{var\left({\hat{\text{g}}}_{0}\right)var\left({\hat{\text{g}}}_{1}\right)}\leq cov\left({\hat{\text{g}}}_{0},{\hat{\text{g}}}_{1}\right)\leq \sqrt{var\left({\hat{\text{g}}}_{0}\right)var\left({\hat{\text{g}}}_{1}\right)}$.

The estimates which violated one of above criteria were excluded for follow-up analyses. We obtained a p-value to detect G × P interaction using a likelihood ratio test (LRT) that compared the goodness of fitness of two models (GREML and G × P RNM), penalizing the difference in the number of parameters between them.

We further tested if the significant G × P interactions were orthogonal (independent without confounding) to residual-population (R × P) interactions, i.e., residual heterogeneity across populations (Ni et al., 2019). Similarly, the R × P interaction can be detected by an R × P RNM:

$$\text{y}=\mu +\text{g}+\text{e}=\mu +\text{g}+{\text{e}}_{0}+{\text{e}}_{1}\cdot \text{c},$$

where e_0_ and ***e*_1_** are n × 1 vectors of the zero- and first-order random regression coefficients when residual effects, **e**, are regressed on the covariate, **c**, i.e., an *n* vector of PC1 or PC2. Furthermore, a full RNM model with both G × P and R × P interactions can be expressed as:

$$\text{y}=\mu +{\text{g}}_{0}+{\text{g}}_{1}\cdot \text{c}+{\text{e}}_{0}+{\text{e}}_{1}\cdot \text{c}.$$

Since the G × P and R × P models are nested within the full model, LRT comparing the full and R × P or G × P model with an appropriate degree of freedom can determine the significance of orthogonal G × P or R × P interaction (Ni et al., 2019). More details about RNM can be found elsewhere (Ni et al., 2019).

**FIGURE 2:**
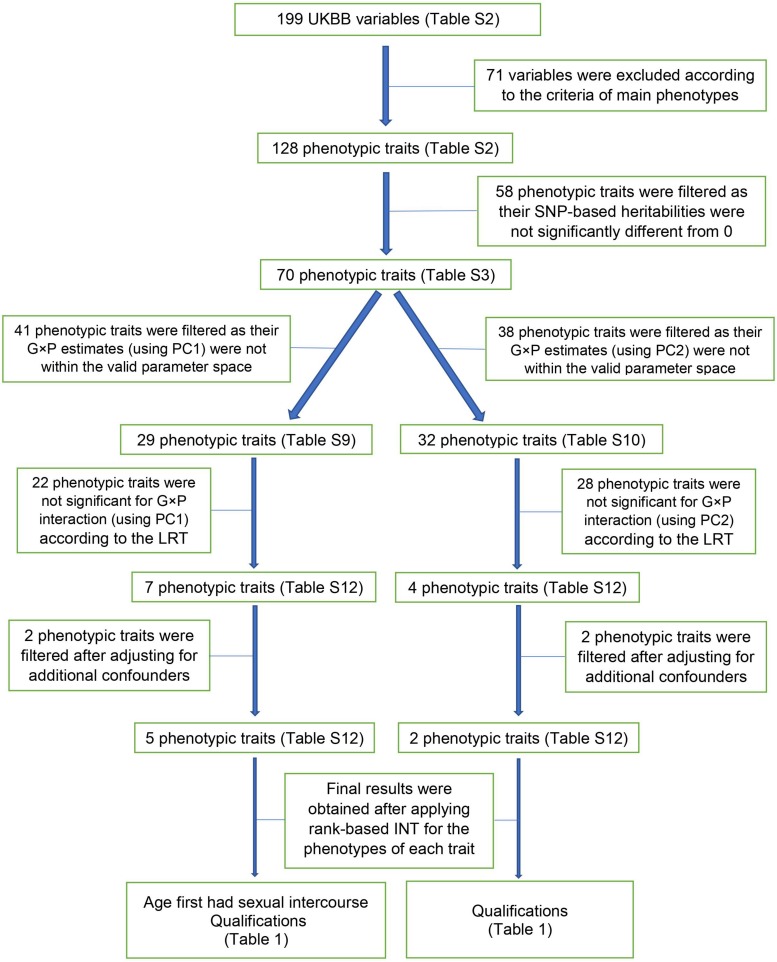
A flowchart of the G × P analysis on the design of POP1 + POP2.

For the analyses showing a significant G × P interaction, we used rank-based INT phenotypes to check explicitly if the significance was due to phenotypic heteroscedasticity or normality assumption violation (Robinson et al., 2017). The bias of RNM/MRNM estimates due to non-normality of phenotypic values can also be remedied by applying the rank-based INT (Ni et al., 2019). In short, the pipeline of our G × P analysis method is briefly as below: firstly, exclude the phenotypes with no significant heritability by GREML; secondly, for the remained phenotypes, exclude the ones with no significant result by LRT comparing RNM and GREML considering basic and additional confounders of fixed effects; thirdly, for the remained phenotypes, exclude the ones with no significant result after further considering robustness to normality assumptions of phenotypic values. We also presented a flowchart showing the pipeline of our G × P analysis on the design of POP1 + POP2 (Figure 2). All models described above (i.e., GREML, bivariate GREML, RNM, MRNM) can be fitted using software MTG2 (Lee and Van der Werf, 2016).

#### Spurious Signals Due to Selection or Collider Bias

We used the UKBB data that have only a 5.5% response rate, i.e., selection. Consequently, the resulting sample may not be representative of the UK population as a whole and the selection may be associated with some of the phenotypes in the UKBB, causing selection or collider bias (Swanson, 2012; Munafò et al., 2018). To test whether the G × P interaction effects detected by our method was genuine or spurious due to selection or collider bias, we conducted a series of simulation studies with phenotypes differentially selected for POP1 (white British) and POP2 (other white Europeans). If two or more phenotypic variables simultaneously influence the probability of participation of individuals in a study, then investigating associations between those variables in the selected sample may induce collider bias (Munafò et al., 2018). Therefore, we further considered the same selection model but including two traits to evaluate collider bias effects on the detection of G × P interaction across POP1 and POP2. The statistical models to test selection and collider bias can be found in Supplementary Text S1 or Munafò et al. (2018).

## Results

### Estimating SNP-Based Heritability for 128 Phenotypes

We first applied the standard GREML model to estimate $h_{SNP}^{2}$ for the 128 phenotypes across POP1 + POP2, POP2 + POP3 and POP1 + POP3, respectively. The phenotypes with significant $h_{SNP}^{2}$ (Supplementary Tables S3–S5) were further investigated for G × P interaction effects using our G × P RNM approach.

### Genetic and Residual Correlations Between Phenotypes and PCs

The main response (**y**) and environmental covariates (**c**) are not always uncorrelated, for which multivariate RNM accounting for (genetic and residual) correlations between **y** and **c** should be used (Ni et al., 2019). We examined if there were non-negligible genetic and residual covariances between the main phenotypes and covariate (PC1 or PC2) for the complex traits with significant heritabilities (Supplementary Tables S6–S8). All genetic and residual covariances estimated by bivariate GREML were not significantly different from zero, and thus we used univariate RNM to detect the G × P interaction effects with covariate PC1/PC2 for those phenotypes.

### G × P Interaction

For POP1 + POP2, we fit the data of the 70 phenotypes with significant $h_{SNP}^{2}$ by modeling the G × P RNM with covariates PC1 and PC2, respectively (Supplementary Tables S9, S10). We excluded those estimates, which were not within the valid parameter space (see Statistical models), from the follow-up statistical test analyses, resulting in 29 and 32 traits remaining for PC1 (Supplementary Table S9) and PC2 analyses (Supplementary Table S10). We examined if there was significant G × P interaction and obtained p-values based on LRT comparing the fit to the data of the G × P RNM and null model. Significance level was determined by Bonferroni multiple testing correction: 0.05/140 = 3.57E−4 for the 70 phenotypes with covariates PC1 and PC2. Supplementary Figure S1 show that significant G × P interactions were found for ten complex traits which are related to blood pressure (pulse rate, automated reading), bone-densitometry of heel (heel BMD T-score, automated; heel broadband ultrasound attenuation, direct entry; heel QUI, direct entry; heel BMD), diet (lamb/mutton intake), sexual factor (age at first sexual intercourse), sleep (sleep duration), smoking (ever smoked) and education (qualifications). For each of the ten traits, we further considered a multiple covariate model that fit PC1 and PC2 jointly (Supplementary Table S11). However, G × P interactions were less significant than those obtained using the single covariate model fitting PC1 or PC2 separately (Supplementary Figure S2), otherwise, the estimates were out of the valid parameter space. This was probably due the fact that there was collinearity between G × P interactions from PC1 and PC2.

In addition to the basic confounders for which the main phenotypes were initially adjusted (see Materials and methods), we further considered additional trait-specific confounders that might be relevant to some of traits (Supplementary Table S2), e.g., Townsend deprivation index, smoking status, alcohol drinker status, etc. After controlling for additional trait-specific confounders, the G × P interactions in POP1 + POP2 were still significant for bone-densitometry of heel (heel BMD T-score, automated; heel broadband ultrasound attenuation, direct entry; heel QUI, direct entry; heel BMD), age first had sexual intercourse and qualifications, whereas the signals disappeared for the other traits (Supplementary Table S12).

We examined the distribution of phenotypic values after controlling additional confounders of the six traits with significant G × P interactions (Supplementary Figure S3) and could not rule out the possibility that the interaction signals were due to non-normality (e.g., residual heteroscedasticity). We conducted the same analyses for the six traits using rank-based INT phenotypes (Table 1), which could control type I error rate due to a skewed and non-normal distribution of residual values (Ni et al., 2019). Indeed, phenotypic heteroscedasticity was remedied when using rank-based INT for the phenotypes of six traits as shown in Supplementary Figures S4–S9.

**TABLE 1:** Genetic variance, interaction variance and their covariance component estimates for six phenotypes across POP1 + POP2 with the covariates PC1 and PC2.

UKBBdata field	Phenotype	Covariate	*var*(*g*_0_) (SE)	*var*(*g*_1_) (SE)	*cov*(*g*_0_,*g*_1_) (SE)	*var*(*e*_0_) (SE)	*P*-value by LRT comparing with baseline model (DF = 2)
78	Heel bone mineral density	PC1	0.3151(0.0459)	0.0124(0.0110)	0.0013(0.0120)	0.6739(0.0456)	0.1586
	(BMD) T-score, automated	PC2	0.3187(0.0460)	−0.0008(0.0047)	−0.0037(0.0110)	0.6838(0.0450)	Excluded
3144	Heel Broadband ultrasound	PC1	0.2754(0.0454)	0.0094(0.0110)	0.0087(0.0120)	0.7161(0.0454)	0.0789
	attenuation, direct entry	PC2	0.2774(0.0454)	0.0006(0.0048)	−0.0024(0.0111)	0.7232(0.0450)	0.8987
3147	Heel quantitative ultrasound	PC1	0.3151(0.0459)	0.0124(0.0110)	0.0013(0.0120)	0.6739(0.0456)	0.1597
	index (QUI), direct entry	PC2	0.3187(0.0460)	−0.0009(0.0047)	−0.0037(0.0110)	0.6839(0.0450)	Excluded
3148	Heel bone mineral density	PC1	0.3070(0.0458)	0.0107(0.0109)	0.0046(0.0120)	0.6836(0.0455)	0.1315
	(BMD)	PC2	0.3106(0.0459)	−0.0016(0.0046)	−0.0069(0.0110)	0.6926(0.0450)	Excluded
2139	Age first had sexual	PC1	0.1006(0.0266)	0.0080(0.0078)	0.0203(0.0087)	0.8909(0.0290)	5.16E−05
	intercourse	PC2	0.1012(0.0266)	0.0110(0.0057)	−0.0015(0.0087)	0.8880(0.0286)	0.0097
6138	Qualifications	PC1	0.1194(0.0235)	0.0706(0.0103)	−0.0791(0.0090)	0.8124(0.0261)	9.21E−18
		PC2	0.1778(0.0214)	0.0360(0.0059)	0.0833(0.0081)	0.7885(0.0233)	2.22E−24

*The phenotypes were adjusted by basic plus additional confounders of fixed effects and transformed by rank-based INT. The G × P interaction signals of age first had sexual intercourse and qualifications were remained significant even after applying rank-based INT phenotypes, however, the other traits were not significant anymore. The estimates which were not within the valid parameter space are marked as “Excluded.” SE denotes standard error. DF denotes degree of freedom.*

For age first had sexual intercourse and qualifications that were shown to have significant G × P interactions, we further tested if the G × P interactions were orthogonal to R × P interactions, i.e., residual heterogeneity (see section “Materials and Methods”). Using the rank-based INT phenotypes adjusted for basic and additional confounders, we carried out an R × P model and a full model in which both G × P and R × P were fitted jointly. Subsequently, we conducted LRT to obtain p-values, comparing the full and nested models. A significant p-value from LRT between the full and R × P model indicates that G × P interaction is orthogonal to R × P interaction (see section “Materials and Methods” and Supplementary Table S13). For age first had sexual intercourse, although G × P or R × P interaction was significantly detected from the G × P or R × P model, it was shown that G × P interaction was not orthogonal to R × P (*p*-value = 0.88 for PC1 and 0.92 for PC2 in Supplementary Table S13). For qualifications, on the other hand, it was shown that the G × P and R × P interactions were statistically independent (*p*-value = 4.15E−05 for PC1 and 0.003 for PC2 in Supplementary Table S13).

For POP2 + POP3, we conducted analyses using the same procedure as in the analyses of POP1 + POP2. The POP3 individuals are very close to those in POP1 in terms of ancestry PC, but their ethnicities are not white British as in POP1 (see section “Materials and Methods” and Figure 1). Thirteen phenotypes demonstrated a significant genetic heterogeneity for covariate PC1 or PC2 as shown in Supplementary Tables S14, S15. After controlling for additional trait-specific confounders and transforming by rank-based INT (Supplementary Table S16), the results for age first had sexual intercourse (*p*-value = 7.86E−05 for PC1) and qualifications (*p*-value = 1.06E−15 for PC1) have demonstrated strong genetic heterogeneity signals (Table 2), which are consistent with our findings for POP1 + POP2. For qualifications, G × P interactions were significantly orthogonal to R × P interactions (*p*-value = 0.003 for PC1 in Supplementary Table S17). We also found significant results across POP2 + POP3 for anthropometric traits (waist circumference and weight) and diabetes diagnosed by doctor (Table 2). However, these phenotypes were not discovered across POP1 + POP2 with significant G × P interaction signals.

**TABLE 2:** Genetic variance, interaction variance and their covariance component estimates for six phenotypes across POP2 + POP3 with the covariates PC1 and PC2.

UKBB data field	Phenotype	Covariate	*var*(*g*_0_) (SE)	*var*(*g*_1_) (SE)	*cov*(*g*_0_,*g*_1_) (SE)	*var*(*e*_0_) (SE)	*P*-value by LRT comparing with baseline model (DF = 2)
48	Waist circumference	PC1	0.1802(0.0243)	0.0222(0.0069)	−0.0395(0.0079)	0.7990(0.0256)	2.92E−06
		PC2	0.1789(0.0243)	0.0076(0.0037)	0.0300(0.0078)	0.8147(0.0252)	0.0004
21002	Weight	PC1	0.2537(0.0252)	0.0209(0.0069)	−0.0328(0.0081)	0.7270(0.0257)	0.0002
		PC2	0.2529(0.0252)	0.0077(0.0040)	0.0219(0.0080)	0.7408(0.0252)	0.0252
2443	Diabetes diagnosed by	PC1	0.1688(0.0203)	0.0259(0.0070)	−0.0015(0.0077)	0.7901(0.0218)	6.65E−11
	doctor	PC2	0.1734(0.0204)	0.0162(0.0051)	−0.0005(0.0076)	0.7966(0.0219)	3.73E−08
2139	Age first had sexual	PC1	0.0936(0.0258)	0.0267(0.0086)	−0.0072(0.0087)	0.8795(0.0283)	7.86E−05
	intercourse	PC2	0.0933(0.0258)	0.0153(0.0056)	0.0112(0.0086)	0.8918(0.0278)	0.0071
6138	Qualifications	PC1	0.0937(0.0264)	0.0324(0.0094)	0.0159(0.0091)	0.8715(0.0287)	1.06E−15
		PC2	0.1139(0.0267)	0.0150(0.0057)	0.0137(0.0086)	0.8713(0.0285)	0.0162

*The phenotypes were adjusted by basic plus additional confounders of fixed effects and transformed by rank-based INT. The results for behavioral phenotypes age first had sexual intercourse and qualifications have demonstrated strong genetic heterogeneity signals, which are consistent with the findings for POP1 + POP2. The significant results for anthropometric traits (waist circumference and weight) and diabetes diagnosed by doctor were also detected. However, these phenotypes were not discovered across POP1 + POP2 with significant G × P interaction signals. SE denotes standard error. DF denotes degree of freedom.*

We also performed the same analyses on POP1 + POP3, which is not a diverse population group as POP1 + POP2 or POP2 + POP3, and thus was used as a negative control group (see section “Materials and Methods”). For several traits showing significant heterogeneous signals with covariate PC1 or PC2 after Bonferroni correction (see Supplementary Tables S18, S19), we further examined them by adding stringent confounders to correct for fixed effects and applying rank-based INT. The final results included no significant G × P interaction across POP1 + POP3 (see Supplementary Tables S20, S21).

For the categorical phenotype qualifications, there were various ways to convert the seven UKBB categories into a continuous or a binary measure (Okbay et al., 2016; Gazal et al., 2017; Lee et al., 2018). Following a previous study (Okbay et al., 2016), we transformed the multiple categories (data fields: 6138.0.0 to 6138.0.5) into an educational year measure (Supplementary Table S22). Based on this continuous phenotypic measure, we found significant genetic heterogeneity across POP1 + POP2 and POP2 + POP3 but no signal across POP1 + POP3 (Supplementary Table S23), which was consistent with our results obtained using four-level categories. We also examined G × P interactions for qualifications based on two types of binary measures (highest educational attainment versus other levels, and lowest educational attainment versus other levels) (Gazal et al., 2017). The results were consistent with those obtained using four-level qualifications, except that an unexpected significant signal across POP1 + POP3 for covariate PC1 was detected based on the binary measure of “college or university degree” versus other six categories (Supplementary Table S24).

### The Findings Are Robust to Selection or Collider Bias

We examined the distribution of phenotypic values for age first had sexual intercourse and qualifications in which G × P interactions were consistently detected from both POP1 + POP2 and POP2 + POP3 (Supplementary Tables S25, S26). The distribution of age first had sexual intercourse is similar across POP1, POP2 and POP3. However, for qualifications, it is apparently shown that the subjects in POP2 and POP3 (other white Europeans) have higher qualification levels than those in POP1 (white British). Moreover, it is likely that the individuals in POP1 have higher educational levels than the general population of United Kingdom because individuals with higher educational levels are more likely to response to surveys from UKBB (Munafò et al., 2018).

Our simulation studies testing for detecting spurious heterogeneity across POP1 and POP2 with multiple scenarios varying the level of selection odds ratios (see Supplementary Text S1 for details) have verified that (1) both G × P RNM and bivariate GREML are robust to the selection bias when using the same selection odds ratio across populations (Table 3); (2) only bivariate GREML is robust against the selection bias when using different selection odds ratios across populations (Table 3); (3) bivariate GREML is robust against the collider bias when estimating genetic correlation between POP1 and POP2, however, it generates biased estimation of genetic correlation between the two traits (Table 4). It is noted that the level of selection odds ratios used in simulations is likely to reflect the real situation of qualifications, i.e,. different selection pressure between POP1 and POP2 in UKBB (see Supplementary Text S1 and Supplementary Table S27).

**TABLE 3:** Simulation study results for selection bias on the phenotype Y across POP1 + POP2.

Selection scenarios in POP1 + POP2	Type I error rate by G × P RNM with PC1	Type I error rate by bivariate GREML	100 estimated genetic correlations	
			Mean	*SE*
*OR*_*POP1,Y*_ = 1, *OR*_*POP2,Y*_ = 1	5%	0%	0.9722	0.0145
*OR*_*POP1,Y*_ = 1, *OR*_*POP2,Y*_ = 2	55%	2%	0.9876	0.0166
*OR*_*POP1,Y*_ = 2, *OR*_*POP2,Y*_ = 2	1%	0%	1.0245	0.0160
*OR*_*POP1,Y*_ = 2, *OR*_*POP2,Y*_ = 3	64%	6%	0.9882	0.0202

*Different odds ratio combinations (OR_POP1,Y_ andOR_POP2,Y_) generated phenotypic values in POP1 + POP2 with different selection bias levels. Type I error rates based on 100 simulation replicates were examined by G × P RNM and bivariate GREML respectively. The genetic correlations of the phenotype between POP1 and POP2 were estimated by bivariate GREML. The results by G × P RNM indicated high type I error rates for selection scenarios of OR_POP1,Y_ = 1, OR_POP2,Y_ = 2 and OR_POP1,Y_ = 2, OR_POP2,Y_ = 3. But for same selection pressures (OR_POP1,Y_ = 1, OR_POP2,Y_ = 1 and OR_POP1,Y_ = 2, OR_POP2,Y_ = 2), G × P RNM can effectively control false positive rate. Type I error rates assessed by bivariate GREML were controlled for all scenarios and the estimated genetic correlations were not significantly different from one for all selection scenarios. Here, we consider 0.05 as a significance level for controlling type I error rate. SE denotes standard error.*

**TABLE 4:** Simulation study results for collider bias on two phenotypes Y and Z across POP1 + POP2.

Selection scenarios with collider bias in POP1 + POP2	Type I error rate	Estimated genetic correlations of the phenotype Y between POP1 and POP2		Estimated genetic correlations between Y and Z on selected POP1 + POP2	
		
		Mean	SE	Mean	SE
*OR*_*POP1,Y*_ = 2, *OR*_*POP1,Z*_ = 2, *OR*_*POP2,Y*_ = 3, *OR*_*POP2,Z*_ = 2	1%	1.0141	0.0189	−0.2516	0.0032
*OR*_*POP1,Y*_ = 2, *OR*_*POP1,Z*_ = 2, *OR*_*POP2,Y*_ = 3, *OR*_*POP2,Z*_ = 3	2%	1.0220	0.0165	−0.2942	0.0031
*OR*_*POP1,Y*_ = 2, *OR*_*POP1,Z*_ = 3, *OR*_*POP2,Y*_ = 3, *OR*_*POP2,Z*_ = 3	2%	1.0091	0.0187	−0.3415	0.0036

*Different odds ratio combinations (OR_POP1,Y_, OR_POP2,Y_, OR_POP1,Z_, and OR_POP2,Z_) generated phenotypes in POP1 + POP2 with different selection bias levels. Type I error rates based on 100 simulation replicates were examined through estimated genetic correlations of the phenotype Y between POP1 and POP2 by bivariate GREML. Type I error rates under the null hypothesis that genetic correlation of 1 implies no interaction were controlled for these combinations (<5%), and meanwhile, significant negative genetic correlations between the two simulated phenotypes Y and Z demonstrated strong collider bias signal in selected POP1 + POP2. Here, we assume that the phenotype Z involves a sum of collider bias effects across all other traits on the main response Y. Here, we consider 0.05 as a significance level for controlling type I error rate. SE denotes standard error.*

For age first had sexual intercourse and qualifications, we also confirmed our findings using bivariate GREML, a robust approach against selection bias (Table 5). As confirmed by the bivariate GREML, it was not likely that the findings for qualifications were spurious because of selection and collider bias. This was also evidenced by the fact that G × P RNM detected a significant interaction signal from POP2 + POP3, noting that POP2 and POP3 were similarly distributed for qualifications (see Supplementary Table S26). Similarly, the findings for age first had sexual intercourse were mostly robust whether using RNM or bivariate GREML except that there was no signal for POP1 + POP2 when using the bivariate GREML, probably due to the lack of power. It was noted that the phenotypic distributions of age first had sexual intercourse were very similar across POP1, POP2 and POP3 (Supplementary Table S25).

**TABLE 5:** Genetic correlation estimates between population groups (POP1, POP2, and POP3) by bivariate GREML for two phenotypes.

Phenotype	Genetic correlation between POP1 and POP2			Genetic correlation between POP2 and POP3			Genetic correlation between POP1 and POP3		
	Estimate	*SE*	*P*-value	Estimate	*SE*	*P*-value	Estimate	*SE*	*P*-value
Qualifications	0.2554	0.2223	8.09E−04	0.4795	0.1550	7.85E−04	0.5676	0.2743	0.1149
Age first had sexual intercourse	0.7418	0.3984	0.5169	0.0491	0.2284	3.14E−05	1.2176	0.3629	0.5488

*The phenotypes were adjusted by basic plus additional confounders of fixed effects and transformed by rank-based INT. The bivariate GREML results for qualifications indicated a significant genetic heterogeneity between POP1 and POP2 (p-value = 8.09E−04), and between POP2 and POP3 (p-value = 7.85E−04), but showed no genetic heterogeneity between POP1 and POP3. These results were consistent with our findings from the G × P RNM. For age first had sexual intercourse, the bivariate GREML detected a significant heterogeneity between POP2 and POP3 (p-value = 3.14E−05), however, there was no interaction signal between POP1 and POP3 (as expected). Unexpectedly, the bivariate GREML failed to find genetic heterogeneity across POP1 + POP2 although RNM provided a significant signal. SE denotes standard error. P-value was obtained through a Wald test under a null hypothesis that genetic correlation equals to 1.*

## Discussion

Previous results (Maier et al., 2015; Robinson et al., 2015; Yang et al., 2015; Tropf et al., 2017) were more likely to reflect heterogeneous genetic effects across nations or cohorts rather than populations as those designs were evidently confounded with country-specific factors (e.g., trait definition and measurement, cultural and societal difference). In this study, we focused on populations and proposed the new concept “genotype-population interaction” in which population is defined by the first and second ancestry PCs (each individual has a unique PC value). Using the RNM with whole-genome data from the UKBB, we have demonstrated significant G × P interaction effects for qualifications and age first had sexual intercourse across populations. Our findings corroborate the results in Tropf et al. (2017) who reported that behavioral phenotypes (education and human reproductive behavior) have significant G × E interactions across populations. For anthropometric phenotypes, height and BMI, our G × P RNM model did not detect any significant interaction signals (Yang et al., 2015). However, the analyses of another two anthropometric traits (waist circumference and weight) have demonstrated significant genetic heterogeneity across the POP2 + POP3 group (other white Europeans). Actually, the results by Tropf et al. (2017) across seven populations have also revealed significant G × E interaction for BMI although the heterogeneity is not strong as for education and reproductive behaviors. Robinson et al. (2015) also reported that, for BMI, environmental differences across Europe masked genetic differentiation. Thus, these findings may be consistent for some anthropometric phenotypes when using diverse European ancestry populations. The previous results (Maier et al., 2015; Robinson et al., 2015; Yang et al., 2015; Tropf et al., 2017) were based on data collected from multiple countries. Therefore, various trait definitions in phenotypic measure and genetic measurement errors across countries may generate artificial heterogeneity. In our study, however, we used the UKBB data that have less cross-country factors and confounders. The phenotypic definitions and measurement of complex traits in the UKBB samples are well standardized and calibrated. Moreover, UKBB utilized uniform standards of imputation and quality control for genotype data. Therefore, our results may provide more reliable estimations of G × P interaction effects across populations.

From the POP2 + POP3 analyses, we also found a significant G × P interaction for diagnosis of diabetes that is a binary response variable. As the RNM has not been explicitly verified for binary traits, we also used bivariate GREML to estimate the genetic correlation between POP2 and POP3 for this disease trait and found no significant signal for genetic heterogeneity (estimate is 0.7988, *SE* = 0.2044, *p*-value = 0.3249). This might be due to the fact that there was no genuine interaction effects or that the bivariate GREML was simply underpowered. For the two binary measuring ways of qualifications (lowest educational attainment versus other levels, and highest educational attainment versus other levels), we also used bivariate GREML to examine genetic correlations between POP1, POP2 and POP3 (Supplementary Table S28). The results for the binary phenotype of “none of the above” versus other six educational categories demonstrated significant genetic heterogeneity between POP1 and POP2 (*p*-value = 5.58E−05) and between POP2 and POP3 (*p*-value = 7.59E−05) but no significant signal between POP1 and POP3 (*p*-value = 0.0619), which were consistent with those obtained from the main analyses. For the binary data of “college or university degree” versus other six categories, the bivariate GREML indicated a marginally significant heterogeneity between POP1 and POP2 (*p*-value = 0.035) and no significant signal between POP2 and POP3 (*p*-value = 0.494), and POP1 and POP3 (*p*-value = 0.94). The reason that the genetic heterogeneity became weaker or disappeared is probably due to the fact that the bivariate GREML has less power compared to the RNM approach, and the phenotype categories reduced from four to two levels.

Our results imply that causal variants at multiple loci may not be universal but rather specific to populations for some complex traits. The results on qualifications across POP1 + POP2 suggested that G × P interaction might be a reason for attenuation of SNP-based heritability when using data from different populations (see Supplementary Text S2 and Supplementary Table S29), which are agreed with Tropf et al. (2017). This missing or hidden heritability issue (Witte et al., 2014) can produce lower predictive power of polygenic risk scores from large GWAS (usually generated from meta-analyses of different populations) compared with single homogenous population since the reference heritability obtained from the meta-analyses among several populations is smaller than that obtained from single homogenous population (de Vlaming et al., 2017). Therefore, our findings suggest that large homogeneous population data sources (e.g., around 400,000 white British individuals in the UKBB) should be used to conduct polygenic risk prediction for some specific traits such as human behaviors.

The current methods used for estimating G × E (or G × P) interactions, e.g., random regression (RR)-GREML (Jarquín et al., 2014) and GCI-GREML (Tropf et al., 2017), require that the main response should be stratified into multiple discrete groups according to covariate levels even for a continuous covariate (Maier et al., 2015). However, the arbitrary grouping ignores the difference of covariate values for the individuals within each group, and results in some loss of information. In contrast, the RNM allows us to fit a continuous covariate representing individuals uniquely (e.g., PC) in the model and produces unbiased estimates (Ni et al., 2019). In our results, bivariate GREML which labels the individuals into two discrete groups (POP1 and POP2) failed to find genetic heterogeneity for age first had sexual intercourse (Supplementary Table S27), while RNM detected significant G × P interaction across POP1 + POP2 (see Table 1). It may imply that G × P RNM is more powerful as it uses individual-level information represented by PC across populations, while bivariate GREML ignores such information within each stratified group. However, on the other hand, RNM may suffer from the selection bias when using different selection odds ratios across populations (Table 3) while bivariate GREML is robust against such selection and collider bias (Tables 3, 4). It is noted that bivariate GREML requires pre-defined population labels (e.g., self-reported ethnicities).

Residual-covariate interaction may result in heterogeneous residual variances across different covariate values, thus it is necessary to examine and distinguish genotype-covariate and residual-covariate interactions (Ni et al., 2019). Our results (Supplementary Tables S13, S17) provided cogent evidence of G × P and R × P interaction effects, which are (partially) independent without confounding, across populations for qualifications. However, for age first had sexual intercourse, there was no evidence showing that G × P interaction was orthogonal to R × P interaction from LRT comparing the full and nested models. Therefore, we could not rule out the possibility that the significant signal was mainly because of residual heterogeneity across populations. In order to disentangle G × P interaction from R × P interaction, the magnitude of G × P interaction should be large (e.g., qualifications) or sample size may have to be increased.

There are several limitations in this study. Firstly, we examined G × P interaction across populations using three data designs (POP1 + POP2 and POP2 + POP3 as primary data, and POP1 + POP3 as a negative control), in which population is referred to the first and second ancestry PCs. As POP1 and POP3 are very close in terms of PCs, the individuals in the two primary groups POP1 + POP2 and POP2 + POP3 have common population structures (Figure 1). But both groups involve in different white ethnic backgrounds, i.e., POP1 may be closer to native British and POP2/POP3 is more likely to be descended from recent immigrants from many other European nations. Therefore, for our data designs, we cannot rule out the possibility that G × P interaction was confounded with immigration-specific factors such as socioeconomic attainment, social relations and cultural beliefs (Drouhot and Nee, 2019). We also notice that, in the UKBB data source, there are numerous samples with other ethnicities (e.g., Indian, Caribbean, and African), thus future studies using our approach may aim to detect genotype-ethnicity interaction, which may uncover challenges for investigations into the genetic architecture of phenotypes across various ethnicities. Secondly, population defined by PCs in this study or by discrete groups in others (Yang et al., 2015; Tropf et al., 2017) includes both environmental and genetic information for individuals, thus the G × P interaction may not merely embody G × E interaction but also contains confounded genotype-by-genotype (G × G) interaction across populations. It may become a new challenge in the future to distinguish G × E and G × G in studies of genetic heterogeneity across populations. Thirdly, the sample size for people with other white ethnicity in UKBB (i.e., the sum of POP2 and POP3) is not large, thus the study may lack power for phenotypes with small SNP-based heritability such as behavioral traits. The phenotypes without significant heritability in the current samples were not investigated for G × P interaction, however, if boosting statistical power for those phenotypes, there may be new findings for heterogeneity across populations. Fourthly, the simulations on selection bias have demonstrated that the G × P RNM is not robust for data across populations with different selection odds ratios (see Table 3). Thus our approach is more preferable and restricted to data without selection bias or with the same selection pressure for populations. Finally, for genotypic information used in this study, we only examined common SNPs (minor allele frequency > 0.01). However, a recent study (Wainschtein et al., 2019) reported that the missing heritability for height and BMI may be explained by rare genetic variants accessed from whole-genome sequence data. Therefore, can rare population-specific variants increase our understanding of genetic heterogeneity across populations? Further research is required to answer this question.

In conclusion, the main findings in our study demonstrated a significant population genetic heterogeneity for behavioral traits (age first had sexual intercourse and qualifications). Our study provided a paradigm shift tool in investigating genetic heterogeneity across populations. The new concept of G × P interaction with the use of ancestry PC is more plausible in explaining the genetic architecture of complex traits across heterogeneous populations. The G × P interaction effects on age first had sexual intercourse and qualifications were found by a powerful approach based on technically homogeneous data (free of genetic measurement errors and cohort/country confounding factors), and these findings were validated in both data designs POP1 + POP2 and POP2 + POP3. The analyses performed in this study can be applied to dissect the genetic architecture of complex traits and diseases across populations, and the results from these analyses will provide important information and suggestion for studies of polygenic risk prediction across Europeans.

## Data Availability Statement

Publicly available datasets were analyzed in this study. This research has been conducted using the UK Biobank Resource. UK Biobank (http://www.ukbiobank.ac.uk) Research Ethics Committee (REC) approval number is 11/NW/0382. Our reference number approved by UK Biobank is 14575.

## Author Contributions

CY and SL conceived and initiated the project. SL supervised and directed the study. CY and GN quality-controlled the data. CY analyzed the data and drafted the manuscript. JW provided key statistical advice. All authors revised the manuscript and approved the final version of the manuscript.

## Conflict of Interest

The authors declare that the research was conducted in the absence of any commercial or financial relationships that could be construed as a potential conflict of interest.
