# Supplementary material for: Detecting Genotype-Population Interaction Effects by Ancestry Principal Components
Source: Front Genet. 2020 Apr 21;11:379. doi: 10.3389/fgene.2020.00379 (PMC7186421; doi:10.3389/fgene.2020.00379)
Supplement: Supplementary file 1 [file Data_Sheet_1.PDF]

Detecting genotype-population interaction effects by ancestry principal components

Supplementary Material

This file includes two supplementary texts, 9 figures and 29 tables.

Text S1. Simulation study on selection and collider bias.

Statistical models for selection and collider bias

A selection model using a logistic regression with a trait Y can be written as

$$\text{logit}(\mathbf{p}) = \ln(\frac{\mathbf{p}}{1-\mathbf{p}}) = \boldsymbol{\mu} + \ln(OR_{\text{POP1, Y}}) \cdot \mathbf{y} \qquad \text{for POP1}$$

and

$$\text{logit}(\mathbf{p}) = \ln(\frac{\mathbf{p}}{1-\mathbf{p}}) = \boldsymbol{\mu} + \ln(OR_{\text{POP2, Y}}) \cdot \mathbf{y} \qquad \text{for POP2}$$

where **p** is a vector of participation probabilities in a study (e.g., UKBB questionnaire survey) for all individuals, **μ** is an overall mean vector which regulates the response rate, **y** is a vector of phenotypic values of the trait Y, $OR_{POP1,Y}$ and $OR_{POP2,Y}$ are selection odds ratios for POP1 and POP2, respectively. Then the sample selection bias can be simulated with varying selection odds ratios.

One hundred replicates of phenotypic values of the trait Y on POP1+POP2 (14,400 individuals) were simulated under a baseline model (GREML) that assumes no G×P interaction: **y** = **g** + **e**, where the variance-covariance structure between **g** and **e** was $\begin{pmatrix} 0.5 & 0 \\ 0 & 0.5 \end{pmatrix}$. For each replicate, to avoid insufficient statistical power, we set **μ** as a vector of zeros which simulates a response rate of 50%. Letting us divide **y** and **p** into subsets according to specific populations (i.e. $\mathbf{y}_1$ and $\mathbf{p}_1$ for POP1 and $\mathbf{y}_2$ and $\mathbf{p}_2$ for POP2), we obtain the participation probability for each individual as

$$\mathbf{p}_1 = \frac{1}{1+\exp(-\ln(OR_{\text{POP1, Y}}) \cdot \mathbf{y}_1)} \qquad \text{for POP1}$$

, and

$$\mathbf{p}_2 = \frac{1}{1+\exp(-\ln(OR_{\text{POP2, Y}}) \cdot \mathbf{y}_2)} \qquad \text{for POP2.}$$

Then, individuals in each population are selected based on the participation probability. Specifically, we generate a uniform distribution vector $\mathbf{u}_1$ on (0, 1) with sample size of POP1, and compare the values of corresponding components in $\mathbf{p}_1$ and $\mathbf{u}_1$. The individuals having larger values in $\mathbf{p}_1$ than in $\mathbf{u}_1$ are assumed to participate in this study. Similarly, we can select individuals in POP2 by comparing $\mathbf{p}_2$ with a random number drawn from a uniform

distribution (0, 1). Different combinations of selection odds ratios for POP1 and POP2 (e.g., $OR_{POP1,Y}$= 1 and $OR_{POP2,Y}$= 2) will generate selection bias associated with phenotypic values in the POP1+POP2 groups.

Since the phenotypic data was simulated under the null model, a significant G×P interaction detected from LRT comparing G×P RNM versus GREML was a type I error (false positive). This allowed us to investigate the type I error rate of G×P interaction due to selection bias attributed to various selection pressures (odds ratios) on POP1 and POP2. Using the same simulated data, we also applied a bivariate GREML (Lee et al, 2012) to test if estimated genetic correlation between POP1 and POP2 was significantly different from 1 (i.e. evidence of G×P interaction across POP1 and POP2) (Falconer and Mackay, 1996). This allowed us to assess the type I error rate of G×P interaction when using the bivariate GREML.

To evaluate collider bias effects, the selection model with two traits Y and Z can be written as

$$\text{logit}(\mathbf{p}) = \ln(\frac{\mathbf{p}}{1-\mathbf{p}}) = \boldsymbol{\mu} + \ln(OR_{\text{POP1, Y}}) \cdot \mathbf{y} + \ln(OR_{\text{POP1, }Z}) \cdot \mathbf{z} \qquad \text{for POP1}$$

, and

$$\text{logit}(\mathbf{p}) = \ln(\frac{\mathbf{p}}{1-\mathbf{p}}) = \boldsymbol{\mu} + \ln(OR_{\text{POP2, Y}}) \cdot \mathbf{y} + \ln(OR_{\text{POP2, }Z}) \cdot \mathbf{z} \qquad \text{for POP2}$$

where **z** is a vector of phenotypic values of the trait Z, $OR_{POP1,Z}$ and $OR_{POP2,Z}$ are selection odds ratios with the trait Z for POP1 and POP2. The magnitude of collider bias depends on the levels of selection odds ratios for the two phenotypes.

We simulated 100 replicates of phenotypic values of the trait Z on POP1+POP2 under the null model of no G×P interaction: **z** = **α** + **β**, where the variance-covariance structure between **α** and **β** is $\begin{pmatrix} 0.5 & 0 \\ 0 & 0.5 \end{pmatrix}$. Since genetic components **g** and **α** are uncorrelated and residual components **e** and **β** are uncorrelated, the phenotypic variable **z** and previous simulated **y** = **g** + **e** are totally independent, but after selection we expect that the two variables will be associated because of a collider. Letting us divide **z** into subsets according to specific populations (i.e. $\mathbf{z}_1$ for POP1 and $\mathbf{z}_2$ for POP2), the individuals can be selected based on

$$\mathbf{p}_1 = \frac{1}{1 + \exp(-\ln(OR_{\text{POP1, Y}}) \cdot \mathbf{y}_1 - \ln(OR_{\text{POP1, }Z}) \cdot \mathbf{z}_1)} \qquad \text{for POP1}$$

and

$$\mathbf{p}_2 = \frac{1}{1 + \exp(-\ln(OR_{\text{POP2, Y}}) \cdot \mathbf{y}_2 - \ln(OR_{\text{POP2, }Z}) \cdot \mathbf{z}_2)} \qquad \text{for POP2.}$$

Similarly, we can select individuals in POP1 or POP2 by comparing $\mathbf{p}_1$ or $\mathbf{p}_2$ with a random number drawn from a uniform distribution (0, 1). Therefore, in terms of collider bias, different combinations of selection odds ratios for different traits and populations (e.g., $OR_{POP1,Y}$= 2, $OR_{POP2,Y}$= 3, $OR_{POP1,Z}$= 2 and $OR_{POP2,Z}$= 2) will generate collider bias in the POP1+POP2 groups. Similarly, we can examine G×P interaction by type I error rate analysis using G×P RNM and bivariate GREML methods and assess collider bias effects for the two methods.

Simulation study on selection bias

We conducted a series of simulation studies by examining different odds ratio combinations for selection in POP1 and POP2 to evaluate influence of detecting G×P interaction due to selection bias (see Materials and Methods). For the 100 replicates of phenotype simulated under a null model, we adjusted the values by first 20 PCs and ethnicity (POP1 or POP2) as fixed effects and transformed by rank-based INT. We then used G×P RNM with covariate PC1 and baseline GREML models to fit the 100 simulated replicates. Since the data was simulated under a null model, we consider a significant G×P interaction signal detected through LRT of G×P RNM versus GREML as a type I error (false positive). The results as shown in Table 3 indicated high type I error rates (> 50%) for selection scenarios of $OR_{POP1,Y}$= 1, $OR_{POP2,Y}$= 2 and $OR_{POP1,Y}$ = 2, $OR_{POP2,Y}$= 3. But for same selection pressures ($OR_{POP1,Y}$= 1, $OR_{POP2,Y}$= 1 and $OR_{POP1,Y}$= 2, $OR_{POP2,Y}$= 2), G×P RNM can effectively control false positive rate. Type I error was also evaluated through genetic correlation estimated by bivariate GREML for the phenotype between POP1 and POP2 with the null assumption that genetic correlation = 1 implies no heterogeneity. As shown in Table 3, type I error rates assessed by bivariate GREML were controlled for all scenarios and the estimated genetic correlations were not significantly different from one for all selection scenarios.

To verify whether the selection odds ratios we simulated are comparable to real situation (qualifications), we performed linear regression including 1 (representing POP1) and 2 (representing POP2) as independent variable and educational levels as dependent variable on real data of qualifications across POP1+POP2. Similarly, we also performed 100 linear regressions for the 100 replicates simulated with selection odds ratio combination of $OR_{POP1,Y}$ = 2 and $OR_{POP2,Y}$ = 3. We then examined R-squared and p-values obtained from linear regressions for real data of qualifications and simulation data (Table S27). The regression R-squared values of simulations were similar to those of real data, which indicated that selection bias by $OR_{POP1,Y}$ = 2 and $OR_{POP2,Y}$ = 3 in simulation has been sufficient to reflect realistic situation of educational levels across POP1+POP2 in UKBB. Therefore, our simulation studies have verified that the G×P interaction of qualifications across populations we detected may not be due to selection bias at this scenario.

Simulation study on collider bias

We also performed simulation studies to evaluate collider bias effects on G×P interaction detected by bivariate GREML (see Subjects and Methods) as shown in Table 4. Type I error rates under the null hypothesis that genetic correlation of 1 implies no interaction were controlled for several combinations of $OR_{POP1,Y}$, $OR_{POP2,Y}$, $OR_{POP1,Z}$ and $OR_{POP2,Z}$, and meanwhile, significant negative genetic correlations between the two simulated phenotypes Y and Z demonstrated strong collider bias signal in selected POP1+POP2. Here we assume that the phenotype Z involves a sum of collider bias effects across all other traits on the main response Y. We further examined R-squared and p-values obtained from linear regressions for those simulation data with different collider bias levels (Table S27). The similar R-squared values indicated that our simulated collider bias settings have been sufficient to represent real situation of qualification levels. The results implied that collider bias may not be a cause for significant G×P interaction of qualifications across POP1+POP2.

Text S2. Hidden heritability

For the significant traits qualifications and age first had sexual intercourse, we examined SNP-based heritabilities estimated by GREML and G×P RNM (see Table S28). The

phenotypic values were adjusted for basic and additional confounders of fixed effects and transformed using rank-based INT. For POP1+POP2, the SNP-based heritability for qualifications estimated by G×P RNM increased by 28% (from 0.0998 to 0.1281) and 84% (from 0.0998 to 0.1840) with covariate PC1 and PC2, compared to those estimated by GREML. But there was no such apparent increase of estimated SNP-based heritability for POP2+POP3 and POP1+POP3 when comparing GREML and G×P RNM.

Fig S1 (A)

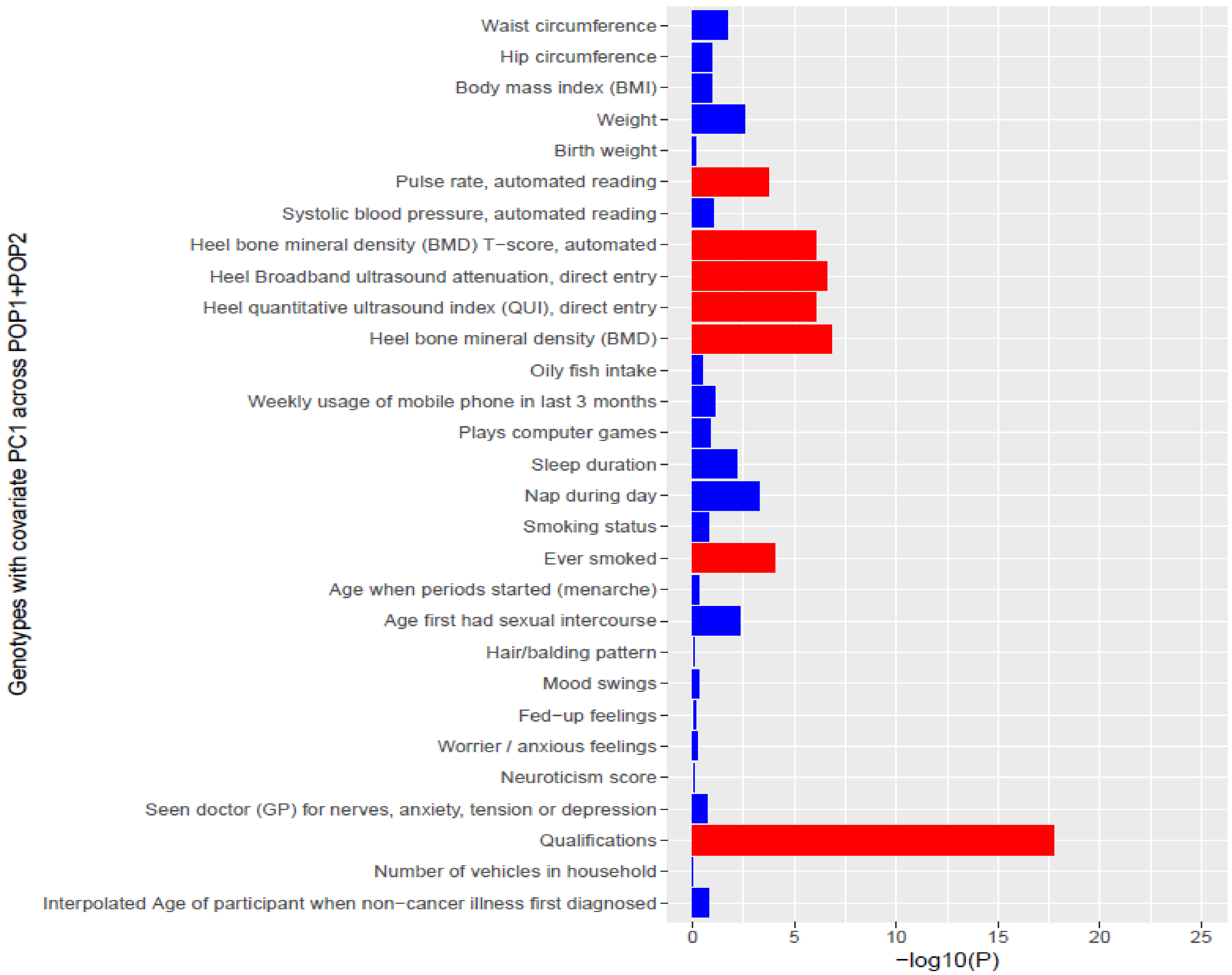

Genotypes with covariate PC1 across POP1+POP2
Waist circumference
Hip circumference
Body mass index (BMI)
Weight
Birth weight
Pulse rate, automated reading
Systolic blood pressure, automated reading
Heel bone mineral density (BMD) T−score, automated
Heel Broadband ultrasound attenuation, direct entry
Heel quantitative ultrasound index (QUI), direct entry
Heel bone mineral density (BMD)
Oily fish intake
Weekly usage of mobile phone in last 3 months
Plays computer games
Sleep duration
Nap during day
Smoking status
Ever smoked
Age when periods started (menarche)
Age first had sexual intercourse
Hair/balding pattern
Mood swings
Fed−up feelings
Worrier / anxious feelings
Neuroticism score
Seen doctor (GP) for nerves, anxiety, tension or depression
Qualifications
Number of vehicles in household
Interpolated Age of participant when non−cancer illness first diagnosed
0
5
10
15
20
25
−log10(P)

Fig S1 (B)

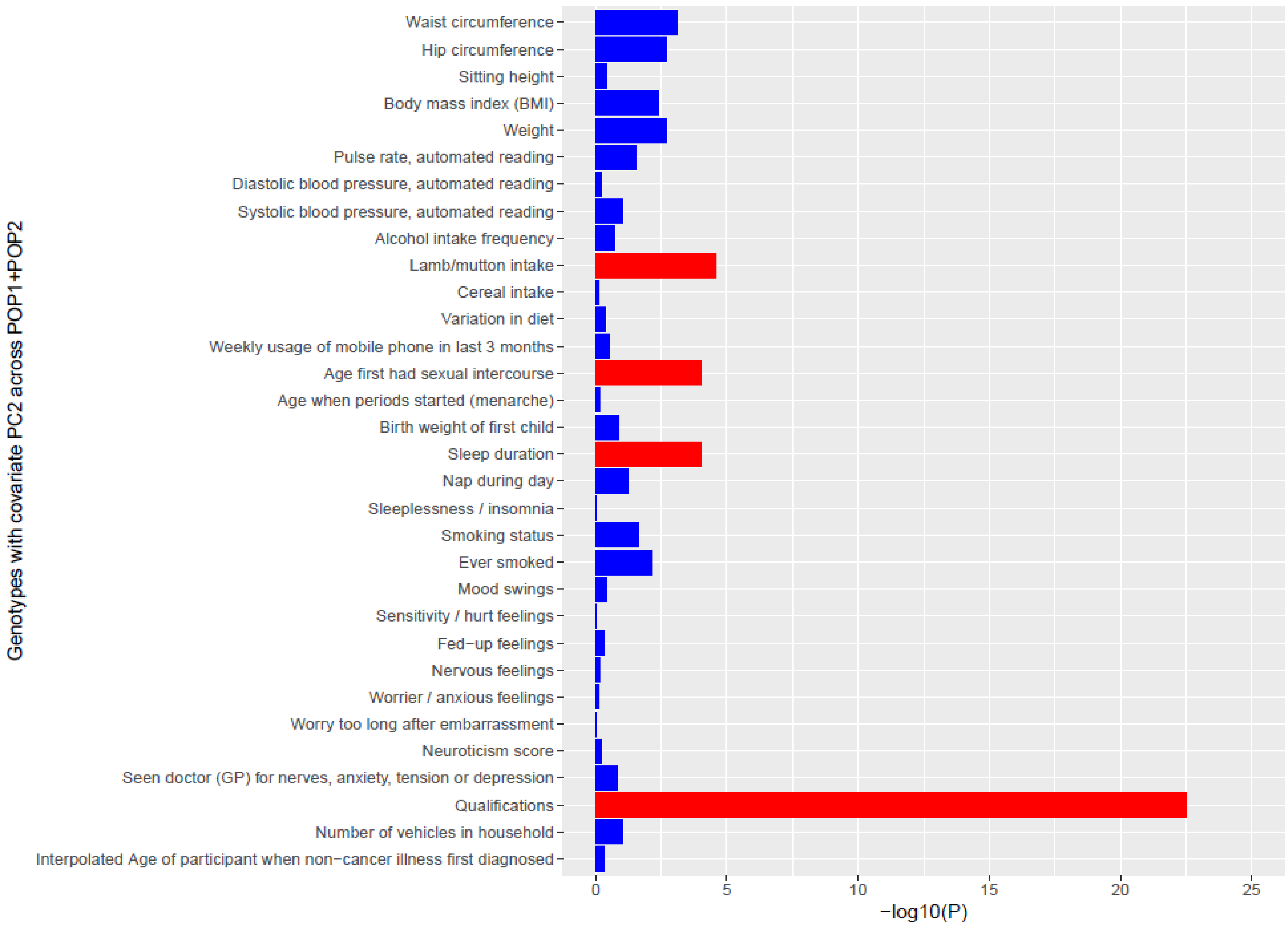
Genotypes with covariate PC2 across POP1+POP2
Waist circumference
Hip circumference
Sitting height
Body mass index (BMI)
Weight
Pulse rate, automated reading
Diastolic blood pressure, automated reading
Systolic blood pressure, automated reading
Alcohol intake frequency
Lamb/mutton intake
Cereal intake
Variation in diet
Weekly usage of mobile phone in last 3 months
Age first had sexual intercourse
Age when periods started (menarche)
Birth weight of first child
Sleep duration
Nap during day
Sleeplessness / insomnia
Smoking status
Ever smoked
Mood swings
Sensitivity / hurt feelings
Fed−up feelings
Nervous feelings
Worrier / anxious feelings
Worry too long after embarrassment
Neuroticism score
Seen doctor (GP) for nerves, anxiety, tension or depression
Qualifications
Number of vehicles in household
Interpolated Age of participant when non−cancer illness first diagnosed
0
5
10
15
20
25
−log10(P)

Figure S1. Detecting G×P interaction for (A) 29 traits with covariate PC1 and (B) 32 traits with covariate PC2. The phenotypic values for each trait were adjusted by basic confounders of fixed effects. Red colour indicates significant genetic heterogeneous signals (significance level was determined by Bonferroni multiple testing criterion: 0.05/140 = 3.57E-4).

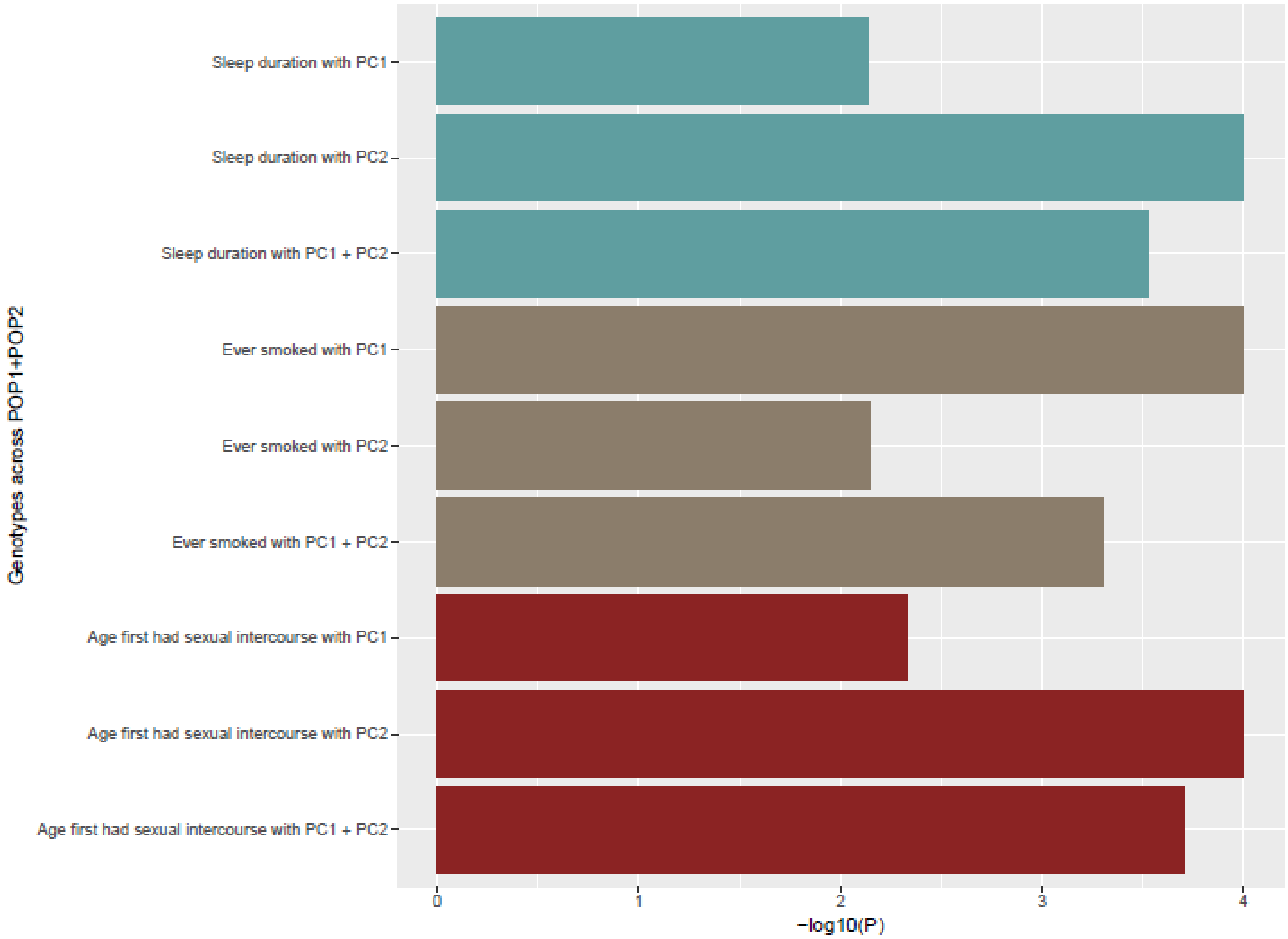

Figure S2. Comparisons of G×P interaction patterns using PC1, PC2 or simultaneous PC1 and PC2 for three valid traits in Table S11.

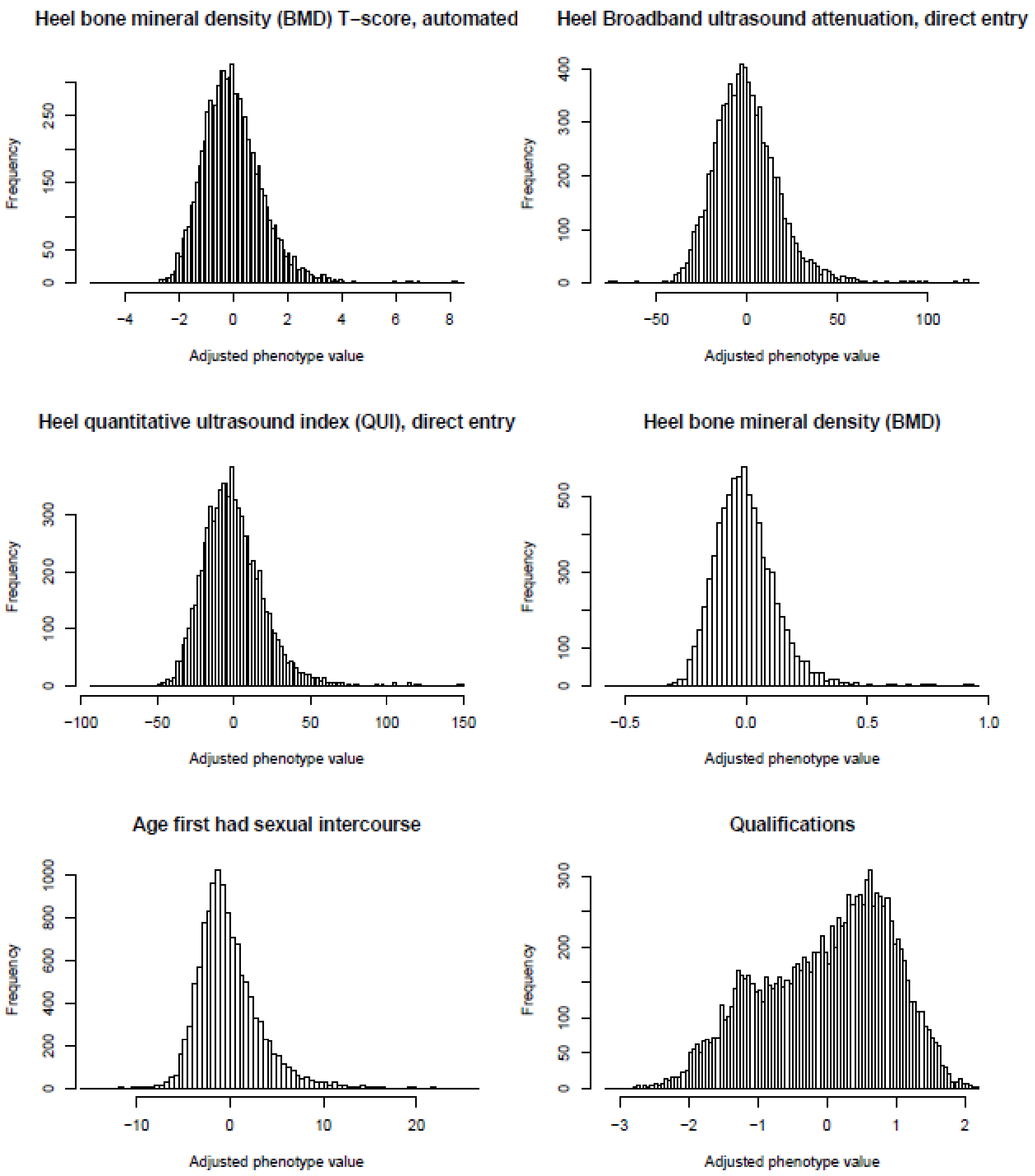

Figure S3. Distributions of phenotypic values after controlling basic and additional confounders of the six traits with significant G×P interactions.

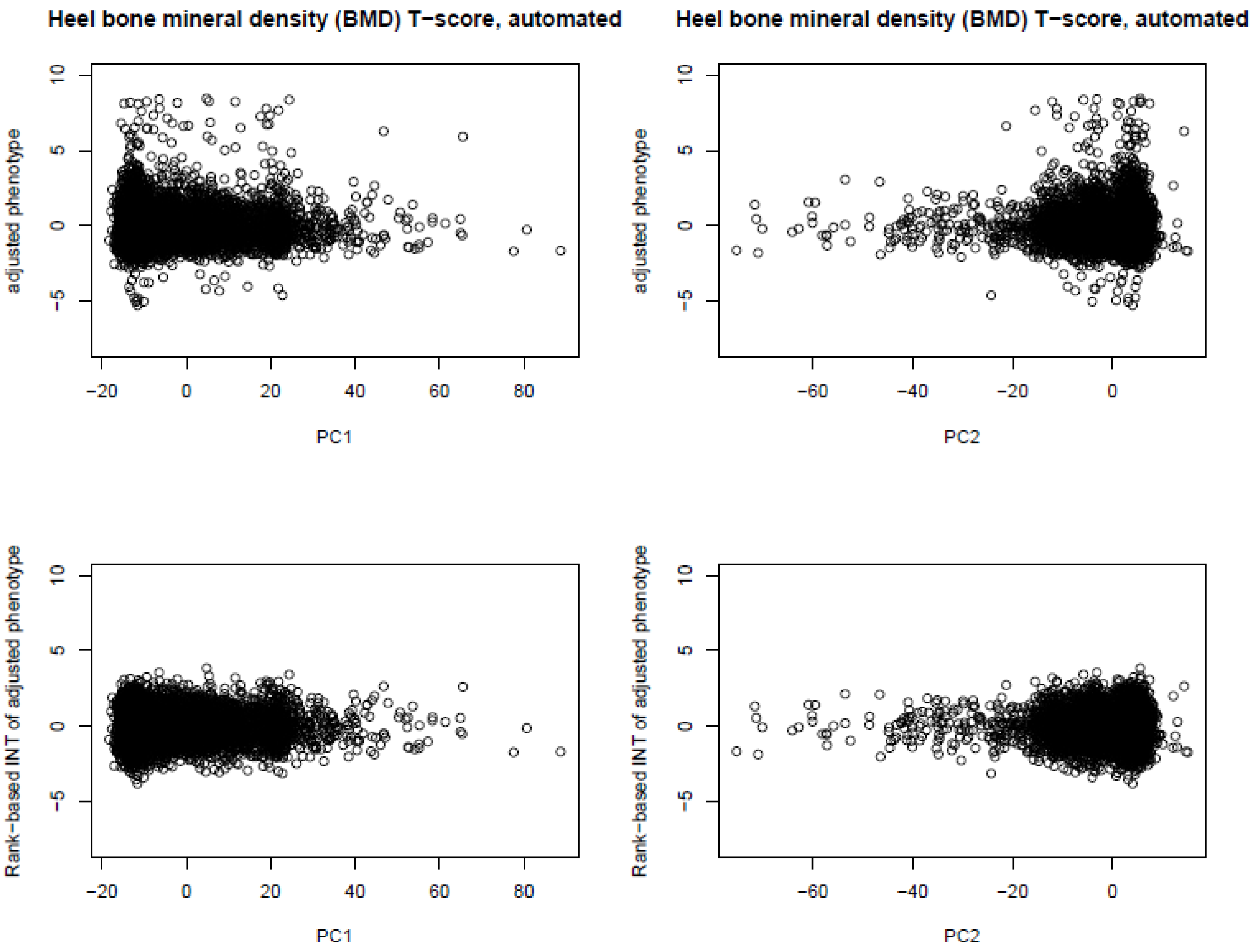

Figure S4. Phenotypic heteroscedasticity of the trait "heel bone mineral density (BMD) T-score, automated" before and after rank-based INT. The phenotypes were adjusted by basic and additional confounders of fixed effects.

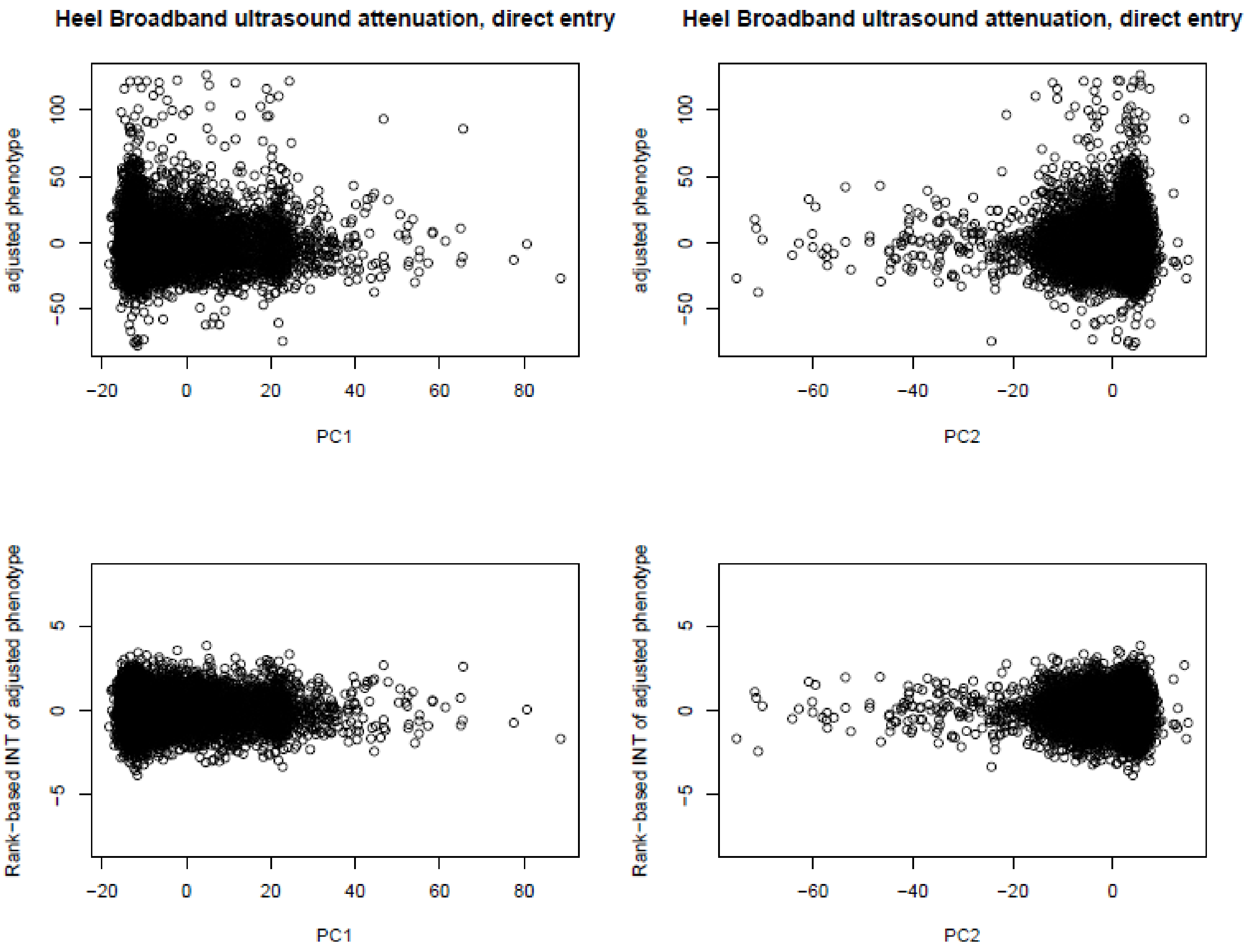

Figure S5. Phenotypic heteroscedasticity of the trait "heel broadband ultrasound attenuation, direct entry" before and after rank-based INT. The phenotypes were adjusted by basic and additional confounders of fixed effects.

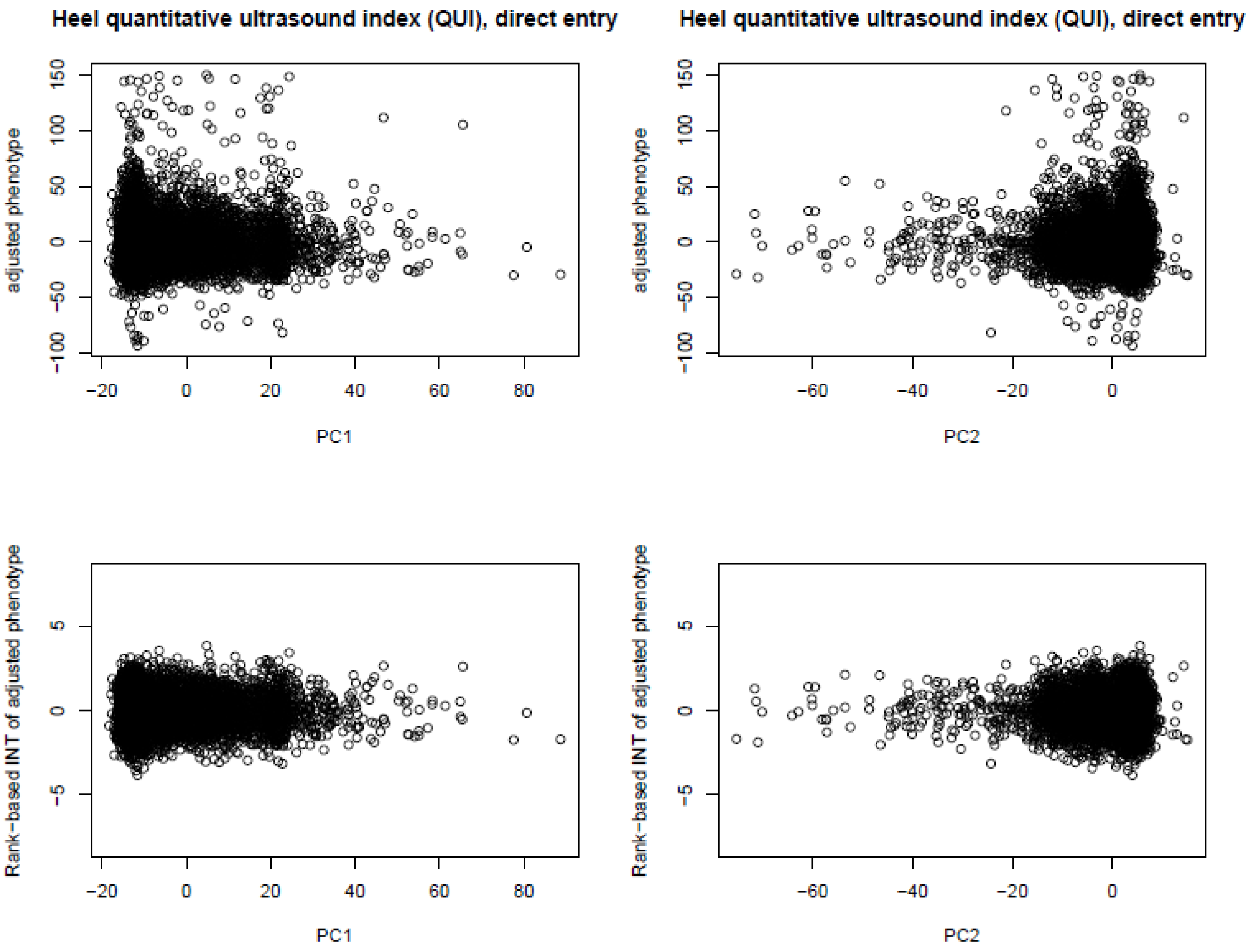

Figure S6. Phenotypic heteroscedasticity of the trait "heel QUI, direct entry" before and after rank-based INT. The phenotypes were adjusted by basic and additional confounders of fixed effects.

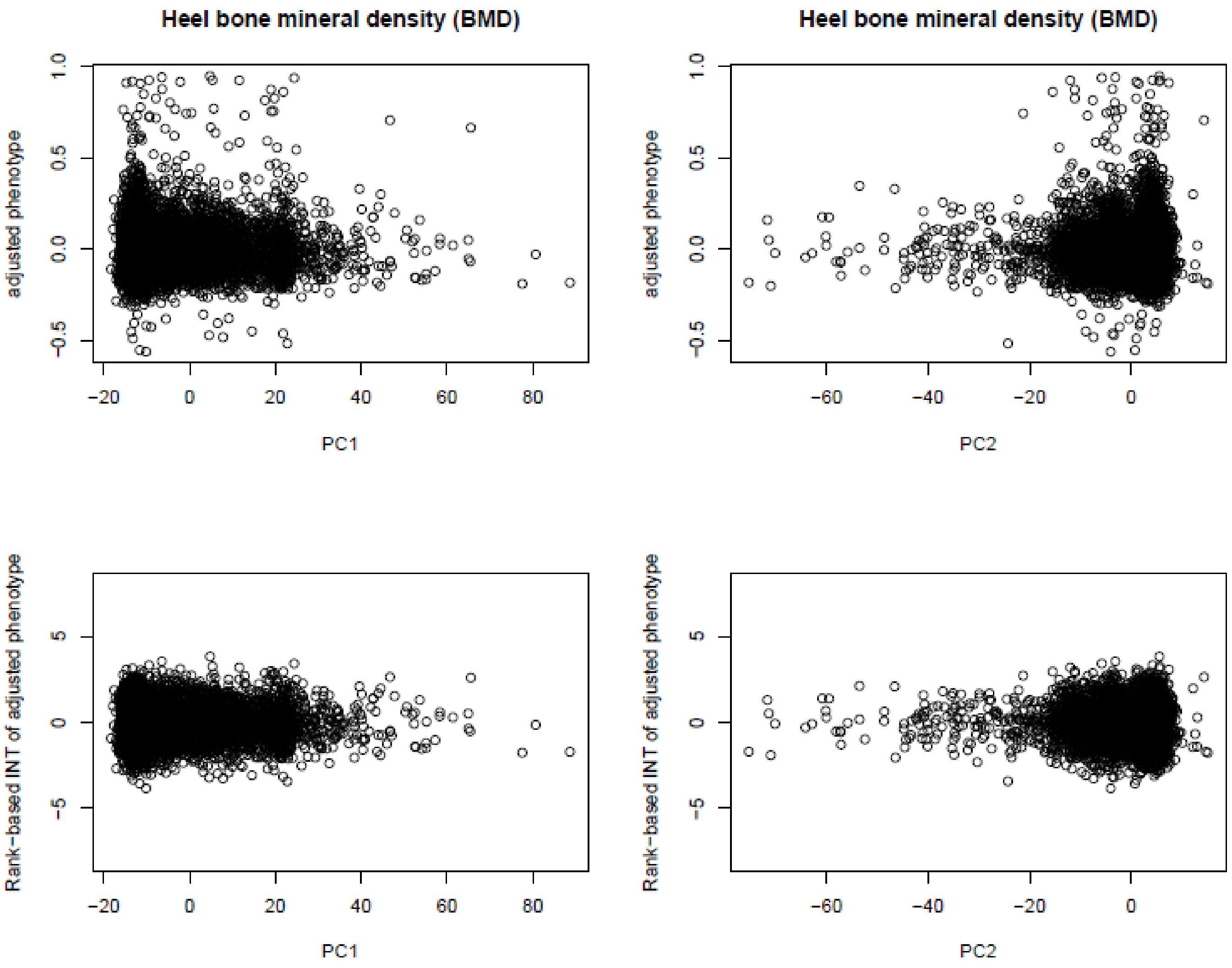

Figure S7. Phenotypic heteroscedasticity of the trait "heel BMD" before and after rank-based INT.

The phenotypes were adjusted by basic and additional confounders of fixed effects.

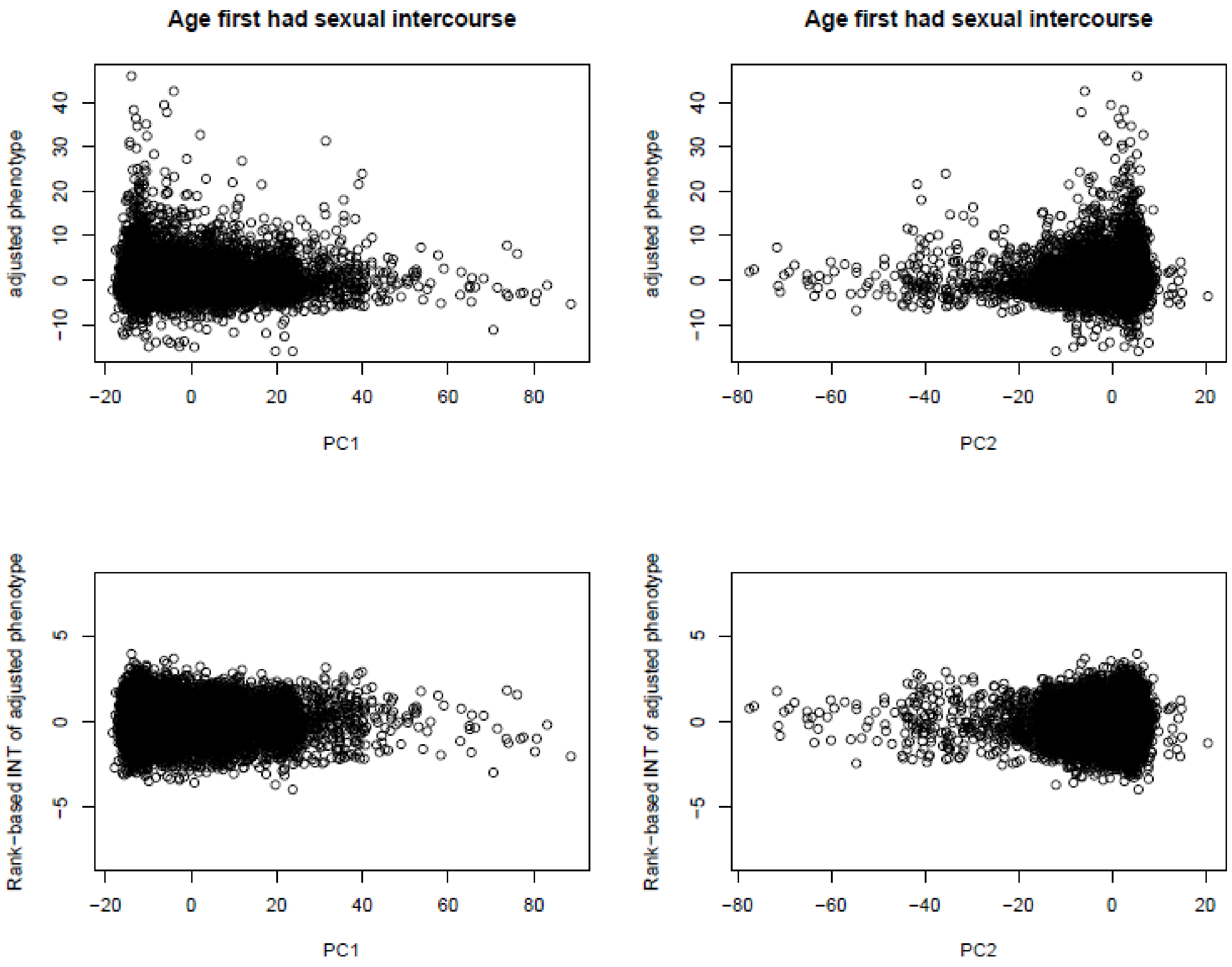

Figure S8. Phenotypic heteroscedasticity of the trait "age first had sexual intercourse" before and after rank-based INT. The phenotypes were adjusted by basic and additional confounders of fixed effects.

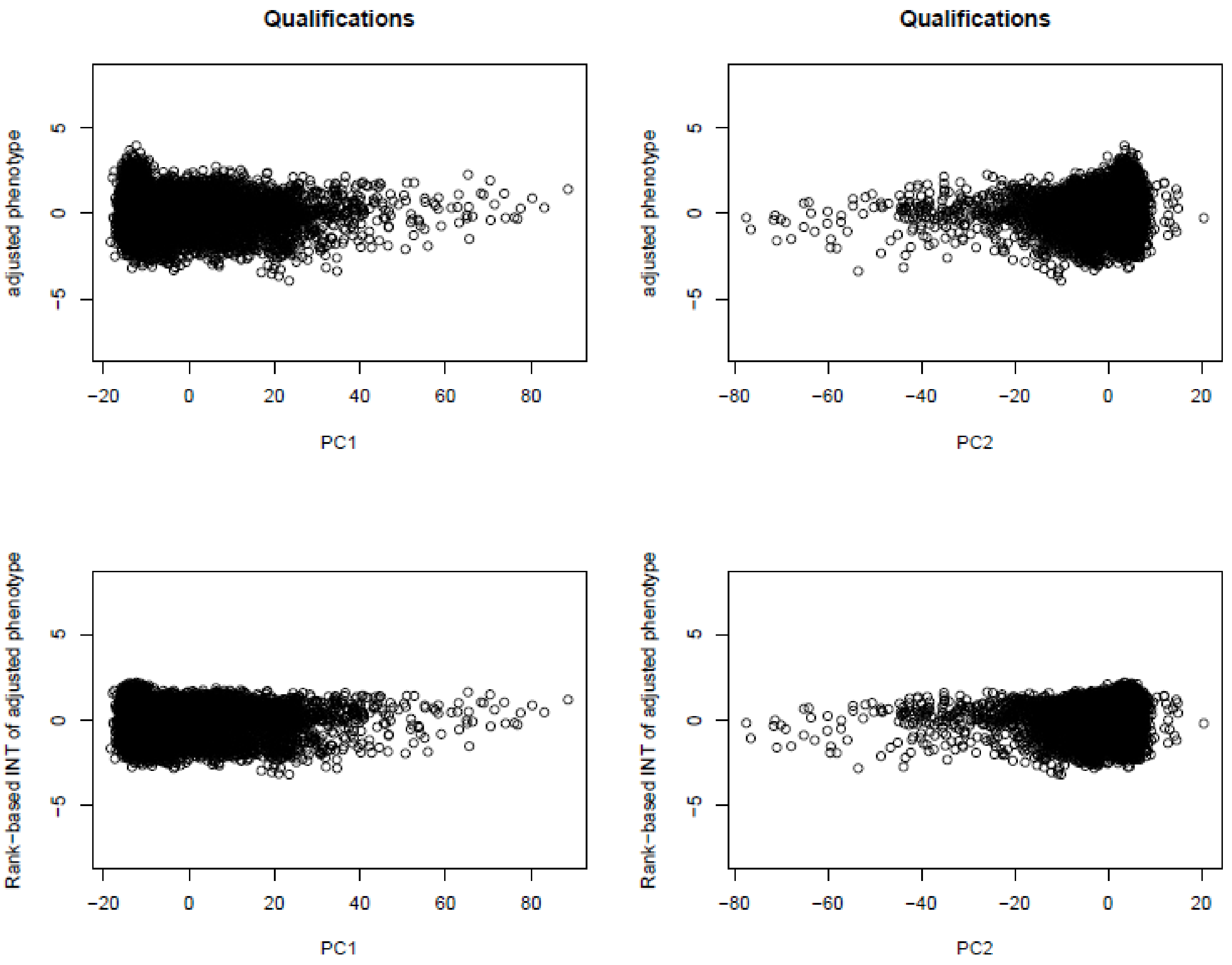

Figure S9. Phenotypic heteroscedasticity of the trait "qualifications" before and after rank-based INT.

The phenotypes were adjusted by basic and additional confounders of fixed effects.

Table S1. The sample sizes of 199 UKBB variables of POP1, POP2 and POP1+POP2. Since there exist numerous “Not Available” (NA) records for individuals in UKBB, the limited sample sizes of some variables may lead to insufficient statistical power to perform our study. Hence, the variables with limited sample size should be excluded. As POP2 and POP3 have the same ethnic background, we only examined sample sizes of POP1 and POP2, and used the following thresholds to exclude the variable with: non-NA number in POP1 < 2,500 and non-NA number in POP2 < 2,500, and then we remain 199 variables whose sample size in POP1+POP2 > 5,000 as shown in this table. Note that some ambiguous values in variables such as “Do not know” or “Prefer not to answer” were treated as NA.

UKBB data field	UKBB variable description	Variable value type	Non-NA No. in POP1	Non-NA No. in POP2	Non-NA No. in POP1+POP2
19	Heel ultrasound method	Unordered categorical	4997	4045	9042
31	Sex	Binary	7487	6913	14400
34	Year of birth	Continuous	7487	6913	14400
48	Waist circumference	Continuous	7485	6888	14373
49	Hip circumference	Continuous	7487	6890	14377
50	Standing height	Continuous	7487	6886	14373
52	Month of birth	Unordered categorical	7487	6913	14400
54	UK Biobank assessment centre	Unordered categorical	7487	6913	14400
78	Heel bone mineral density (BMD) T-score, automated	Continuous	4331	3442	7773
87	Non-cancer illness year/age first occurred	Continuous	5621	4875	10496
102	Pulse rate, automated reading	Continuous	6989	6608	13597
120	Birth weight known	Unordered categorical	7487	6909	14396
132	Job code	Unordered categorical	4869	5105	9974
134	Number of self-reported cancers	Continuous	7487	6909	14396
135	Number of self-reported non-cancer illnesses	Continuous	7487	6909	14396
189	Townsend deprivation index at recruitment	Continuous	7487	6906	14393
403	Number of times snap-button pressed	Continuous	7469	6850	14319
404	Duration to first press of snap-button in each round	Continuous	3116	2812	5928
670	Type of accommodation lived in	Unordered categorical	7487	6913	14400
680	Own or rent accommodation lived in	Unordered categorical	7469	6886	14355
699	Length of time at current address	Continuous	7487	6913	14400
709	Number in household	Continuous	7469	6886	14355
728	Number of vehicles in household	Ordered categorical	7469	6886	14355
738	Average total household income before tax	Ordered categorical	7469	6879	14348

757	Time employed in main current job	Continuous	4220	4635	8855
767	Length of working week for main job	Continuous	4220	4635	8855
777	Frequency of travelling from home to job workplace	Continuous	4220	4631	8851
796	Distance between home and job workplace	Continuous	3953	4196	8149
806	Job involves mainly walking or standing	Ordered categorical	4220	4635	8855
816	Job involves heavy manual or physical work	Ordered categorical	4220	4635	8855
826	Job involves shift work	Ordered categorical	4220	4635	8855
845	Age completed full time education	Continuous	5241	2953	8194
1110	Length of mobile phone use	Ordered categorical	7487	6913	14400
1120	Weekly usage of mobile phone in last 3 months	Ordered categorical	6363	6062	12425
1130	Hands-free device/speakerphone use with mobile phone in last 3 month	Ordered categorical	6363	6062	12425
1140	Difference in mobile phone use compared to two years previously	Ordered categorical	6363	6062	12425
1150	Usual side of head for mobile phone use	Binary	6363	6062	12425
1160	Sleep duration	Continuous	7487	6913	14400
1170	Getting up in morning	Ordered categorical	7487	6906	14393
1180	Morning/evening person (chronotype)	Ordered categorical	7487	6906	14393
1190	Nap during day	Ordered categorical	7487	6913	14400
1200	Sleeplessness / insomnia	Ordered categorical	7487	6913	14400
1210	Snoring	Binary	7487	6913	14400
1220	Daytime dozing / sleeping (narcolepsy)	Ordered categorical	7487	6913	14400
1239	Current tobacco smoking	Ordered categorical	7487	6913	14400
1249	Past tobacco smoking	Ordered categorical	6574	6219	12793
1259	Smoking/smokers in household	Unordered categorical	6574	6212	12786
1269	Exposure to tobacco smoke at home	Continuous	6574	6219	12793
1279	Exposure to tobacco smoke outside home	Continuous	6574	6219	12793
1289	Cooked vegetable intake	Continuous	7487	6913	14400
1299	Salad / raw vegetable intake	Continuous	7487	6913	14400
1309	Fresh fruit intake	Continuous	7487	6913	14400
1319	Dried fruit intake	Continuous	7487	6913	14400
1329	Oily fish intake	Ordered categorical	7487	6913	14400
1339	Non-oily fish intake	Ordered categorical	7487	6913	14400
1349	Processed meat intake	Ordered categorical	7487	6913	14400
1359	Poultry intake	Ordered categorical	7487	6913	14400

1369	Beef intake	Ordered categorical	7487	6913	14400
1379	Lamb/mutton intake	Ordered categorical	7487	6913	14400
1389	Pork intake	Ordered categorical	7487	6913	14400
1408	Cheese intake	Ordered categorical	7306	6694	14000
1418	Milk type used	Unordered categorical	7487	6913	14400
1428	Spread type	Unordered categorical	7487	6906	14393
1438	Bread intake	Continuous	7487	6906	14393
1448	Bread type	Unordered categorical	7259	6596	13855
1458	Cereal intake	Continuous	7487	6913	14400
1468	Cereal type	Unordered categorical	6147	4944	11091
1478	Salt added to food	Ordered categorical	7487	6913	14400
1488	Tea intake	Continuous	7487	6913	14400
1498	Coffee intake	Continuous	7487	6913	14400
1508	Coffee type	Unordered categorical	5850	5744	11594
1518	Hot drink temperature	Ordered categorical	7487	6913	14400
1528	Water intake	Continuous	7487	6913	14400
1538	Major dietary changes in the last 5 years	Unordered categorical	7487	6913	14400
1548	Variation in diet	Ordered categorical	7487	6906	14393
1558	Alcohol intake frequency.	Ordered categorical	7487	6913	14400
1568	Average weekly red wine intake	Continuous	5272	4205	9477
1578	Average weekly champagne plus white wine intake	Continuous	5272	4205	9477
1588	Average weekly beer plus cider intake	Continuous	5272	4205	9477
1598	Average weekly spirits intake	Continuous	5272	4205	9477
1608	Average weekly fortified wine intake	Continuous	5272	4205	9477
1618	Alcohol usually taken with meals	Ordered categorical	5868	5138	11006
1628	Alcohol intake versus 10 years previously	Ordered categorical	6952	6293	13245
1647	Country of birth (UK/elsewhere)	Unordered categorical	7487	6913	14400
1677	Breastfed as a baby	Binary	7487	6913	14400
1687	Comparative body size at age 10	Ordered categorical	7487	6913	14400
1697	Comparative height size at age 10	Ordered categorical	7487	6913	14400
1707	Handedness (chirality/laterality)	Binary	7487	6913	14400
1717	Skin colour	Ordered categorical	7487	6913	14400
1747	Hair colour (natural, before greying)	Unordered categorical	7487	6913	14400
1767	Adopted as a child	Binary	7487	6913	14400

1777	Part of a multiple birth	Binary	7374	6823	14197
1787	Maternal smoking around birth	Binary	7374	6823	14197
1920	Mood swings	Binary	7487	6913	14400
1930	Miserableness	Binary	7487	6913	14400
1940	Irritability	Binary	7487	6913	14400
1950	Sensitivity / hurt feelings	Binary	7487	6913	14400
1960	Fed-up feelings	Binary	7487	6913	14400
1970	Nervous feelings	Binary	7487	6913	14400
1980	Worrier / anxious feelings	Binary	7487	6913	14400
1990	Tense / 'highly strung'	Binary	7487	6913	14400
2000	Worry too long after embarrassment	Binary	7487	6913	14400
2010	Suffer from 'nerves'	Binary	7487	6913	14400
2020	Loneliness, isolation	Binary	7487	6913	14400
2030	Guilty feelings	Binary	7487	6913	14400
2040	Risk taking	Binary	7487	6913	14400
2050	Frequency of depressed mood in last 2 weeks	Ordered categorical	7487	6913	14400
2060	Frequency of unenthusiasm / disinterest in last 2 weeks	Ordered categorical	7487	6913	14400
2070	Frequency of tenseness / restlessness in last 2 weeks	Ordered categorical	7487	6913	14400
2080	Frequency of tiredness / lethargy in last 2 weeks	Ordered categorical	7487	6913	14400
2090	Seen doctor (GP) for nerves, anxiety, tension or depression	Binary	7487	6913	14400
2100	Seen a psychiatrist for nerves, anxiety, tension or depression	Binary	7487	6913	14400
2129	Answered sexual history questions	Binary	7487	6913	14400
2139	Age first had sexual intercourse	Continuous	6939	6173	13112
2149	Lifetime number of sexual partners	Continuous	6866	6130	12996
2159	Ever had same-sex intercourse	Binary	6866	6130	12996
2207	Wears glasses or contact lenses	Binary	7487	6913	14400
2217	Age started wearing glasses or contact lenses	Continuous	6692	5654	12346
2227	Other eye problems	Binary	7487	6913	14400
2237	Plays computer games	Ordered categorical	7487	6913	14400
2247	Hearing difficulty/problems	Binary	7487	6906	14393
2257	Hearing difficulty/problems with background noise	Binary	7484	6911	14395
2375	Relative age of first facial hair	Ordered categorical	3502	2768	6270
2385	Relative age voice broke	Ordered categorical	3502	2768	6270
2395	Hair/balding pattern	Ordered categorical	3502	2768	6270

2405	Number of children fathered	Continuous	3502	2768	6270
2443	Diabetes diagnosed by doctor	Binary	7487	6913	14400
2453	Cancer diagnosed by doctor	Binary	7487	6913	14400
2463	Fractured/broken bones in last 5 years	Binary	7487	6913	14400
2473	Other serious medical condition/disability diagnosed by doctor	Binary	7487	6913	14400
2492	Taking other prescription medications	Binary	7487	6913	14400
2654	Non-butter spread type details	Unordered categorical	3970	2509	6479
2664	Reason for reducing amount of alcohol drunk	Unordered categorical	3075	2543	5618
2714	Age when periods started (menarche)	Continuous	3985	4142	8127
2734	Number of live births	Continuous	3985	4142	8127
2744	Birth weight of first child	Continuous	3200	3080	6280
2784	Ever taken oral contraceptive pill	Binary	3985	4142	8127
3081	Foot measured for bone density	Binary	4964	3984	8948
3082	Fractured heel	Binary	4964	3984	8948
3140	Pregnant	Binary	3985	4131	8116
3143	Ankle spacing width	Continuous	4331	3442	7773
3144	Heel Broadband ultrasound attenuation, direct entry	Continuous	4331	3442	7773
3147	Heel quantitative ultrasound index (QUI), direct entry	Continuous	4331	3442	7773
3148	Heel bone mineral density (BMD)	Continuous	4327	3442	7769
3393	Hearing aid user	Binary	4455	4457	8912
4079	Diastolic blood pressure, automated reading	Continuous	6989	6608	13597
4080	Systolic blood pressure, automated reading	Continuous	6988	6608	13596
6138 **§**	Qualifications	Ordered categorical	7487	6906	14393
6141	How are people in household related to participant	Unordered categorical	6057	5485	11542
6142	Current employment status	Unordered categorical	7487	6913	14400
6143	Transport type for commuting to job workplace	Unordered categorical	3953	4196	8149
6144	Never eat eggs, dairy, wheat, sugar	Unordered categorical	7487	6906	14393
6145	Illness, injury, bereavement, stress in last 2 years	Unordered categorical	7487	6906	14393
6150	Vascular/heart problems diagnosed by doctor	Unordered categorical	7487	6913	14400
6152	Blood clot, DVT, bronchitis, emphysema, asthma, rhinitis, eczema, allergy diagnosed by doctor	Unordered categorical	7487	6913	14400
6153	Medication for cholesterol, blood pressure, diabetes, or take exogenous hormones	Unordered categorical	3985	4138	8123
6154	Medication for pain relief, constipation, heartburn	Unordered categorical	7487	6906	14393

6155	Vitamin and mineral supplements	Unordered categorical	7487	6906	14393
6177	Medication for cholesterol, blood pressure or diabetes	Unordered categorical	3502	2768	6270
6179	Mineral and other dietary supplements	Unordered categorical	7487	6906	14393
20002	Non-cancer illness code, self-reported	Unordered categorical	5621	4875	10496
20008	Interpolated Year when non-cancer illness first diagnosed	Continuous	5621	4875	10496
20009	Interpolated Age of participant when non-cancer illness first diagnosed	Continuous	5621	4875	10496
20013	Method of recording time when non-cancer illness first diagnosed	Unordered categorical	5621	4875	10496
20015	Sitting height	Continuous	7486	6881	14367
20022	Birth weight	Continuous	4273	3590	7863
20023	Mean time to correctly identify matches	Continuous	7440	6776	14216
20116	Smoking status	Ordered categorical	7487	6913	14400
20117	Alcohol drinker status	Ordered categorical	7487	6913	14400
20127	Neuroticism score	Continuous	6140	5065	11205
20160	Ever smoked	Binary	7487	6281	13768
21000	Ethnic background	Unordered categorical	7487	6913	14400
21001	Body mass index (BMI)	Continuous	7487	6878	14365
21002	Weight	Continuous	7487	6879	14366
21003	Age when attended assessment centre	Continuous	7487	6913	14400
21022	Age at recruitment	Continuous	7487	6913	14400
22000	Genotype measurement batch	Unordered categorical	7487	6913	14400
24003	Nitrogen dioxide air pollution; 2010	Continuous	7379	6677	14056
24004	Nitrogen oxides air pollution; 2010	Continuous	7379	6677	14056
24005	Particulate matter air pollution (pm10); 2010	Continuous	6801	6392	13193
24006	Particulate matter air pollution (pm2.5); 2010	Continuous	6801	6392	13193
24007	Particulate matter air pollution (pm2.5) absorbance; 2010	Continuous	6801	6392	13193
24008	Particulate matter air pollution 2.5-10um; 2010	Continuous	6801	6392	13193
24009	Traffic intensity on the nearest road	Continuous	7379	6677	14056
24010	Inverse distance to the nearest road	Continuous	7379	6677	14056
24011	Traffic intensity on the nearest major road	Continuous	7379	6677	14056
24012	Inverse distance to the nearest major road	Continuous	7379	6677	14056
24013	Total traffic load on major roads	Continuous	7379	6677	14056
24014	Close to major road	Binary	7379	6677	14056
24015	Sum of road length of major roads within 100m	Continuous	7379	6677	14056

24016	Nitrogen dioxide air pollution; 2005	Continuous	7379	6677	14056
24017	Nitrogen dioxide air pollution; 2006	Continuous	7379	6677	14056
24018	Nitrogen dioxide air pollution; 2007	Continuous	7379	6677	14056
24019	Particulate matter air pollution (pm10); 2007	Continuous	7356	6674	14030
24020	Average daytime sound level of noise pollution	Continuous	7379	6677	14056
24021	Average evening sound level of noise pollution	Continuous	7379	6677	14056
24022	Average night-time sound level of noise pollution	Continuous	7379	6677	14056
24023	Average 16-hour sound level of noise pollution	Continuous	7379	6677	14056
24024	Average 24-hour sound level of noise pollution	Continuous	7379	6677	14056

§: Qualifications (data field 6138) was reclassified and ordered as 1) none, 2) O-levels or CSEs, 3) A-levels, NVQ, HND, HNC or other professional qualification, and 4) college or university degree.

Table S2. The use of 199 UKBB variables for current study. 128 variables were selected as main phenotypes, which can be classified into a number of criteria, 1) lifestyle and environment (alcohol, diet, electronic device use, sexual factors, sleep, smoking and sun exposure), 2) physical measures (anthropometry, blood pressure and bone-densitometry of heel), 3) early life factors, 4) sociodemographics (education, employment and household), 5) health and medical history (eyesight, hearing, medical conditions and medication),6) psychosocial factors (mental health), 7) female-specific and male-specific factors, 8) verbal interview (medical conditions) and 9) cognitive function (reaction time). The other variables can be used as basic confounders, additional confounders or excluded. Note that some phenotypes such as from sociodemographics (e.g., qualifications) can also be used as additional confounders for other phenotypes.

UKBB data field	UKBB variable description	UKBB category	Note
31	Sex	Baseline characteristics	Confounder (basic)
34	Year of birth	Baseline characteristics	Confounder (basic)
52	Month of birth	Baseline characteristics	Excluded
189	Townsend deprivation index at recruitment	Baseline characteristics	Confounder
21022	Age at recruitment	Baseline characteristics	Confounder (basic)
22000	Genotype measurement batch	Genomics - Genotyping process and sample QC	Confounder (basic)
54	UK Biobank assessment centre	Reception - Recruitment	Confounder (basic)
21003	Age when attended assessment centre	Reception - Recruitment	Confounder (basic), same as "21022"
48	Waist circumference	Physical measures - Anthropometry	**Phenotype**
49	Hip circumference	Physical measures - Anthropometry	**Phenotype**
50	Standing height	Physical measures - Anthropometry	**Phenotype**
20015	Sitting height	Physical measures - Anthropometry	**Phenotype**
21001	Body mass index (BMI)	Physical measures - Anthropometry	**Phenotype**
21002	Weight	Physical measures - Anthropometry	**Phenotype**
102	Pulse rate, automated reading	Physical measures - Blood pressure	**Phenotype**
4079	Diastolic blood pressure, automated reading	Physical measures - Blood pressure	**Phenotype**
4080	Systolic blood pressure, automated reading	Physical measures - Blood pressure	**Phenotype**
19	Heel ultrasound method	Physical measures - Bone-densitometry of heel	Confounder
78	Heel bone mineral density (BMD) T-score, automated	Physical measures - Bone-densitometry of heel	**Phenotype**
3081	Foot measured for bone density	Physical measures - Bone-densitometry of heel	Confounder
3082	Fractured heel	Physical measures - Bone-densitometry of heel	Confounder
3143	Ankle spacing width	Physical measures - Bone-densitometry of heel	**Phenotype**

3144	Heel Broadband ultrasound attenuation, direct entry	Physical measures - Bone-densitometry of heel	**Phenotype**
3147	Heel quantitative ultrasound index (QUI), direct entry	Physical measures - Bone-densitometry of heel	**Phenotype**
3148	Heel bone mineral density (BMD)	Physical measures - Bone-densitometry of heel	**Phenotype**
1558	Alcohol intake frequency.	Lifestyle and environment - Alcohol	**Phenotype**
1568	Average weekly red wine intake	Lifestyle and environment - Alcohol	**Phenotype**
1578	Average weekly champagne plus white wine intake	Lifestyle and environment - Alcohol	**Phenotype**
1588	Average weekly beer plus cider intake	Lifestyle and environment - Alcohol	**Phenotype**
1598	Average weekly spirits intake	Lifestyle and environment - Alcohol	**Phenotype**
1608	Average weekly fortified wine intake	Lifestyle and environment - Alcohol	**Phenotype**
1618	Alcohol usually taken with meals	Lifestyle and environment - Alcohol	**Phenotype**
1628	Alcohol intake versus 10 years previously	Lifestyle and environment - Alcohol	**Phenotype**
2664	Reason for reducing amount of alcohol drunk	Lifestyle and environment - Alcohol	Confounder
20117	Alcohol drinker status	Lifestyle and environment - Alcohol	**Phenotype**
6144	Never eat eggs, dairy, wheat, sugar	Lifestyle and environment - Diet	Confounder
1289	Cooked vegetable intake	Lifestyle and environment - Diet	**Phenotype**
1299	Salad / raw vegetable intake	Lifestyle and environment - Diet	**Phenotype**
1309	Fresh fruit intake	Lifestyle and environment - Diet	**Phenotype**
1319	Dried fruit intake	Lifestyle and environment - Diet	**Phenotype**
1329	Oily fish intake	Lifestyle and environment - Diet	**Phenotype**
1339	Non-oily fish intake	Lifestyle and environment - Diet	**Phenotype**
1349	Processed meat intake	Lifestyle and environment - Diet	**Phenotype**
1359	Poultry intake	Lifestyle and environment - Diet	**Phenotype**
1369	Beef intake	Lifestyle and environment - Diet	**Phenotype**
1379	Lamb/mutton intake	Lifestyle and environment - Diet	**Phenotype**
1389	Pork intake	Lifestyle and environment - Diet	**Phenotype**
1408	Cheese intake	Lifestyle and environment - Diet	**Phenotype**
1418	Milk type used	Lifestyle and environment - Diet	Confounder
1428	Spread type	Lifestyle and environment - Diet	Confounder
1438	Bread intake	Lifestyle and environment - Diet	**Phenotype**
1448	Bread type	Lifestyle and environment - Diet	Confounder
1458	Cereal intake	Lifestyle and environment - Diet	**Phenotype**
1468	Cereal type	Lifestyle and environment - Diet	Confounder
1478	Salt added to food	Lifestyle and environment - Diet	**Phenotype**
1488	Tea intake	Lifestyle and environment - Diet	**Phenotype**

1498	Coffee intake	Lifestyle and environment - Diet	**Phenotype**
1508	Coffee type	Lifestyle and environment - Diet	Confounder
1518	Hot drink temperature	Lifestyle and environment - Diet	**Phenotype**
1528	Water intake	Lifestyle and environment - Diet	**Phenotype**
1538	Major dietary changes in the last 5 years	Lifestyle and environment - Diet	Confounder
1548	Variation in diet	Lifestyle and environment - Diet	**Phenotype**
2654	Non-butter spread type details	Lifestyle and environment - Diet	Confounder
1110	Length of mobile phone use	Lifestyle and environment - Electronic device use	**Phenotype**
1120	Weekly usage of mobile phone in last 3 months	Lifestyle and environment - Electronic device use	**Phenotype**
1130	Hands-free device/speakerphone use with mobile phone in last 3 month	Lifestyle and environment - Electronic device use	**Phenotype**
1140	Difference in mobile phone use compared to two years previously	Lifestyle and environment - Electronic device use	**Phenotype**
1150	Usual side of head for mobile phone use	Lifestyle and environment - Electronic device use	**Phenotype**
2237	Plays computer games	Lifestyle and environment - Electronic device use	**Phenotype**
2129	Answered sexual history questions	Lifestyle and environment - Sexual factors	Confounder
2139	Age first had sexual intercourse	Lifestyle and environment - Sexual factors	**Phenotype**
2149	Lifetime number of sexual partners	Lifestyle and environment - Sexual factors	**Phenotype**
2159	Ever had same-sex intercourse	Lifestyle and environment - Sexual factors	**Phenotype**
1160	Sleep duration	Lifestyle and environment - Sleep	**Phenotype**
1170	Getting up in morning	Lifestyle and environment - Sleep	**Phenotype**
1180	Morning/evening person (chronotype)	Lifestyle and environment - Sleep	**Phenotype**
1190	Nap during day	Lifestyle and environment - Sleep	**Phenotype**
1200	Sleeplessness / insomnia	Lifestyle and environment - Sleep	**Phenotype**
1210	Snoring	Lifestyle and environment - Sleep	**Phenotype**
1220	Daytime dozing / sleeping (narcolepsy)	Lifestyle and environment - Sleep	**Phenotype**
1239	Current tobacco smoking	Lifestyle and environment - Smoking	**Phenotype**
1249	Past tobacco smoking	Lifestyle and environment - Smoking	**Phenotype**
1259	Smoking/smokers in household	Lifestyle and environment - Smoking	Confounder
1269	Exposure to tobacco smoke at home	Lifestyle and environment - Smoking	Confounder
1279	Exposure to tobacco smoke outside home	Lifestyle and environment - Smoking	Confounder
20116	Smoking status	Lifestyle and environment - Smoking	**Phenotype**
20160	Ever smoked	Lifestyle and environment - Smoking	**Phenotype**
1717	Skin colour	Lifestyle and environment - Sun exposure	**Phenotype**

1747	Hair colour (natural, before greying)	Lifestyle and environment - Sun exposure	Confounder
1647	Country of birth (UK/elsewhere)	Early life factors	Confounder
1677	Breastfed as a baby	Early life factors	**Phenotype**
1687	Comparative body size at age 10	Early life factors	**Phenotype**
1697	Comparative height size at age 10	Early life factors	**Phenotype**
1707	Handedness (chirality/laterality)	Early life factors	**Phenotype**
1767	Adopted as a child	Early life factors	**Phenotype**
1777	Part of a multiple birth	Early life factors	**Phenotype**
1787	Maternal smoking around birth	Early life factors	**Phenotype**
20022	Birth weight	Early life factors	**Phenotype**
120	Birth weight known	Early life factors	Confounder
845	Age completed full time education	Sociodemographics - Education	**Phenotype**
6138	Qualifications	Sociodemographics - Education	**Phenotype** (reorganized)
132	Job code	Sociodemographics - Employment	Confounder
757	Time employed in main current job	Sociodemographics - Employment	**Phenotype**
767	Length of working week for main job	Sociodemographics - Employment	**Phenotype**
777	Frequency of travelling from home to job workplace	Sociodemographics - Employment	**Phenotype**
796	Distance between home and job workplace	Sociodemographics - Employment	**Phenotype**
806	Job involves mainly walking or standing	Sociodemographics - Employment	**Phenotype**
816	Job involves heavy manual or physical work	Sociodemographics - Employment	**Phenotype**
826	Job involves shift work	Sociodemographics - Employment	**Phenotype**
6142	Current employment status	Sociodemographics - Employment	Confounder
6143	Transport type for commuting to job workplace	Sociodemographics - Employment	Confounder
21000	Ethnic background	Sociodemographics - Ethnicity	Have been used for sampling
670	Type of accommodation lived in	Sociodemographics - Household	Confounder
680	Own or rent accommodation lived in	Sociodemographics - Household	Confounder
699	Length of time at current address	Sociodemographics - Household	**Phenotype**
709	Number in household	Sociodemographics - Household	**Phenotype**
728	Number of vehicles in household	Sociodemographics - Household	**Phenotype**
738	Average total household income before tax	Sociodemographics - Household	**Phenotype**
6141	How are people in household related to participant	Sociodemographics - Household	Confounder
2714	Age when periods started (menarche)	Female-specific factors	**Phenotype**
2734	Number of live births	Female-specific factors	**Phenotype**
2744	Birth weight of first child	Female-specific factors	**Phenotype**

2784	Ever taken oral contraceptive pill	Female-specific factors	Confounder
2375	Relative age of first facial hair	Male-specific factors	**Phenotype**
2385	Relative age voice broke	Male-specific factors	**Phenotype**
2395	Hair/balding pattern	Male-specific factors	**Phenotype**
2405	Number of children fathered	Male-specific factors	**Phenotype**
2207	Wears glasses or contact lenses	Health and medical history - Eyesight	**Phenotype**
2217	Age started wearing glasses or contact lenses	Health and medical history - Eyesight	**Phenotype**
2227	Other eye problems	Health and medical history - Eyesight	**Phenotype**
2247	Hearing difficulty/problems	Health and medical history - Hearing	**Phenotype**
2257	Hearing difficulty/problems with background noise	Health and medical history - Hearing	**Phenotype**
3393	Hearing aid user	Health and medical history - Hearing	**Phenotype**
2443	Diabetes diagnosed by doctor	Health and medical history - Medical conditions	**Phenotype**
2453	Cancer diagnosed by doctor	Health and medical history - Medical conditions	**Phenotype**
2463	Fractured/broken bones in last 5 years	Health and medical history - Medical conditions	**Phenotype**
2473	Other serious medical condition/disability diagnosed by doctor	Health and medical history - Medical conditions	**Phenotype**
6150	Vascular/heart problems diagnosed by doctor	Health and medical history - Medical conditions	Confounder
6152	Blood clot, DVT, bronchitis, emphysema, asthma, rhinitis, eczema, allergy diagnosed by doctor	Health and medical history - Medical conditions	Confounder
6153	Medication for cholesterol, blood pressure, diabetes, or take exogenous hormones	Health and medical history - Medical conditions	Confounder
2492	Taking other prescription medications	Health and medical history - Medication	Confounder
6155	Vitamin and mineral supplements	Health and medical history - Medication	Confounder
6177	Medication for cholesterol, blood pressure or diabetes	Health and medical history - Medication	Confounder
6179	Mineral and other dietary supplements	Health and medical history - Medication	Confounder
6154	Medication for pain relief, constipation, heartburn	Health and medical history - Medication	Confounder
403	Number of times snap-button pressed	Cognitive function - Reaction time	**Phenotype**
404	Duration to first press of snap-button in each round	Cognitive function - Reaction time	**Phenotype**
20023	Mean time to correctly identify matches	Cognitive function - Reaction time	**Phenotype**
1920	Mood swings	Psychosocial factors - Mental health	**Phenotype**
1930	Miserableness	Psychosocial factors - Mental health	**Phenotype**
1940	Irritability	Psychosocial factors - Mental health	**Phenotype**
1950	Sensitivity / hurt feelings	Psychosocial factors - Mental health	**Phenotype**
1960	Fed-up feelings	Psychosocial factors - Mental health	**Phenotype**

1970	Nervous feelings	Psychosocial factors - Mental health	**Phenotype**
1980	Worrier / anxious feelings	Psychosocial factors - Mental health	**Phenotype**
1990	Tense / 'highly strung'	Psychosocial factors - Mental health	**Phenotype**
2000	Worry too long after embarrassment	Psychosocial factors - Mental health	**Phenotype**
2010	Suffer from 'nerves'	Psychosocial factors - Mental health	**Phenotype**
2020	Loneliness, isolation	Psychosocial factors - Mental health	**Phenotype**
2030	Guilty feelings	Psychosocial factors - Mental health	**Phenotype**
2040	Risk taking	Psychosocial factors - Mental health	**Phenotype**
2050	Frequency of depressed mood in last 2 weeks	Psychosocial factors - Mental health	**Phenotype**
2060	Frequency of unenthusiasm / disinterest in last 2 weeks	Psychosocial factors - Mental health	**Phenotype**
2070	Frequency of tenseness / restlessness in last 2 weeks	Psychosocial factors - Mental health	**Phenotype**
2080	Frequency of tiredness / lethargy in last 2 weeks	Psychosocial factors - Mental health	**Phenotype**
2090	Seen doctor (GP) for nerves, anxiety, tension or depression	Psychosocial factors - Mental health	**Phenotype**
2100	Seen a psychiatrist for nerves, anxiety, tension or depression	Psychosocial factors - Mental health	**Phenotype**
6145	Illness, injury, bereavement, stress in last 2 years	Psychosocial factors - Mental health	Confounder
20127	Neuroticism score	Psychosocial factors - Mental health	**Phenotype**
87	Non-cancer illness year/age first occurred	Verbal interview - Medical conditions	Excluded
134	Number of self-reported cancers	Verbal interview - Medical conditions	**Phenotype**
135	Number of self-reported non-cancer illnesses	Verbal interview - Medical conditions	**Phenotype**
3140	Pregnant	Verbal interview - Medical conditions	Confounder
20002	Non-cancer illness code, self-reported	Verbal interview - Medical conditions	Confounder
20008	Interpolated Year when non-cancer illness first diagnosed	Verbal interview - Medical conditions	Confounder
20009	Interpolated Age of participant when non-cancer illness first diagnosed	Verbal interview - Medical conditions	**Phenotype**
20013	Method of recording time when non-cancer illness first diagnosed	Verbal interview - Medical conditions	Confounder
24003	Nitrogen dioxide air pollution; 2010	Residential air pollution	Confounder
24004	Nitrogen oxides air pollution; 2010	Residential air pollution	Confounder
24005	Particulate matter air pollution (pm10); 2010	Residential air pollution	Confounder
24006	Particulate matter air pollution (pm2.5); 2010	Residential air pollution	Confounder
24007	Particulate matter air pollution (pm2.5) absorbance; 2010	Residential air pollution	Confounder
24008	Particulate matter air pollution 2.5-10um; 2010	Residential air pollution	Confounder

24009	Traffic intensity on the nearest road	Residential air pollution	Confounder
24010	Inverse distance to the nearest road	Residential air pollution	Confounder
24011	Traffic intensity on the nearest major road	Residential air pollution	Confounder
24012	Inverse distance to the nearest major road	Residential air pollution	Confounder
24013	Total traffic load on major roads	Residential air pollution	Confounder
24014	Close to major road	Residential air pollution	Confounder
24015	Sum of road length of major roads within 100m	Residential air pollution	Confounder
24016	Nitrogen dioxide air pollution; 2005	Residential air pollution	Confounder
24017	Nitrogen dioxide air pollution; 2006	Residential air pollution	Confounder
24018	Nitrogen dioxide air pollution; 2007	Residential air pollution	Confounder
24019	Particulate matter air pollution (pm10); 2007	Residential air pollution	Confounder
24020	Average daytime sound level of noise pollution	Residential noise pollution	Confounder
24021	Average evening sound level of noise pollution	Residential noise pollution	Confounder
24022	Average night-time sound level of noise pollution	Residential noise pollution	Confounder
24023	Average 16-hour sound level of noise pollution	Residential noise pollution	Confounder
24024	Average 24-hour sound level of noise pollution	Residential noise pollution	Confounder

Table S3. Seventy UKBB phenotypes with significant SNP-based heritability estimated by GREML model across POP1+POP2. SE denotes standard error.

Phenotypes	UKBB data field	h^2_{SNP} (SE)
Physical measures - Anthropometry:		
Waist circumference	48	0.162(0.024)
Hip circumference	49	0.187(0.025)
Standing height	50	0.495(0.025)
Sitting height	20015	0.335(0.025)
Body mass index (BMI)	21001	0.204(0.025)
Weight	21002	0.216(0.025)
Physical measures - Blood pressure:		
Pulse rate, automated reading	102	0.155(0.025)
Diastolic blood pressure, automated reading	4079	0.122(0.025)
Systolic blood pressure, automated reading	4080	0.134(0.025)
Physical measures - Bone-densitometry of heel:		
Heel bone mineral density (BMD) T-score, automated	78	0.268(0.045)
Ankle spacing width	3143	0.176(0.044)
Heel Broadband ultrasound attenuation, direct entry	3144	0.233(0.044)
Heel quantitative ultrasound index (QUI), direct entry	3147	0.268(0.045)
Heel bone mineral density (BMD)	3148	0.256(0.045)
lifestyle and environment - Alcohol:		
Alcohol intake frequency	1558	0.067(0.023)
Average weekly spirits intake	1598	0.087(0.035)
Lifestyle and environment - Diet:		
Fresh fruit intake	1309	0.080(0.024)
Oily fish intake	1329	0.083(0.023)
Beef intake	1369	0.071(0.024)
Lamb/mutton intake	1379	0.050(0.023)
Cereal intake	1458	0.127(0.024)
Salt added to food	1478	0.097(0.024)
Tea intake	1488	0.054(0.023)
Hot drink temperature	1518	0.118(0.024)
Water intake	1528	0.121(0.024)
Variation in diet	1548	0.067(0.024)

Lifestyle and environment - Electronic device use:		
Weekly usage of mobile phone in last 3 months	1120	0.069(0.027)
Plays computer games	2237	0.098(0.024)
Lifestyle and environment - Sexual factors:		
Age first had sexual intercourse	2139	0.103(0.027)
Lifetime number of sexual partners	2149	0.170(0.031)
Ever had same-sex intercourse	2159	0.056(0.026)
Lifestyle and environment - Sleep:		
Sleep duration	1160	0.074(0.023)
Getting up in morning	1170	0.095(0.024)
Morning/evening person (chronotype)	1180	0.086(0.026)
Nap during day	1190	0.086(0.024)
Sleeplessness / insomnia	1200	0.103(0.024)
Lifestyle and environment - Smoking:		
Current tobacco smoking	1239	0.045(0.023)
Past tobacco smoking	1249	0.114(0.027)
Smoking status	20116	0.141(0.024)
Ever smoked	20160	0.133(0.025)
Lifestyle and environment - Sun exposure:		
Skin colour	1717	0.112(0.024)
Early life factors:		
Comparative body size at age 10	1687	0.126(0.025)
Comparative height size at age 10	1697	0.243(0.025)
Birth weight	20022	0.113(0.042)
Female-specific factors:		
Age when periods started (menarche)	2714	0.240(0.043)
Birth weight of first child	2744	0.158(0.055)
Male-specific factors:		
Relative age of first facial hair	2375	0.187(0.055)
Relative age voice broke	2385	0.165(0.058)
Hair/balding pattern	2395	0.347(0.055)
Psychosocial factors - Mental health:		
Mood swings	1920	0.081(0.024)
Irritability	1940	0.083(0.025)
Sensitivity / hurt feelings	1950	0.052(0.024)

Fed-up feelings	1960	0.069(0.024)
Nervous feelings	1970	0.066(0.024)
Worrier / anxious feelings	1980	0.104(0.024)
Tense / 'highly strung'	1990	0.069(0.025)
Worry too long after embarrassment	2000	0.134(0.025)
Neuroticism score	20127	0.170(0.031)
Guilty feelings	2030	0.100(0.024)
Risk taking	2040	0.094(0.025)
Frequency of depressed mood in last 2 weeks	2050	0.092(0.025)
Frequency of unenthusiasm / disinterest	2060	0.067(0.024)
Frequency of tenseness / restlessness	2070	0.094(0.025)
Frequency of tiredness / lethargy in last 2 weeks	2080	0.101(0.025)
Seen doctor (GP) for nerves, anxiety, tension or depression	2090	0.064(0.023)
Cognitive function - *Reaction time*:		
Number of times snap-button pressed	403	0.053(0.024)
Health and medical history - *Eyesight*:		
Age started wearing glasses or contact lenses	2217	0.098(0.028)
Sociodemographics - *Education*:		
Qualifications	6138	0.162(0.024)
Sociodemographics - *Household*:		
Number of vehicles in household	728	0.048(0.023)
Verbal interview - *Medical conditions*:		
Interpolated Age of participant when non-cancer illness first diagnosed	20009	0.094(0.034)

Table S4. **Fifty-eight UKBB phenotypes with significant SNP-based heritability estimated by GREML model across POP2+POP3**. SE denotes standard error.

Phenotypes	UKBB data field	h^2_{SNP} (SE)
Physical measures - *Anthropometry*:		
Waist circumference	48	0.205(0.024)
Hip circumference	49	0.201(0.024)
Standing height	50	0.493(0.024)
Sitting height	20015	0.365(0.024)
Body mass index (BMI)	21001	0.229(0.024)
Weight	21002	0.251(0.024)
Physical measures - *Blood pressure*:		
Pulse rate, automated reading	102	0.121(0.024)
Diastolic blood pressure, automated reading	4079	0.140(0.025)
Systolic blood pressure, automated reading	4080	0.130(0.025)
Physical measures - *Bone-densitometry of heel*:		
Heel bone mineral density (BMD) T-score, automated	78	0.207(0.045)
Ankle spacing width	3143	0.291(0.046)
Heel Broadband ultrasound attenuation, direct entry	3144	0.193(0.045)
Heel quantitative ultrasound index (QUI), direct entry	3147	0.207(0.045)
Heel bone mineral density (BMD)	3148	0.212(0.045)
lifestyle and environment - *Alcohol:*		
Alcohol intake frequency	1558	0.110(0.023)
Average weekly spirits intake	1598	0.076(0.035)
Alcohol intake versus 10 years previously	1628	0.063(0.025)
Lifestyle and environment - *Diet:*		
Fresh fruit intake	1309	0.066(0.023)
Dried fruit intake	1319	0.057(0.023)
Oily fish intake	1329	0.049(0.022)
Non-oily fish intake	1339	0.047(0.022)
Processed meat intake	1349	0.085(0.023)
Salt added to food	1478	0.098(0.023)
Hot drink temperature	1518	0.087(0.023)
Water intake	1528	0.105(0.023)

Variation in diet	1548	0.059(0.022)
Lifestyle and environment - Electronic device use:		
Length of mobile phone use	1110	0.074(0.023)
Hands-free device/speakerphone use with mobile phone in last 3 month	1130	0.053(0.027)
Plays computer games	2237	0.048(0.023)
Lifestyle and environment - Sexual factors:		
Age first had sexual intercourse	2139	0.074(0.025)
Lifestyle and environment - Sleep:		
Sleep duration	1160	0.081(0.023)
Getting up in morning	1170	0.120(0.023)
Morning/evening person (chronotype)	1180	0.138(0.025)
Nap during day	1190	0.050(0.023)
Sleeplessness / insomnia	1200	0.048(0.023)
Lifestyle and environment - Smoking:		
Past tobacco smoking	1249	0.088(0.026)
Smoking status	20116	0.081(0.024)
Lifestyle and environment - Sun exposure:		
Skin colour	1717	0.163(0.024)
Early life factors:		
Comparative body size at age 10	1687	0.177(0.024)
Comparative height size at age 10	1697	0.259(0.025)
Female-specific factors:		
Age when periods started (menarche)	2714	0.198(0.039)
Number of live births	2734	0.119(0.037)
Birth weight of first child	2744	0.141(0.053)
Male-specific factors:		
Relative age of first facial hair	2375	0.197(0.061)
Hair/balding pattern	2395	0.426(0.059)
Psychosocial factors - Mental health:		
Irritability	1940	0.049(0.023)
Fed-up feelings	1960	0.060(0.023)
Worry too long after embarrassment	2000	0.048(0.024)
Neuroticism score	20127	0.057(0.029)
Frequency of tenseness / restlessness	2070	0.064(0.024)

Frequency of tiredness / lethargy in last 2 weeks	2080	0.048(0.023)
Cognitive function - Reaction time:		
Duration to first press of snap-button in each round	404	0.115(0.056)
Health and medical history:		
Age started wearing glasses or contact lenses	2217	0.177(0.029)
Hearing difficulty/problems	2247	0.067(0.024)
Diabetes diagnosed by doctor	2443	0.092(0.023)
Sociodemographics - Education:		
Qualifications	6138	0.162(0.024)
Sociodemographics - Household:		
Length of time at current address	699	0.059(0.023)
Average total household income before tax	738	0.057(0.026)

Table S5. **Seventy UKBB phenotypes with significant SNP-based heritability estimated by GREML model across POP1+POP3**. SE denotes standard error.

Phenotypes	UKBB data field	h^2_{SNP} (SE)
Physical measures - Anthropometry:		
Waist circumference	48	0.182(0.023)
Hip circumference	49	0.212(0.023)
Standing height	50	0.548(0.023)
Sitting height	20015	0.378(0.023)
Body mass index (BMI)	21001	0.215(0.023)
Weight	21002	0.240(0.023)
Physical measures - Blood pressure:		
Pulse rate, automated reading	102	0.148(0.024)
Diastolic blood pressure, automated reading	4079	0.141(0.024)
Systolic blood pressure, automated reading	4080	0.136(0.024)
Physical measures - Bone-densitometry of heel:		
Heel bone mineral density (BMD) T-score, automated	78	0.303(0.040)
Ankle spacing width	3143	0.232(0.040)
Heel Broadband ultrasound attenuation, direct entry	3144	0.307(0.040)
Heel quantitative ultrasound index (QUI), direct entry	3147	0.303(0.040)
Heel bone mineral density (BMD)	3148	0.308(0.041)
lifestyle and environment - Alcohol:		
Alcohol intake frequency	1558	0.093(0.022)
Lifestyle and environment - Diet:		
Fresh fruit intake	1309	0.048(0.021)
Dried fruit intake	1319	0.044(0.022)
Oily fish intake	1329	0.058(0.021)
Non-oily fish intake	1339	0.045(0.021)
Processed meat intake	1349	0.059(0.021)
Poultry intake	1359	0.054(0.021)
Beef intake	1369	0.046(0.022)
Lamb/mutton intake	1379	0.046(0.021)
Cheese intake	1408	0.048(0.022)
Cereal intake	1458	0.065(0.021)

Salt added to food	1478	0.084(0.022)
Hot drink temperature	1518	0.090(0.022)
Water intake	1528	0.061(0.022)
Lifestyle and environment - Electronic device use:		
Length of mobile phone use	1110	0.070(0.022)
Plays computer games	2237	0.069(0.022)
Lifestyle and environment - Sexual factors:		
Age first had sexual intercourse	2139	0.146(0.025)
Lifestyle and environment - Sleep:		
Sleep duration	1160	0.077(0.022)
Getting up in morning	1170	0.119(0.022)
Morning/evening person (chronotype)	1180	0.162(0.025)
Nap during day	1190	0.094(0.022)
Sleeplessness / insomnia	1200	0.098(0.022)
Snoring	1210	0.076(0.024)
Lifestyle and environment - Smoking:		
Current tobacco smoking	1239	0.055(0.022)
Past tobacco smoking	1249	0.092(0.025)
Smoking status	20116	0.116(0.022)
Ever smoked	20160	0.093(0.023)
Lifestyle and environment - Sun exposure:		
Skin colour	1717	0.180(0.023)
Early life factors:		
Comparative body size at age 10	1687	0.130(0.023)
Comparative height size at age 10	1697	0.279(0.023)
Birth weight	20022	0.118(0.038)
Female-specific factors:		
Age when periods started (menarche)	2714	0.183(0.038)
Number of live births	2734	0.099(0.038)
Male-specific factors:		
Relative age of first facial hair	2375	0.210(0.053)
Hair/balding pattern	2395	0.436(0.053)
Psychosocial factors - Mental health:		
Mood swings	1920	0.117(0.022)
Miserableness	1930	0.060(0.022)

Irritability<br>Sensitivity / hurt feelings<br>Fed-up feelings<br>Nervous feelings<br>Worrier / anxious feelings<br>Tense / 'highly strung'<br>Worry too long after embarrassment<br>Neuroticism score<br>Guilty feelings<br>Risk taking<br>Frequency of tiredness / lethargy in last 2 weeks<br>Seen doctor (GP) for nerves, anxiety, tension or depression	1940<br>1950<br>1960<br>1970<br>1980<br>1990<br>2000<br>20127<br>2030<br>2040<br>2080<br>2090	0.099(0.023)<br>0.055(0.023)<br>0.099(0.022)<br>0.056(0.022)<br>0.095(0.022)<br>0.113(0.023)<br>0.077(0.023)<br>0.108(0.027)<br>0.069(0.022)<br>0.068(0.023)<br>0.079(0.022)<br>0.046 (0.021)
Cognitive function - *Reaction time*:<br>Mean time to correctly identify matches	 20023	 0.071(0.022)
Health and medical history:<br>Age started wearing glasses or contact lenses<br>Diabetes diagnosed by doctor<br>Fractured/broken bones in last 5 years	 2217<br>2443<br>2463	 0.141(0.026)<br>0.049(0.021)<br>0.050(0.022)
Sociodemographics - *Education*:<br>Qualifications	 6138	 0.173(0.022)
Sociodemographics - *Household*:<br>Average total household income before tax	 738	 0.060(0.024)
Verbal interview - *Medical conditions*:<br>Number of self-reported non-cancer illnesses	 135	 0.078(0.022)

Table S6. **Genetic and residual covariances between 70 UKBB phenotypes and PC1/PC2 estimated by bivariate GREML model across POP1+POP2**. SE denotes standard error.

UKBB data field	Phenotypes	Genetic covariance with PC1 (SE)	Genetic covariance with PC2 (SE)	Residual covariance with PC1 (SE)	Residual covariance with PC2 (SE)
	Physical measures - Anthropometry:				
48	Waist circumference	0.0041(0.0189)	0.0007(0.017)	-0.0176(0.0178)	-0.0033(0.0167)
49	Hip circumference	0.0085(0.0194)	-0.0045(0.0174)	-0.0208(0.0180)	0.009(0.0168)
50	Standing height	-0.0015(0.0225)	-0.0031(0.0204)	0.0164(0.0187)	0.0164(0.0175)
20015	Sitting height	-0.0050(0.0213)	-0.0064(0.0192)	0.0188(0.0187)	0.0188(0.0174)
21001	Body mass index (BMI)	0.0073(0.0197)	0.0015(0.0177)	-0.0206(0.0181)	-0.0092(0.0169)
21002	Weight	0.0044(0.0199)	-0.003(0.0178)	-0.0028(0.0182)	0.005(0.017)
	Physical measures - Blood pressure:				
102	Pulse rate, automated reading	-0.0035(0.0191)	0.0073(0.0172)	0.0220(0.0181)	-0.0219(0.017)
4079	Diastolic blood pressure, automated reading	-0.0004(0.0184)	-0.0066(0.0166)	0.0002(0.0179)	0.0222(0.0168)
4080	Systolic blood pressure, automated reading	-0.0070(0.0187)	-0.005(0.0169)	0.0222(0.0180)	0.0133(0.0169)
	Physical measures - Bone-densitometry of heel:				
78	Heel bone mineral density (BMD) T-score, automated	0.0011(0.0259)	-0.0118(0.0234)	-0.0011(0.0244)	0.029(0.0229)
3143	Ankle spacing width	0.0044(0.0240)	-0.0043(0.0219)	-0.0048(0.0233)	0.0072(0.0221)
3144	Heel Broadband ultrasound attenuation, direct entry	0.0062(0.0252)	-0.0073(0.0229)	-0.0294(0.0241)	0.0212(0.0227)
3147	Heel quantitative ultrasound index (QUI), direct entry	0.0011(0.0259)	-0.0118(0.0234)	-0.0011(0.0244)	0.029(0.0229)
3148	Heel bone mineral density (BMD)	0.0017(0.0257)	-0.0123(0.0232)	-0.0034(0.0243)	0.0291(0.0228)
	lifestyle and environment - Alcohol:				
1558	Alcohol intake frequency	0.0028(0.0163)	0.0086(0.0149)	-0.0065(0.0165)	-0.0223(0.0157)
1598	Average weekly spirits intake	-0.0044(0.0203)	-0.0038(0.0188)	0.0175(0.0204)	0.0173(0.0196)
	Lifestyle and environment - Diet:				
1309	Fresh fruit intake	0.0012(0.0169)	0.0002(0.0154)	-0.0082(0.0168)	-0.0023(0.0159)
1329	Oily fish intake	-0.0044(0.0169)	0.0042(0.0154)	0.0180(0.0168)	-0.0151(0.0159)
1369	Beef intake	0.0059(0.0165)	0.0012(0.0151)	-0.0223(0.0166)	-0.0046(0.0158)
1379	Lamb/mutton intake	0.0042(0.0156)	0.0066(0.0145)	-0.0226(0.0161)	-0.0226(0.0155)
1458	Cereal intake	0.0023(0.0182)	-0.0024(0.0164)	-0.0017(0.0174)	0.0027(0.0164)
1478	Salt added to food	0.0047(0.0174)	-0.0021(0.0158)	-0.0219(0.0171)	0.0082(0.0162)
1488	Tea intake	-0.0012(0.0157)	0.0052(0.0145)	0.0012(0.0161)	-0.0198(0.0154)
1518	Hot drink temperature	-0.0076(0.0181)	-0.005(0.0163)	0.0218(0.0175)	0.0218(0.0164)

1528	Water intake	-0.0012(0.0181)	-0.0091(0.0163)	-0.0088(0.0175)	0.0217(0.0164)
1548	Variation in diet	-0.0085(0.0164)	-0.0057(0.0151)	0.0224(0.0167)	0.0224(0.0159)
	Lifestyle and environment - Electronic device use:				
1120	Weekly usage of mobile phone in last 3 months	0.0039(0.0172)	-0.0043(0.0159)	-0.0242(0.0176)	0.0189(0.0169)
2237	Plays computer games	0.0039(0.0174)	-0.0023(0.0157)	-0.0219(0.0170)	0.0112(0.0161)
	Lifestyle and environment - Sexual factors:				
2139	Age first had sexual intercourse	0.0039(0.0184)	0.0002(0.0168)	-0.0236(0.0181)	-0.0003(0.0172)
2149	Lifetime number of sexual partners	-0.0100(0.0209)	-0.0066(0.0189)	0.0241(0.0200)	0.024(0.0187)
2159	Ever had same-sex intercourse	-0.0064(0.0165)	-0.0019(0.0155)	0.0238(0.0171)	0.0065(0.0165)
	Lifestyle and environment - Sleep:				
1160	Sleep duration	-0.0058(0.0166)	-0.0066(0.0152)	0.0223(0.0166)	0.0222(0.0158)
1170	Getting up in morning	-0.0027(0.0174)	0.0003(0.0157)	0.0166(0.0171)	-0.0023(0.0161)
1180	Morning/evening person (chronotype)	-0.0043(0.0176)	0.0005(0.0161)	0.0234(0.0176)	-0.0003(0.0167)
1190	Nap during day	-0.0050(0.0171)	0.0096(0.0156)	0.0221(0.0170)	-0.0221(0.0161)
1200	Sleeplessness / insomnia	0.0001(0.0176)	0.0061(0.0159)	0.0002(0.0172)	-0.0219(0.0162)
	Lifestyle and environment - Smoking:				
1239	Current tobacco smoking	-0.0047(0.0153)	0.0005(0.0143)	0.0226(0.0159)	-0.0043(0.0154)
1249	Past tobacco smoking	0.0092(0.0186)	0.0068(0.0169)	-0.0232(0.0182)	-0.0221(0.0172)
20116	Smoking status	-0.0083(0.0185)	-0.0006(0.0166)	0.0214(0.0176)	-0.0024(0.0165)
20160	Ever smoked	-0.0076(0.0186)	-0.0052(0.0168)	0.0221(0.0178)	0.0165(0.0168)
	Lifestyle and environment - Sun exposure:				
1717	Skin colour	0.009(0.0179)	-0.0007(0.0162)	-0.0219(0.0174)	0.0041(0.0164)
	Early life factors:				
1687	Comparative body size at age 10	0.0048(0.0184)	-0.006(0.0166)	-0.0219(0.0177)	0.0219(0.0166)
1697	Comparative height size at age 10	0.0043(0.0203)	0.0022(0.0182)	-0.0135(0.0184)	-0.0015(0.0171)
20022	Birth weight	0.0047(0.0221)	0.006(0.0206)	-0.0303(0.0223)	-0.0184(0.0214)
	Female-specific factors:				
2714	Age when periods started (menarche)	-0.0073(0.0247)	-0.0079(0.0222)	0.0291(0.0236)	0.0097(0.0221)
2744	Birth weight of first child	-0.0023(0.0256)	-0.0076(0.0236)	0.0059(0.0257)	0.0349(0.0245)
	Male-specific factors:				
2375	Relative age of first facial hair	0.0042(0.026)	-0.0115(0.0242)	-0.0331(0.026)	0.0345(0.0249)
2385	Relative age voice broke	-0.0022(0.0259)	0.004(0.0244)	0.0024(0.0263)	-0.0148(0.0254)
2395	Hair/balding pattern	-0.0032(0.0284)	0.0027(0.0258)	0.0014(0.0269)	0.0016(0.0254)
	Psychosocial factors - Mental health:				
1920	Mood swings	0.0011(0.0171)	0.001(0.0157)	-0.0083(0.0171)	-0.0057(0.0162)

1940	Irritability	-0.0065(0.0173)	0.0034(0.0159)	0.0227(0.0173)	-0.0115(0.0165)
1950	Sensitivity / hurt feelings	0.0046(0.0159)	0.0017(0.0148)	-0.0229(0.0164)	-0.0098(0.0158)
1960	Fed-up feelings	-0.0034(0.0167)	0.0045(0.0153)	0.0212(0.0169)	-0.0219(0.0161)
1970	Nervous feelings	-0.0038(0.0165)	0.004(0.0152)	0.0226(0.0167)	-0.0227(0.016)
1980	Worrier / anxious feelings	0.0007(0.0178)	0.0059(0.0161)	0.0001(0.0174)	-0.0223(0.0164)
1990	Tense / 'highly strung'	-0.002(0.0168)	0.0031(0.0155)	0.0141(0.017)	-0.0153(0.0163)
2000	Worry too long after embarrassment	0.0047(0.0187)	0.0022(0.0169)	-0.0221(0.0179)	-0.0111(0.0168)
20127	Neuroticism score	-0.0008(0.0208)	0.0048(0.019)	0.0176(0.0199)	-0.0244(0.0187)
2030	Guilty feelings	0.0074(0.0177)	0.0011(0.0161)	-0.0223(0.0173)	-0.0051(0.0164)
2040	Risk taking	-0.0018(0.0176)	0.0072(0.016)	0.0006(0.0174)	-0.0226(0.0164)
2050	Frequency of depressed mood in last 2 weeks	0.0012(0.0175)	-0.0076(0.016)	-0.0112(0.0173)	0.0226(0.0164)
2060	Frequency of unenthusiasm / disinterest	-0.0001(0.0166)	0.0009(0.0153)	-0.0004(0.0168)	-0.0052(0.0161)
2070	Frequency of tenseness / restlessness	0.0046(0.0176)	0.0073(0.0161)	-0.0227(0.0174)	-0.0226(0.0165)
2080	Frequency of tiredness / lethargy in last 2 weeks	-0.0051(0.0177)	0.0074(0.0161)	0.0223(0.0174)	-0.0223(0.0164)
2090	Seen doctor (GP) for nerves, anxiety, tension or depression	-0.0043(0.0163)	0.0005(0.015)	0.0111(0.0165)	0.0001(0.0158)
403	*Cognitive function* - *Reaction time*: Number of times snap-button pressed	-0.0032(0.0158)	-0.0003(0.0146)	0.0112(0.0164)	0.0057(0.0157)
2217	*Health and medical history* - *Eyesight*: Age started wearing glasses or contact lenses	-0.0053(0.0186)	-0.006(0.017)	0.0242(0.0184)	0.0211(0.0175)
6138	*Sociodemographics* - *Education*: Qualifications	-0.0063(0.0189)	-0.0096(0.017)	0.0213(0.0178)	0.0213(0.0166)
728	*Sociodemographics* - *Household*: Number of vehicles in household	-0.0045(0.0155)	-0.0012(0.0145)	0.0227(0.0162)	0.0057(0.0156)
20009	*Verbal interview* - *Medical conditions*: Interpolated Age of participant when non-cancer illness first diagnosed	0.0006(0.0194)	-0.0065(0.0179)	-0.0009(0.0197)	0.025(0.0188)

Table S7. Genetic and residual covariances between 58 UKBB phenotypes and PC1/PC2 estimated by bivariate GREML model across POP2+POP3. SE denotes standard error.

UKBB data field	Phenotypes	Genetic covariance with PC1 (SE)	Genetic covariance with PC2 (SE)	Residual covariance with PC1 (SE)	Residual covariance with PC2 (SE)
	Physical measures - Anthropometry:				
48	Waist circumference	0.0003(0.0192)	0.004(0.0171)	0.0015(0.0177)	-0.0083(0.0163)
49	Hip circumference	0.0038(0.0192)	0.0048(0.0181)	-0.0019(0.0178)	-0.0079(0.0165)
50	Standing height	0.0045(0.0218)	0.0003(0.0197)	0.0068(0.0181)	-0.0067(0.0169)
20015	Sitting height	0.0048(0.0209)	0.002(0.0187)	0.0076(0.0181)	-0.0074(0.0168)
21001	Body mass index (BMI)	0.0032(0.0195)	0.0056(0.0183)	-0.0082(0.0178)	-0.0078(0.0165)
21002	Weight	0.0016(0.0199)	0.0045(0.0185)	0.0082(0.018)	-0.0077(0.0166)
	Physical measures - Blood pressure:				
102	Pulse rate, automated reading	-0.0043(0.0178)	0.0045(0.016)	0.0091(0.0173)	-0.0091(0.0162)
4079	Diastolic blood pressure, automated reading	0.0009(0.0183)	-0.0005(0.0164)	-0.009(0.0175)	0.0087(0.0164)
4080	Systolic blood pressure, automated reading	-0.0027(0.018)	-0.0015(0.0162)	0.009(0.0174)	0.0072(0.0163)
	Physical measures - Bone-densitometry of heel:				
78	Heel bone mineral density (BMD) T-score, automated	0.0107(0.0241)	-0.0096(0.023)	-0.0124(0.0234)	0.0121(0.0224)
3143	Ankle spacing width	0.0015(0.0257)	0.0014(0.0231)	0.0024(0.0243)	-0.0121(0.0227)
3144	Heel Broadband ultrasound attenuation, direct entry	0.0092(0.0239)	-0.0086(0.0234)	-0.0124(0.0233)	0.012(0.0225)
3147	Heel quantitative ultrasound index (QUI), direct entry	0.0107(0.0241)	-0.0096(0.023)	-0.0124(0.0234)	0.0121(0.0224)
3148	Heel bone mineral density (BMD)	0.0032(0.0243)	-0.0098(0.0231)	-0.0123(0.0235)	0.012(0.0224)
	lifestyle and environment - Alcohol:				
1558	Alcohol intake frequency	0.0024(0.0172)	0.0037(0.0153)	-0.0089(0.0168)	-0.0088(0.0155)
1598	Average weekly spirits intake	0.001(0.0194)	-0.004(0.0181)	-0.003(0.0198)	0.0114(0.019)
1628	Alcohol intake versus 10 years previously	-0.0012(0.0164)	0.0038(0.0151)	0.0011(0.0168)	-0.0096(0.016)
	Lifestyle and environment - Diet:				
1309	Fresh fruit intake	-0.0036(0.0158)	0.0004(0.0145)	0.0091(0.016)	-0.0022(0.0153)
1319	Dried fruit intake	-0.0031(0.0156)	0.007(0.0187)	0.0092(0.0161)	-0.0077(0.0162)
1329	Oily fish intake	0.0015(0.0151)	0.004(0.0141)	-0.0091(0.0157)	-0.0092(0.0151)
1339	Non-oily fish intake	0.0022(0.015)	0.0039(0.0139)	-0.0092(0.0157)	-0.0092(0.015)
1349	Processed meat intake	-0.001(0.0165)	0.0032(0.0149)	0.009(0.0164)	-0.009(0.0154)
1478	Salt added to food	0.0048(0.0167)	-0.0038(0.0153)	-0.0089(0.0165)	0.0089(0.0157)
1518	Hot drink temperature	-0.0027(0.0167)	0.0013(0.0151)	0.0033(0.0166)	-0.0002(0.0156)

1528	Water intake	-0.0034(0.0171)	-0.0037(0.0154)	0.0028(0.0168)	0.0089(0.0157)
1548	Variation in diet	-0.0027(0.0156)	0.0038(0.0143)	0.009(0.0159)	-0.0092(0.0152)
	Lifestyle and environment - Electronic device use:				
1110	Length of mobile phone use	0.002(0.0162)	0.0032(0.0148)	-0.0091(0.0163)	-0.006(0.0155)
1130	Hands-free device/speakerphone use with mobile phone in last 3 month	0.0043(0.0163)	-0.0059(0.0171)	-0.01(0.017)	0.0095(0.0169)
2237	Plays computer games	0.0037(0.0151)	0.0031(0.014)	-0.0091(0.0157)	-0.0092(0.0151)
	Lifestyle and environment - Sexual factors:				
2139	Age first had sexual intercourse	0.0034(0.0169)	-0.0029(0.0156)	-0.0097(0.0171)	0.0067(0.0164)
	Lifestyle and environment - Sleep:				
1160	Sleep duration	-0.0077(0.0161)	-0.0063(0.0187)	0.0089(0.016)	0.0076(0.0162)
1170	Getting up in morning	-0.0043(0.0175)	0.0069(0.0157)	0.0088(0.0169)	-0.0088(0.0159)
1180	Morning/evening person (chronotype)	-0.0003(0.0184)	-0.0064(0.0186)	0.0034(0.0177)	0.0084(0.0169)
1190	Nap during day	-0.0037(0.0153)	0.0045(0.0141)	0.0092(0.0159)	-0.0091(0.0151)
1200	Sleeplessness / insomnia	0.0016(0.0151)	-0.0026(0.014)	-0.0092(0.0157)	0.0092(0.0151)
	Lifestyle and environment - Smoking:				
1249	Past tobacco smoking	-0.0064(0.0175)	0.0037(0.0158)	-0.0095(0.0174)	-0.0095(0.0164)
20116	Smoking status	-0.0044(0.0167)	-0.0005(0.015)	0.009(0.0166)	-0.0008(0.0157)
	Lifestyle and environment - Sun exposure:				
1717	Skin colour	0.0003(0.0185)	-0.0012(0.0166)	-0.0086(0.0173)	0.0086(0.0161)
	Early life factors:				
1687	Comparative body size at age 10	-0.0041(0.0188)	-0.0029(0.0169)	0.0087(0.0176)	0.0086(0.0164)
1697	Comparative height size at age 10	-0.0011(0.02)	0.0022(0.0179)	0.0082(0.018)	-0.0082(0.0168)
	Female-specific factors:				
2714	Age when periods started (menarche)	-0.0081(0.0227)	-0.0057(0.0206)	0.0114(0.0218)	0.0115(0.0206)
2734	Number of live births	0.0013(0.0208)	0.004(0.0189)	-0.0116(0.0208)	-0.0116(0.0197)
2744	Birth weight of first child	-0.0014(0.0244)	-0.0046(0.0226)	0.0138(0.0248)	0.0142(0.0236)
	Male-specific factors:				
2375	Relative age of first facial hair	0.0038(0.0263)	-0.0081(0.0243)	-0.0151(0.0267)	0.015(0.0255)
2395	Hair/balding pattern	-0.0081(0.0293)	0.0074(0.0264)	0.0073(0.0277)	-0.0138(0.0259)
	Psychosocial factors - Mental health:				
1940	Irritability	-0.0005(0.0154)	-0.0019(0.0145)	0.0017(0.0161)	0.006(0.0155)
1960	Fed-up feelings	0.0017(0.0158)	-0.0034(0.0146)	-0.0093(0.0162)	0.0093(0.0155)
2000	Worry too long after embarrassment	0.0046(0.0045)	-0.0027(0.0143)	-0.0094(0.0049)	0.0095(0.0154)
20127	Neuroticism score	0.0028(0.0171)	0.0032(0.0161)	-0.0106(0.0179)	-0.0106(0.0173)

2070	Frequency of tenseness / restlessness	0.0015(0.0161)	0.0037(0.0144)	-0.0094(0.0164)	-0.0094(0.0153)
2080	Frequency of tiredness / lethargy in last 2 weeks	-0.0009(0.0153)	0.0045(0.0142)	0.0028(0.0159)	-0.0094(0.0153)
	Cognitive function - Reaction time:				
404	Duration to first press of snap-button in each round	0.0064(0.0238)	0.0094(0.0238)	-0.0144(0.0247)	-0.0139(0.0242)
	Health and medical history:				
2217	Age started wearing glasses or contact lenses	0.0061(0.02)	-0.0055(0.0179)	-0.0096(0.0189)	0.0095(0.0177)
2247	Hearing difficulty/problems	0.003(0.0162)	0.0006(0.0148)	-0.0094(0.0164)	-0.0008(0.0156)
2443	Diabetes diagnosed by doctor	-0.0046(0.0168)	0.002(0.0152)	0.009(0.0166)	-0.0058(0.0157)
	Sociodemographics - Education:				
6138	Qualifications	-0.0031(0.0183)	-0.0061(0.0164)	0.0086(0.0172)	0.0086(0.016)
	Sociodemographics - Household:				
699	Length of time at current address	-0.0055(0.0206)	-0.0005(0.0143)	0.0078(0.0173)	-0.0005(0.0152)
738	Average total household income before tax	-0.0015(0.0164)	-0.0033(0.0154)	0.0099(0.017)	0.0099(0.0164)

Table S8. Genetic and residual covariances between 70 UKBB phenotypes and PC1/PC2 estimated by bivariate GREML model across POP1+POP3. SE denotes standard error.

UKBB data field	Phenotypes	Genetic covariance with PC1 (SE)	Genetic covariance with PC2 (SE)	Residual covariance with PC1 (SE)	Residual covariance with PC2 (SE)
	Physical measures - Anthropometry:				
48	Waist circumference	-0.0187(0.1147)	0.0878(0.1339)	0.0368(0.1025)	-0.0945(0.1111)
49	Hip circumference	-0.0234(0.0909)	0.054(0.1061)	0.0383(0.0802)	-0.0554(0.087)
50	Standing height	-0.0004(0.0682)	-0.07(0.0781)	0.0132(0.0535)	0.064(0.0579)
20015	Sitting height	0.0048(0.0378)	-0.0333(0.0437)	-0.0003(0.0313)	0.0335(0.034)
21001	Body mass index (BMI)	-0.0065(0.0467)	0.0386(0.0545)	0.0113(0.0412)	-0.0398(0.0446)
21002	Weight	-0.0201(0.141)	0.0719(0.1643)	0.0387(0.1229)	-0.0758(0.1334)
	Physical measures - Blood pressure:				
102	Pulse rate, automated reading	-0.0656(0.1079)	-0.0625(0.1258)	0.0803(0.0985)	0.0668(0.1063)
4079	Diastolic blood pressure, automated reading	-0.0581(0.0982)	0.0352(0.1146)	0.065(0.09)	-0.0238(0.0971)
4080	Systolic blood pressure, automated reading	-0.0595(0.1685)	0.014(0.1966)	0.0614(0.1548)	0.0069(0.1671)
	Physical measures - Bone-densitometry of heel:				
78	Heel bone mineral density (BMD) T-score, automated	-0.007(0.0152)	0.016(0.0176)	0.0085(0.0136)	-0.0158(0.0147)
3143	Ankle spacing width	-0.0088(0.0483)	0.0348(0.0559)	0.0107(0.0441)	-0.0384(0.0473)
3144	Heel Broadband ultrasound attenuation, direct entry	-0.082(0.2359)	0.2011(0.273)	0.111(0.2111)	-0.1974(0.2271)
3147	Heel quantitative ultrasound index (QUI), direct entry	-0.1238(0.2696)	0.2827(0.312)	0.1502(0.2415)	-0.2802(0.2597)
3148	Heel bone mineral density (BMD)	-0.0008(0.0017)	0.0019(0.002)	0.001(0.0015)	-0.0018(0.0016)
	lifestyle and environment - Alcohol:				
1558	Alcohol intake frequency	0.0066(0.0132)	0.0102(0.0154)	-0.0079(0.0124)	-0.0109(0.0133)
	Lifestyle and environment - Diet:				
1309	Fresh fruit intake	-0.0034(0.0135)	-0.0155(0.0157)	0.0039(0.013)	0.0181(0.0139)
1319	Dried fruit intake	0.0165(0.0162)	-0.0263(0.0189)	-0.0214(0.0157)	0.0299(0.0168)
1329	Oily fish intake	0.0055(0.0077)	-0.0001(0.009)	-0.0074(0.0074)	0.0001(0.0079)
1339	Non-oily fish intake	0.0009(0.0066)	-0.002(0.0077)	-0.0012(0.0064)	0.0024(0.0068)
1349	Processed meat intake	-0.0004(0.0088)	0.0024(0.0103)	0.0007(0.0084)	-0.0027(0.009)
1359	Poultry intake	-0.0044(0.0077)	0.0028(0.0089)	0.0062(0.0074)	-0.0033(0.0079)
1369	Beef intake	-0.0046(0.007)	0.0045(0.0082)	0.0064(0.0068)	-0.0051(0.0073)
1379	Lamb/mutton intake	-0.0014(0.006)	-0.0026(0.0069)	0.0019(0.0057)	0.0031(0.0061)
1408	Cheese intake	0.0011(0.0093)	-0.0122(0.0108)	-0.0014(0.009)	0.0138(0.0096)

1458	Cereal intake	-0.0079(0.0242)	0.0034(0.0282)	0.0102(0.023)	-0.0048(0.0247)
1478	Salt added to food	-0.0017(0.0076)	-0.0004(0.0088)	0.0023(0.0071)	0.0008(0.0077)
1518	Hot drink temperature	-0.0011(0.0051)	0.0004(0.0059)	0.0011(0.0048)	-0.0005(0.0051)
1528	Water intake	0.0036(0.0205)	-0.0192(0.0239)	-0.0047(0.0196)	0.0216(0.021)
	Lifestyle and environment - Electronic device use:				
1110	Length of mobile phone use	0.0008(0.0114)	0.002(0.0133)	-0.0011(0.0108)	-0.0023(0.0116)
2237	Plays computer games	-0.0001(0.0044)	0.0014(0.0051)	0.0003(0.0042)	-0.0016(0.0045)
	Lifestyle and environment - Sexual factors:				
2139	Age first had sexual intercourse	-0.021(0.0366)	0.0219(0.0429)	0.0241(0.0335)	-0.023(0.0364)
	Lifestyle and environment - Sleep:				
1160	Sleep duration	-0.0027(0.0093)	0.0112(0.0109)	0.0037(0.0088)	-0.0124(0.0095)
1170	Getting up in morning	0.002(0.007)	0.0013(0.0081)	-0.0025(0.0064)	-0.001(0.0069)
1180	Morning/evening person (chronotype)	0.0029(0.0093)	-0.0005(0.0109)	-0.0029(0.0085)	0.0004(0.0092)
1190	Nap during day	0.0014(0.0052)	-0.001(0.006)	-0.0019(0.0048)	0.001(0.0052)
1200	Sleeplessness / insomnia	0.0009(0.0063)	0.0014(0.0074)	-0.001(0.0059)	-0.0019(0.0064)
1210	Snoring	0.0037(0.0042)	0.0003(0.0049)	-0.0049(0.004)	-0.0003(0.0043)
	Lifestyle and environment - Smoking:				
1239	Current tobacco smoking	-0.0017(0.0036)	0.0002(0.0041)	0.0023(0.0034)	-0.0003(0.0037)
1249	Past tobacco smoking	-0.003(0.0122)	-0.0008(0.0143)	0.0039(0.0116)	0.0008(0.0125)
20116	Smoking status	-0.0003(0.0063)	-0.0008(0.0073)	0.0006(0.0058)	0.0008(0.0063)
20160	Ever smoked	0.0019(0.0043)	-0.0008(0.0051)	-0.0024(0.0041)	0.0004(0.0044)
	Lifestyle and environment - Sun exposure:				
1717	Skin colour	0.0002(0.0055)	0.0016(0.0064)	-0.0015(0.0049)	-0.0012(0.0053)
	Early life factors:				
1687	Comparative body size at age 10	0.0016(0.0031)	0.0007(0.0036)	-0.002(0.0028)	-0.0007(0.0031)
1697	Comparative height size at age 10	-0.0007(0.0034)	-0.0006(0.0039)	0.0007(0.0029)	0.0004(0.0031)
20022	Birth weight	0.002(0.0075)	0.0011(0.0087)	-0.0021(0.0071)	-0.0012(0.0076)
	Female-specific factors:				
2714	Age when periods started (menarche)	-0.0126(0.0185)	-0.0272(0.0218)	0.0176(0.0175)	0.0305(0.0189)
2734	Number of live births	0.0012(0.0128)	-0.0093(0.0151)	-0.0023(0.0125)	0.0103(0.0134)
	Male-specific factors:				
2375	Relative age of first facial hair	0.0034(0.0065)	-0.0021(0.0074)	-0.0044(0.0059)	0.002(0.0063)
2395	Hair/balding pattern	-0.006(0.0164)	0.0022(0.0188)	0.007(0.0143)	-0.0019(0.0154)
	Psychosocial factors - Mental health:				
1920	Mood swings	0.0012(0.0044)	-0.0003(0.0052)	-0.0014(0.0041)	0.0002(0.0044)

1930	Miserableness	0.0018(0.0042)	0.0058(0.0049)	-0.0024(0.004)	-0.0066(0.0043)
1940	Irritability	0.0004(0.0041)	-0.0005(0.0047)	-0.0002(0.0038)	0.0006(0.0041)
1950	Sensitivity / hurt feelings	0.0004(0.0042)	0.0029(0.0049)	-0.0006(0.004)	-0.0035(0.0043)
1960	Fed-up feelings	-0.0002(0.0043)	0.0013(0.005)	0.0005(0.004)	-0.0017(0.0043)
1970	Nervous feelings	0.0011(0.0036)	-0.0001(0.0042)	-0.0015(0.0034)	0(0.0037)
1980	Worrier / anxious feelings	0.001(0.0044)	0.0007(0.0051)	-0.0014(0.0041)	-0.0009(0.0044)
1990	Tense / 'highly strung'	-0.0003(0.0035)	0.0008(0.0041)	0.0004(0.0032)	-0.001(0.0035)
2000	Worry too long after embarrassment	0.0016(0.0044)	-0.0025(0.0051)	-0.0021(0.0041)	0.0028(0.0044)
20127	Neuroticism score	0.0078(0.0315)	-0.0095(0.0371)	-0.0092(0.0295)	0.0082(0.032)
2030	Guilty feelings	0.0016(0.0039)	-0.0015(0.0046)	-0.002(0.0037)	0.0016(0.004)
2040	Risk taking	-0.0007(0.004)	-0.0001(0.0046)	0.001(0.0038)	0.0001(0.0041)
2080	Frequency of tiredness / lethargy in last 2 weeks	-0.0055(0.0073)	0.0008(0.0086)	0.007(0.0069)	-0.0011(0.0075)
2090	Seen doctor (GP) for nerves, anxiety, tension or depression	0.001(0.0039)	0.0032(0.0046)	-0.0013(0.0038)	-0.0037(0.0041)
	Cognitive function - Reaction time:				
20023	Mean time to correctly identify matches	-0.159(0.91)	0.5117(1.0597)	0.226(0.863)	-0.6117(0.9266)
	Health and medical history:				
2217	Age started wearing glasses or contact lenses	0.015(0.1613)	-0.1278(0.1888)	-0.0136(0.1481)	0.1421(0.1604)
2443	Diabetes diagnosed by doctor	0.0001(0.0018)	-0.0004(0.0021)	-0.0001(0.0017)	0.0004(0.0018)
2463	Fractured/broken bones in last 5 years	0.0008(0.0026)	-0.0045(0.003)	-0.0012(0.0025)	0.0052(0.0026)
	Sociodemographics - Education:				
6138	Qualifications	0.004(0.0093)	-0.0085(0.0108)	-0.0062(0.0083)	0.0096(0.009)
	Sociodemographics - Household:				
738	Average total household income before tax	-0.0039(0.0101)	-0.0088(0.0117)	0.0048(0.0096)	0.0101(0.0103)
	Verbal interview - Medical conditions:				
135	Number of self-reported non-cancer illnesses	0.0001(0.0156)	-0.0047(0.0182)	0.0015(0.0147)	0.0049(0.0158)

Table S9. **Genetic variance, interaction variance and their covariance component estimates for 70 UKBB phenotypes with significant heritabilities across POP1+POP2 with the covariate PC1**. The estimates which were not within the valid parameter space were excluded for follow-up analyses as shown in the column "Note". SE denotes standard error. DF denotes degree of freedom.

UKBB data field	$var(\mathbf{g_0})$ (SE)	$var(\mathbf{g_1})$ (SE)	$cov(\mathbf{g_0},\mathbf{g_1})$ (SE)	$var(\mathbf{e_0})$ (SE)	Note	P-value by LRT comparing with baseline model (DF =2)
48	22.8234(3.4160)	1.0785(0.8583)	-2.8866(1.0905)	115.2992(3.6088)		0.0220
49	16.1120(2.1416)	0.3507(0.5114)	-1.2321(0.6705)	69.6114(2.2349)		0.1322
50	19.3852(1.0527)	-0.0938(0.2146)	0.2898(0.3109)	19.9075(0.9541)	Excluded	
20015	4.5722(0.3539)	-0.1247(0.0649)	0.1097(0.1028)	9.1564(0.3431)	Excluded	
21001	4.6117(0.5676)	0.2398(0.1522)	-0.3711(0.1822)	17.8795(0.5895)		0.1285
21002	43.0297(4.9553)	2.8555(1.3309)	-5.5148(1.5958)	151.7953(5.1194)		0.0029
102	19.6191(3.1902)	1.5100(0.8223)	-3.8713(0.9883)	103.7211(3.3880)		0.0002
4079	13.3974(2.7753)	-0.4101(0.6394)	0.6785(0.8590)	95.8879(2.9699)	Excluded	
4080	43.8004(8.2129)	1.7493(2.1498)	-4.9706(2.5723)	274.5824(8.8145)		0.1119
78	0.4026(0.0676)	0.0263(0.0189)	0.0416(0.0185)	1.0632(0.0679)		9.39E-07
3143	2.7502(0.6788)	-0.0278(0.1648)	0.4103(0.1829)	12.7605(0.7007)	Excluded	
3144	85.2801(16.0444)	6.6743(4.6119)	10.4970(4.4641)	267.7478(16.2238)		2.87E-07
3147	126.3220(21.2130)	8.2347(5.9147)	13.0431(5.8075)	333.5802(21.2942)		9.40E-07
3148	0.0048(0.0008)	0.0003(0.0002)	0.0006(0.0002)	0.0135(0.0008)		1.58E-07
1558	0.1547(0.0528)	-0.0036(0.0125)	0.0264(0.0168)	2.1348(0.0587)	Excluded	
1598	1.9868(0.7932)	6.5790(0.4870)	-4.4794(0.4369)	31.0538(1.0070)	Excluded	
1309	0.0183(0.0348)	-0.0224(0.0072)	0.0472(0.0112)	2.0163(0.0402)	Excluded	
1329	0.0669(0.0187)	0.0049(0.0049)	-0.0090(0.0061)	0.7365(0.0208)		0.3285
1369	0.0500(0.0167)	-0.0032(0.0043)	0.0220(0.0055)	0.6616(0.0184)	Excluded	
1379	0.0275(0.0117)	-0.0027(0.0034)	0.0212(0.0040)	0.4843(0.0130)	Excluded	
1458	0.9810(0.1847)	-0.0285(0.0422)	0.0126(0.0576)	6.7208(0.1981)	Excluded	
1478	0.0819(0.0195)	-0.0030(0.0049)	0.0313(0.0063)	0.7404(0.0212)	Excluded	
1488	0.7017(0.0510)	0.6597(0.0380)	-0.7585(0.0436)	6.4842(0.1076)	Excluded	
1518	0.0435(0.0084)	-0.0033(0.0016)	0.0113(0.0024)	0.3076(0.0089)	Excluded	
1528	0.1598(0.0832)	-0.0406(0.0174)	0.1469(0.0256)	4.2004(0.0949)	Excluded	
1548	0.0248(0.0086)	-0.0017(0.0020)	0.0038(0.0027)	0.3413(0.0095)	Excluded	
1120	0.1099(0.0425)	0.0136(0.0120)	0.0077(0.0134)	1.4478(0.0469)		0.0799
2237	0.0249(0.0061)	0.0039(0.0020)	-0.0034(0.0021)	0.2304(0.0068)		0.1408
2139	1.5527(0.4033)	0.3563(0.1283)	-0.1990(0.1348)	13.1435(0.4406)		0.0047
2149	-36.1697(19.0655)	-5.4504(2.0441)	36.1221(5.8221)	1143.5765(23.37)	Excluded	
2159	0.0000(0.0006)	-0.0003(0.0001)	0.0010(0.0002)	0.0297(0.0006)	Excluded	

1160	0.0834(0.0267)	0.0218(0.0091)	-0.0063(0.0093)	1.0469(0.0299)		0.0073
1170	0.0601(0.0146)	-0.0041(0.0037)	0.0183(0.0048)	0.5567(0.0159)	Excluded	
1180	0.0822(0.0244)	-0.0101(0.0053)	0.0252(0.0075)	0.8692(0.0265)	Excluded	
1190	0.0291(0.0080)	0.0072(0.0024)	-0.0096(0.0026)	0.3010(0.0089)		0.0006
1200	0.0539(0.0124)	-0.0027(0.0030)	0.0052(0.0039)	0.4670(0.0134)	Excluded	
1239	0.0004(0.0023)	-0.0012(0.0005)	0.0036(0.0008)	0.1299(0.0027)	Excluded	
1249	0.2571(0.0390)	0.0632(0.0133)	-0.1324(0.0146)	1.4763(0.0418)	Excluded	
20116	0.0700(0.0121)	0.0057(0.0035)	-0.0032(0.0040)	0.4228(0.0130)		0.1870
20160	0.0303(0.0058)	0.0047(0.0016)	-0.0082(0.0019)	0.1972(0.0063)		0.0001
1717	0.0263(0.0075)	-0.0023(0.0017)	0.0162(0.0024)	0.3254(0.0083)	Excluded	
1687	0.0146(0.0029)	-0.0005(0.0008)	0.0026(0.0009)	0.1034(0.0031)	Excluded	
1697	0.0270(0.0028)	-0.0001(0.0007)	-0.0001(0.0009)	0.0839(0.0029)	Excluded	
20022	0.0490(0.0186)	0.0026(0.0035)	-0.0020(0.0047)	0.3900(0.0194)		0.6955
2714	0.6343(0.1138)	0.0031(0.0209)	-0.0263(0.0285)	1.9979(0.1153)		0.5074
2744	0.2355(0.0859)	-0.0113(0.0150)	0.0631(0.0206)	1.3442(0.0887)	Excluded	
2375	0.0399(0.0112)	-0.0033(0.0020)	0.0117(0.0028)	0.1671(0.0114)	Excluded	
2385	0.0139(0.0058)	-0.0027(0.0008)	0.0079(0.0014)	0.0930(0.0060)	Excluded	
2395	0.4116(0.0668)	0.0069(0.0120)	-0.0088(0.0153)	0.7675(0.0659)		0.8129
1920	0.0191(0.0058)	0.0015(0.0015)	-0.0021(0.0018)	0.2195(0.0064)		0.4826
1940	0.0177(0.0052)	-0.0023(0.0011)	0.0056(0.0016)	0.1925(0.0056)	Excluded	
1950	0.0125(0.0057)	-0.0007(0.0013)	0.0012(0.0018)	0.2248(0.0063)	Excluded	
1960	0.0160(0.0056)	0.0012(0.0015)	-0.0011(0.0018)	0.2152(0.0062)		0.7256
1970	0.0127(0.0044)	-0.0017(0.0010)	0.0050(0.0014)	0.1741(0.0049)	Excluded	
1980	0.0247(0.0057)	0.0001(0.0014)	-0.0013(0.0018)	0.2115(0.0062)		0.6595
1990	0.0052(0.0031)	-0.0011(0.0007)	0.0055(0.0010)	0.1351(0.0035)	Excluded	
2000	0.0326(0.0062)	-0.0003(0.0014)	0.0001(0.0019)	0.2118(0.0066)	Excluded	
20127	1.7563(0.3177)	0.0084(0.0695)	0.0255(0.0929)	8.5585(0.3316)		0.8753
2030	0.0204(0.0050)	-0.0011(0.0012)	0.0026(0.0016)	0.1843(0.0054)	Excluded	
2040	0.0124(0.0043)	-0.0019(0.0009)	0.0072(0.0012)	0.1779(0.0047)	Excluded	
2050	0.0123(0.0072)	-0.0025(0.0017)	0.0136(0.0023)	0.3273(0.0081)	Excluded	
2060	0.0113(0.0077)	-0.0022(0.0017)	0.0157(0.0024)	0.3693(0.0087)	Excluded	
2070	0.0106(0.0071)	-0.0031(0.0017)	0.0116(0.0022)	0.3240(0.0080)	Excluded	
2080	0.0707(0.0174)	0.0007(0.0048)	0.0157(0.0058)	0.6382(0.0190)	Excluded	
2090	0.0135(0.0050)	0.0006(0.0012)	-0.0027(0.0016)	0.2007(0.0056)		0.1977
403	0.2533(0.0046)	-0.0006(0.0040)	-0.0029(0.0009)	0.2533(0.0046)	Excluded	
2217	27.0236(7.4244)	-2.3572(1.1177)	-0.3735(2.0760)	242.8999(7.9144)	Excluded	
6138	0.1583(0.0246)	0.0768(0.0101)	-0.0688(0.0086)	0.7846(0.0265)		1.96E-18

728	0.0338(0.0165)	0.0009(0.0045)	-0.0014(0.0054)	0.6696(0.0185)		0.9670
20009	19.0474(6.7765)	1.0624(1.3542)	-3.2719(1.8475)	181.4122(7.2165)		0.1779

Table S10. **Genetic variance, interaction variance and their covariance component estimates for 70 UKBB phenotypes with significant heritabilities across POP1+POP2 with the covariate PC2.** The estimates which were not within the valid parameter space were excluded for follow-up analyses as shown in the column "Note". SE denotes standard error. DF denotes degree of freedom.

UKBB data field	$var(\mathbf{g_0})$ (SE)	$var(\mathbf{g_1})$ (SE)	$cov(\mathbf{g_0}, \mathbf{g_1})$ (SE)	$var(\mathbf{e_0})$ (SE)	Note	P-value by LRT comparing with baseline model (DF =2)
48	22.6677(3.4011)	0.6079(0.4699)	3.5679(1.0350)	115.9131(3.5460)		0.0008
49	16.0363(2.1375)	0.4629(0.3077)	2.2087(0.6575)	69.5760(2.2019)		0.0020
50	19.4046(1.0518)	-0.0410(0.1465)	-0.4103(0.3192)	19.8373(0.9442)	Excluded	
20015	4.5379(0.3536)	0.0759(0.0575)	0.0999(0.1115)	8.9874(0.3409)		0.3857
21001	4.6304(0.5666)	0.1250(0.0825)	0.5573(0.1746)	17.9787(0.5791)		0.0040
21002	42.9272(4.9474)	1.3950(0.7474)	5.2355(1.5208)	153.3097(5.0251)		0.0020
102	19.4977(3.1888)	0.6285(0.4871)	2.5351(0.9932)	104.6823(3.3361)		0.0308
4079	13.2579(2.7734)	0.1359(0.3432)	0.7811(0.8411)	95.4995(2.9324)		0.6326
4080	43.2231(8.1925)	1.4060(1.1705)	5.3977(2.4994)	275.4511(8.6274)		0.0920
78	0.4017(0.0681)	-0.0072(0.0061)	-0.0179(0.0158)	1.0985(0.0675)	Excluded	
3143	2.7566(0.6796)	-0.1655(0.0539)	-0.3505(0.1637)	12.9057(0.6917)	Excluded	
3144	84.7201(16.1744)	-1.1356(1.5932)	-4.0006(3.8517)	276.3461(16.1749)	Excluded	
3147	126.0446(21.3600)	-2.2620(1.9058)	-5.6293(4.9698)	344.6565(21.1687)	Excluded	
3148	0.0048(0.0008)	-0.0001(0.0001)	-0.0003(0.0002)	0.0139(0.0008)	Excluded	
1558	0.1525(0.0528)	0.0148(0.0096)	0.0161(0.0171)	2.1192(0.0581)		0.1938
1598	0.7563(0.9430)	16.6010(1.0157)	4.1934(0.4631)	25.6094(1.1035)	Excluded	
1309	0.0108(0.0329)	-0.0148(0.0035)	-0.0529(0.0098)	1.9700(0.0379)	Excluded	
1329	0.0669(0.0187)	-0.0010(0.0027)	0.0028(0.0061)	0.7423(0.0204)	Excluded	
1369	0.0521(0.0167)	-0.0030(0.0027)	-0.0194(0.0055)	0.6595(0.0182)	Excluded	
1379	0.0273(0.0117)	0.0053(0.0027)	-0.0060(0.0041)	0.4768(0.0130)		2.73E-05
1458	0.9782(0.1847)	0.0012(0.0250)	0.0310(0.0576)	6.6938(0.1961)		0.7890
1478	0.0796(0.0196)	0.0007(0.0036)	-0.0145(0.0066)	0.7393(0.0211)	Excluded	
1488	0.4535(0.1724)	0.0406(0.0290)	0.1418(0.0556)	7.3141(0.1924)	Excluded	
1518	0.0423(0.0084)	-0.0013(0.0012)	-0.0090(0.0026)	0.3069(0.0089)	Excluded	
1528	0.7063(0.1312)	-0.0328(0.0212)	-0.3455(0.0422)	4.9554(0.1371)	Excluded	
1548	0.0247(0.0086)	0.0005(0.0014)	-0.0020(0.0028)	0.3392(0.0094)		0.4123
1120	0.1081(0.0425)	0.0093(0.0072)	0.0078(0.0135)	1.4543(0.0461)		0.2894
2237	0.0256(0.0061)	0.0004(0.0009)	0.0035(0.0019)	0.2333(0.0066)	Excluded	
2139	1.4802(0.4017)	0.3641(0.0929)	0.4340(0.1365)	13.2176(0.4311)		0.0001
2149	-6.7960(19.5429)	-8.5329(2.3998)	-38.0657(5.4242)	1128.2480(23.51)	Excluded	
2159	0.0012(0.0006)	-0.0003(0.0001)	-0.0016(0.0002)	0.0381(0.0007)	Excluded	

1160	0.0828(0.0267)	0.0235(0.0067)	0.0151(0.0097)	1.0463(0.0293)		0.0001
1170	0.0616(0.0145)	-0.0048(0.0022)	-0.0250(0.0048)	0.5562(0.0156)	Excluded	
1180	0.0827(0.0243)	-0.0056(0.0030)	-0.0265(0.0073)	0.8643(0.0260)	Excluded	
1190	0.0285(0.0081)	0.0023(0.0014)	0.0064(0.0026)	0.3064(0.0088)		0.0595
1200	0.0534(0.0124)	0.0004(0.0018)	-0.0001(0.0040)	0.4644(0.0132)		0.9543
1239	0.0146(0.0041)	0.0014(0.0010)	-0.0135(0.0014)	0.1765(0.0043)	Excluded	
1249	0.2777(0.0417)	0.0272(0.0075)	0.0992(0.0133)	1.4904(0.0443)	Excluded	
20116	0.0699(0.0121)	0.0054(0.0024)	0.0056(0.0040)	0.4232(0.0128)		0.0233
20160	0.0308(0.0058)	0.0017(0.0009)	0.0056(0.0018)	0.1996(0.0062)		0.0072
1717	0.0460(0.0094)	0.0010(0.0019)	-0.0100(0.0032)	0.3453(0.0101)	Excluded	
1687	0.0147(0.0029)	-0.0005(0.0004)	-0.0018(0.0009)	0.1033(0.0031)	Excluded	
1697	0.0270(0.0028)	-0.0001(0.0004)	-0.0004(0.0009)	0.0840(0.0028)	Excluded	
20022	0.0480(0.0184)	-0.0012(0.0012)	0.0071(0.0041)	0.3949(0.0192)	Excluded	
2714	0.6329(0.1137)	0.0120(0.0155)	0.0264(0.0297)	1.9899(0.1145)		0.6695
2744	0.2488(0.0864)	0.0059(0.0127)	-0.0211(0.0211)	1.3147(0.0884)		0.1341
2375	0.0385(0.0112)	-0.0018(0.0012)	-0.0096(0.0027)	0.1670(0.0113)	Excluded	
2385	0.0185(0.0063)	-0.0019(0.0004)	-0.0075(0.0013)	0.0921(0.0064)	Excluded	
2395	0.4137(0.0668)	-0.0003(0.0064)	0.0056(0.0152)	0.7728(0.0653)	Excluded	
1920	0.0193(0.0058)	0.0010(0.0009)	0.0023(0.0018)	0.2198(0.0063)		0.3940
1940	0.0174(0.0052)	-0.0012(0.0006)	-0.0037(0.0016)	0.1918(0.0056)	Excluded	
1950	0.0124(0.0057)	0.0001(0.0009)	-0.0004(0.0018)	0.2241(0.0062)		0.8834
1960	0.0162(0.0056)	0.0007(0.0009)	0.0022(0.0018)	0.2155(0.0061)		0.4926
1970	0.0123(0.0044)	0.0002(0.0006)	-0.0006(0.0014)	0.1727(0.0048)		0.6717
1980	0.0247(0.0057)	0.0005(0.0009)	0.0013(0.0019)	0.2111(0.0062)		0.7755
1990	0.0122(0.0039)	-0.0011(0.0006)	-0.0076(0.0013)	0.1478(0.0042)	Excluded	
2000	0.0326(0.0062)	0.0003(0.0009)	0.0008(0.0019)	0.2112(0.0065)		0.9192
20127	1.7552(0.3175)	0.0395(0.0438)	0.0757(0.0920)	8.5297(0.3276)		0.5963
2030	0.0205(0.0050)	-0.0008(0.0006)	-0.0015(0.0016)	0.1839(0.0053)	Excluded	
2040	0.0141(0.0046)	-0.0018(0.0006)	-0.0084(0.0015)	0.1840(0.0050)	Excluded	
2050	0.0415(0.0097)	0.0020(0.0020)	-0.0285(0.0032)	0.3642(0.0101)	Excluded	
2060	0.0406(0.0108)	0.0051(0.0028)	-0.0282(0.0038)	0.4119(0.0115)	Excluded	
2070	0.0403(0.0103)	-0.0032(0.0015)	-0.0191(0.0031)	0.3799(0.0109)	Excluded	
2080	0.0709(0.0174)	0.0010(0.0029)	-0.0087(0.0058)	0.6380(0.0187)	Excluded	
2090	0.0134(0.0050)	0.0011(0.0008)	0.0033(0.0016)	0.2003(0.0055)		0.1522
403	0.0011(0.0039)	-0.0022(0.0004)	-0.0060(0.0012)	0.2660(0.0046)	Excluded	
2217	26.5141(7.4474)	-1.0862(0.8221)	0.0062(2.1969)	242.1076(7.9367)	Excluded	
6138	0.1752(0.0237)	0.0459(0.0063)	0.0862(0.0084)	0.7995(0.0250)		3.22E-23

728	0.0337(0.0165)	0.0055(0.0031)	0.0036(0.0055)	0.6650(0.0182)		0.0897
20009	18.6624(6.7718)	0.9099(1.0368)	2.4535(1.9616)	181.8959(7.1638)		0.4897

Table S11. **Genetic variance, two interaction variances and their covariance component estimates for ten UKBB phenotypes across POP1+POP2 with simultaneous covariates PC1 and PC2**. The estimates which were not within the valid parameter space were excluded for follow-up analyses as shown in the column "Note". SE denotes standard error. DF denotes degree of freedom.

UKBB data field	$var(\mathbf{g_0})$ (SE)	$var(\mathbf{g_1})$ (SE)	$cov(\mathbf{g_0}, \mathbf{g_1})$ (SE)	$var(\mathbf{g_2})$ (SE)	$cov(\mathbf{g_0}, \mathbf{g_2})$ (SE)	$var(\mathbf{e_0})$ (SE)	Note	P-value by LRT comparing with null model (DF =4)
102	19.6275(3.1920)	1.6468(0.9220)	-4.5356(1.3220)	-0.1943(0.5102)	-0.9667(1.3264)	103.7767(3.3897)	Excluded	
78	0.4053(0.0676)	0.0247(0.0216)	0.0695(0.0255)	-0.0067(0.0074)	0.0346(0.0232)	1.0682(0.0679)	Excluded	
3144	84.8875(16.0285)	6.4843(5.3411)	18.3183(6.1430)	-1.0605(1.8600)	10.6128(5.5819)	269.2638(16.2269)	Excluded	
3147	127.1609(21.2069)	7.7508(6.7659)	21.8139(7.9958)	-2.0979(2.3355)	10.8653(7.2771)	335.1489(21.2930)	Excluded	
3148	0.0048(0.0008)	0.0003(0.0003)	0.0009(0.0003)	-0.0001(0.0001)	0.0004(0.0003)	0.0135(0.0008)	Excluded	
1160	0.0824(0.0267)	0.0061(0.0084)	-0.0017(0.0119)	0.0213(0.0073)	0.0145(0.0130)	1.0426(0.0297)		0.0003
20160	0.0304(0.0058)	0.0043(0.0018)	-0.0076(0.0024)	0.0005(0.0009)	0.0009(0.0024)	0.1971(0.0063)		0.0005
1379	0.0276(0.0116)	-0.0051(0.0030)	0.0235(0.0048)	0.0065(0.0028)	0.0085(0.0053)	0.4801(0.0129)	Excluded	
2139	1.4745(0.4017)	0.1370(0.1306)	0.0349(0.1669)	0.3112(0.1006)	0.4877(0.1743)	13.1333(0.4386)		0.0002
6138	0.1751(0.0223)	0.0619(0.0115)	-0.0237(0.0111)	0.0262(0.0064)	0.0816(0.0105)	0.7579(0.0246)	Excluded	

Table S12. **Comparison of LRT significance results with/without additional confounders for ten phenotypes with significant interaction across POP1+POP2.** In bold we marked the significant ones by *P* < 3.57E-4 after Bonferroni correction. The estimates which were not within the valid parameter space were excluded. DF denotes degree of freedom.

UKBB data-field	Phenotypes	*P*-value by LRT (G×P RNM with PC1 vs. null model) (DF =2) for phenotypes adjusted by basic confounders	*P*-value by LRT (G×P RNM with PC1 vs. null model) (DF=2) for phenotypes adjusted by basic and additional confounders	*P*-value by LRT (G×P RNM with PC2 vs. null model) (DF=2) for phenotypes adjusted by basic confounders	*P*-value by LRT (G×P RNM with PC2 vs. null model) (DF =2) for phenotypes adjusted by basic and additional confounders
102	Pulse rate, automated reading	**0.0002**	0.0021	0.0308	0.0887
78	Heel bone mineral density (BMD) T-score, automated	**9.39E-07**	**1.33E-08**	Excluded	Excluded
3144	Heel Broadband ultrasound attenuation, direct entry	**2.87E-07**	**1.70E-07**	Excluded	Excluded
3147	Heel quantitative ultrasound index (QUI), direct entry	**9.40E-07**	**1.33E-08**	Excluded	Excluded
3148	Heel bone mineral density (BMD)	**1.58E-07**	**2.19E-09**	Excluded	Excluded
1160	Sleep duration	0.0073	0.2187	**0.0001**	0.0179
20160	Ever smoked	**0.0001**	0.6531	0.0072	0.9341
1379	Lamb/mutton intake	Excluded	Excluded	**2.73E-05**	0.0056
2139	Age first had sexual intercourse	0.0047	0.0010	**0.0001**	**0.0001**
6138	Qualifications	**1.96E-18**	**1.21E-12**	**3.22E-23**	**3.76E-19**

Notes: the basic confounders of fixed effects are sex, year of birth, age at recruitment, genotype measurement batch, UKB assessment centre and the first 20 ancestry PCs provided by UKB.

For phenotypes data-field 102, additional confounders are: “Townsend deprivation index at recruitment (data-field 189)”, “major dietary changes in the last 5 years (data-field 1538)”, “variation in diet (data-field 1548)”, “smoking status (data-field 20116)” and “alcohol drinker status (data-field 20117)”.

For phenotypes data-field 78, 3144, 3147, 3148, additional confounders are: “Townsend deprivation index at recruitment (data-field 189)”, “ankle spacing width (data-field 3143)”, “heel ultrasound method (data-field 19)” and “foot measured for bone density (data-field 3081)”.

For phenotypes data-field 1160, additional confounders are: “Townsend deprivation index at recruitment (data-field 189)”, “residential noise pollution (data-field: 24020-24024)”, “type of accommodation lived in (data-field 670)”, “number in household (data-field 709)”, “current employment status (data-field 6142)”, “morning/evening person (chronotype) (data-field 1180)”, “sleeplessness / insomnia (data-field 1200)” and “daytime dozing/sleeping (narcolepsy) (data-Field 1220)”.

For phenotype data-field 20160, additional confounder is: “Townsend deprivation index at recruitment (data-field 189)”, “exposure to tobacco smoke at home (data-field 1269)” and “exposure to tobacco smoke outside home (data-field 1279)”.

For phenotype data-field 1379, additional confounders are: “Townsend deprivation index at recruitment (data-field 189)”, “major dietary changes in the last 5 years (data-field 1538)”, “variation in diet (data-field 1548)”, “alcohol drinker status (data-field 20117)” and “average total household income before tax (data-field: 738)”.

For phenotype data-field 2139, additional confounders are: “Townsend deprivation index at recruitment (data-field 189)” and “qualifications (data-field 6138)”.

For phenotype data-field 6138, additional confounders are: “Townsend deprivation index at recruitment (data-field: 189)”, “current employment status (data-field: 6142)” and “average total household income before tax (data-field: 738)”.

Table S13. **Results of model comparisons (baseline, G×P/R×P and full) through LRT for two phenotypes across POP1+POP2 with the covariates PC1 and PC2**. The phenotypes were adjusted by basic plus additional confounders of fixed effects and transformed using rank-based INT. DF denotes degree of freedom.

UKBB data field	Phenotype	Covariate	Model comparison	DF	P-value by LRT
2139	Age first had sexual intercourse	PC1	Full versus R×P	2	0.8770
			Full versus G×P	2	0.3778
			Full versus GREML	4	0.0002
			R×P versus GREML	2	2.22E-05
			G×P versus GREML	2	5.16E-05
		PC2	Full versus R×P	2	0.9247
			Full versus G×P	2	0.9901
			Full versus GREML	4	0.0484
			R×P versus GREML	2	0.0131
			G×P versus GREML	2	0.0097
6138	Qualifications	PC1	Full versus R×P	2	4.15E-05
			Full versus G×P	2	5.09E-05
			Full versus GREML	4	2.35E-20
			R×P versus GREML	2	1.13E-17
			G×P versus GREML	2	9.21E-18
		PC2	Full versus R×P	2	0.0032
			Full versus G×P	2	3.39E-09
			Full versus GREML	4	5.64E-31
			R×P versus GREML	2	2.36E-30
			G×P versus GREML	2	2.22E-24

Table S14. Genetic variance, interaction variance and their covariance component estimates for 58 UKBB phenotypes with significant heritabilities across POP2+POP3 with the covariate PC1. The estimates which were not within the valid parameter space are excluded for follow-up analyses as shown in the column "Note" (in bold we marked the significant ones by P < 0.05/116 = 0.0004 after Bonferroni correction). SE denotes standard error. DF denotes degree of freedom.

UKBB data-field	$var(\mathbf{g_0})$ (SE)	$var(\mathbf{g_1})$ (SE)	$cov(\mathbf{g_0}, \mathbf{g_1})$ (SE)	$var(\mathbf{e_0})$ (SE)	Note	*P*-value by LRT comparing with null model (DF=2)
48	29.3531(3.4716)	3.9198(1.0112)	-6.8481(1.1346)	108.7908(3.6147)		**6.92E-09**
49	17.7361(2.1585)	1.4819(0.5692)	-2.9198(0.6875)	68.3066(2.2444)		**0.0001**
50	19.3685(1.0300)	0.2300(0.2268)	-0.1071(0.3125)	19.7821(0.9359)		0.4506
20015	4.7694(0.3326)	-0.0584(0.0696)	0.1839(0.0995)	8.3267(0.3186)	Excluded	
21001	5.2187(0.5645)	0.4888(0.1602)	-0.6964(0.1836)	17.1518(0.5808)		0.0004
21002	50.4472(4.9668)	5.9372(1.4506)	-9.2975(1.6187)	142.5020(5.0576)		**4.05E-08**
102	14.4839(2.9487)	1.3990(0.8005)	-2.6871(0.9507)	104.7696(3.1923)		0.0166
4079	15.4457(2.7790)	-0.2765(0.6064)	0.1227(0.8651)	95.0684(2.9504)	Excluded	
4080	40.1608(7.6905)	-2.7447(1.4987)	2.9719(2.3585)	272.5969(8.2202)	Excluded	
78	0.3206(0.0671)	0.0480(0.0204)	0.0252(0.0187)	1.1281(0.0692)		**3.76E-07**
3143	4.4681(0.7193)	-0.0833(0.1491)	0.4781(0.1809)	10.9806(0.7188)	Excluded	
3144	71.8120(16.1704)	9.3888(4.7703)	8.6593(4.4874)	279.7071(16.6966)		**1.83E-07**
3147	100.5781(21.0580)	15.0530(6.3924)	7.9177(5.8529)	353.9731(21.7207)		**3.77E-07**
3148	0.0041(0.0008)	0.0006(0.0003)	0.0003(0.0002)	0.0140(0.0009)		**2.47E-07**
1558	0.2557(0.0542)	0.0039(0.0123)	0.0041(0.0174)	2.0676(0.0588)		0.7806
1598	2.5854(0.7757)	0.4771(0.1750)	-1.4140(0.2662)	29.4564(0.8877)	Excluded	
1628	0.0160(0.0078)	0.0019(0.0023)	-0.0096(0.0029)	0.4396(0.0096)	Excluded	
1309	0.0197(0.0364)	-0.0244(0.0078)	0.0458(0.0123)	2.1924(0.0423)	Excluded	
1319	0.0391(0.0589)	-0.0319(0.0136)	0.0850(0.0183)	3.1448(0.0681)	Excluded	
1329	0.0384(0.0180)	0.0072(0.0050)	-0.0111(0.0061)	0.7627(0.0204)		0.1723
1339	0.0299(0.0140)	0.0053(0.0041)	-0.0032(0.0047)	0.5921(0.0159)		0.3751
1349	0.0996(0.0269)	0.0055(0.0062)	-0.0116(0.0088)	1.0696(0.0296)		0.4266
1478	0.0820(0.0188)	-0.0020(0.0046)	0.0304(0.0063)	0.7340(0.0205)	Excluded	
1518	0.0326(0.0084)	-0.0041(0.0014)	0.0075(0.0025)	0.3350(0.0091)	Excluded	
1528	0.7051(0.1586)	0.0826(0.0457)	-0.0074(0.0530)	5.9796(0.1736)		0.0261

1548	0.0222(0.0085)	0.0002(0.0021)	-0.0015(0.0028)	0.3542(0.0095)		0.8026
1110	0.1277(0.0347)	0.0109(0.0089)	-0.0479(0.0121)	1.5226(0.0383)	Excluded	
1130	0.0419(0.0188)	0.0106(0.0060)	0.0244(0.0061)	0.6486(0.0206)	Excluded	
2237	0.0115(0.0057)	0.0016(0.0017)	0.0011(0.0019)	0.2372(0.0064)		0.1631
2139	1.0829(0.3699)	0.4007(0.1218)	-0.2017(0.1282)	13.1538(0.4106)		**0.0002**
1160	0.0879(0.0262)	0.0390(0.0098)	-0.0188(0.0093)	1.0070(0.0292)		**2.14E-05**
1170	0.0772(0.0152)	0.0028(0.0040)	0.0007(0.0050)	0.5661(0.0164)		0.5017
1180	0.1361(0.0252)	0.0001(0.0058)	-0.0012(0.0079)	0.8538(0.0269)		0.9831
1190	0.0164(0.0076)	0.0052(0.0022)	-0.0057(0.0026)	0.3099(0.0086)		0.0273
1200	0.0248(0.0116)	-0.0049(0.0025)	0.0084(0.0037)	0.4974(0.0130)	Excluded	
1249	0.1317(0.0384)	-0.0043(0.0078)	0.0063(0.0118)	1.3732(0.0417)	Excluded	
20116	0.0385(0.0114)	0.0006(0.0029)	0.0072(0.0037)	0.4427(0.0125)	Excluded	
1717	0.0740(0.0095)	0.0018(0.0026)	0.0272(0.0031)	0.3165(0.0098)	Excluded	
1687	0.0206(0.0029)	-0.0003(0.0007)	0.0022(0.0009)	0.0970(0.0030)	Excluded	
1697	0.0292(0.0029)	0.0010(0.0007)	-0.0017(0.0009)	0.0828(0.0029)		0.1817
2714	0.5038(0.0998)	-0.0131(0.0172)	0.0203(0.0266)	2.0599(0.1023)	Excluded	
2734	0.1687(0.0522)	0.0196(0.0120)	-0.0232(0.0144)	1.2134(0.0552)		0.1677
2744	0.2291(0.0878)	0.0000(0.0153)	0.0221(0.0215)	1.4305(0.0908)	Excluded	
2375	0.1770(0.0141)	0.0431(0.0138)	0.0028(0.0029)	-0.0003(0.0031)		0.3597
2395	0.4911(0.0708)	0.0077(0.0129)	-0.0036(0.0154)	0.6604(0.0689)		0.7761
1940	0.0108(0.0050)	-0.0004(0.0012)	0.0018(0.0016)	0.2029(0.0056)	Excluded	
1960	0.0139(0.0053)	-0.0004(0.0012)	0.0015(0.0017)	0.2157(0.0059)	Excluded	
2000	0.0117(0.0058)	-0.0004(0.0013)	0.0008(0.0019)	0.2336(0.0065)	Excluded	
20127	0.5977(0.2965)	0.0003(0.0669)	0.0382(0.0910)	9.7643(0.3257)	Excluded	
2070	0.0295(0.0106)	-0.0022(0.0030)	0.0201(0.0036)	0.4233(0.0118)	Excluded	
2080	0.0356(0.0169)	0.0114(0.0053)	-0.0031(0.0059)	0.6828(0.0191)		0.0148
404	12441.93(4683.00)	-1523.68(964.68)	7665.23(1146.36)	75961.29(4814.57)	Excluded	
2217	47.6927(7.7861)	-1.8024(1.2492)	-2.3755(2.1523)	222.6492(8.0704)	Excluded	
2247	0.0099(0.0037)	0.0000(0.0009)	-0.0034(0.0012)	0.1617(0.0042)	Excluded	
2443	0.0034(0.0010)	0.0030(0.0005)	0.0003(0.0004)	0.0357(0.0011)		**4.88E-29**
6138	0.1327(0.0205)	0.0398(0.0081)	-0.0001(0.0072)	0.6825(0.0219)		**1.81E-13**
699	5.9094(1.9164)	0.4690(0.4016)	-2.1491(0.6147)	84.6077(2.1306)	Excluded	
738	0.0738(0.0357)	-0.0053(0.0086)	0.0348(0.0112)	1.2983(0.0393)	Excluded	

Table S15. **Genetic variance, interaction variance and their covariance component estimates for 58 UKBB phenotypes with significant heritabilities across POP2+POP3 with the covariate PC2.** The estimates which were not within the valid parameter space are excluded for follow-up analyses as shown in the column "Note" (in bold we marked the significant ones by P < 0.05/116 = 0.0004 after Bonferroni correction). SE denotes standard error. DF denotes degree of freedom.

UKB data-field	$var(\mathbf{g_0})$ (SE)	$var(\mathbf{g_1})$ (SE)	$cov(\mathbf{g_0}, \mathbf{g_1})$ (SE)	$var(\mathbf{e_0})$ (SE)	Note	P-value by LRT comparing with null model (DF =2)
48	29.2708(3.4812)	0.7248(0.4715)	4.0443(1.0811)	112.0167(3.5482)		**0.0002**
49	17.6501(2.1581)	0.5207(0.3017)	2.3922(0.6738)	69.3360(2.2038)		0.0011
50	19.4296(1.0298)	-0.0555(0.1421)	-0.3130(0.3197)	20.0123(0.9239)	Excluded	
20015	4.7441(0.3326)	0.0380(0.0521)	-0.0340(0.1065)	8.2553(0.3156)		0.3739
21001	5.2462(0.5654)	0.0856(0.0771)	0.4591(0.1765)	17.5269(0.5703)		0.0203
21002	50.3179(4.9792)	1.2034(0.7069)	4.8923(1.5357)	147.2704(4.9679)		0.0047
102	14.4908(2.9472)	0.5656(0.4550)	1.7582(0.9310)	105.5717(3.1365)		0.1565
4079	15.3282(2.7745)	0.3065(0.3380)	1.5207(0.8435)	94.6158(2.9111)		0.1976
4080	40.0402(7.6878)	-0.7162(0.8887)	-2.4181(2.4035)	270.6507(8.1186)	Excluded	
78	0.3100(0.0677)	-0.0042(0.0064)	-0.0094(0.0160)	1.1911(0.0685)	Excluded	
3143	4.4414(0.7217)	-0.1123(0.0607)	-0.2243(0.1658)	11.0458(0.7144)	Excluded	
3144	70.1760(16.3260)	-0.9640(1.5654)	-3.2945(3.8860)	291.8513(16.5557)	Excluded	
3147	97.2343(21.2319)	-1.3135(1.9993)	-2.9516(5.0193)	373.7378(21.4770)	Excluded	
3148	0.0040(0.0008)	-0.0001(0.0001)	-0.0001(0.0002)	0.0148(0.0009)	Excluded	
1558	0.2557(0.0540)	0.0194(0.0095)	0.0409(0.0179)	2.0525(0.0581)		0.0473
1598	3.8801(0.7132)	5.1373(0.5082)	-5.9721(0.3492)	22.1056(0.6559)	Excluded	
1628	0.0309(0.0126)	0.0030(0.0019)	0.0083(0.0040)	0.4714(0.0138)		0.1290
1309	0.0410(0.0369)	-0.0179(0.0045)	-0.0739(0.0120)	2.2874(0.0433)	Excluded	
1319	0.2761(0.0907)	0.3954(0.0490)	-0.1803(0.0353)	3.5394(0.0993)		**1.73E-78**
1329	0.0381(0.0179)	0.0035(0.0030)	0.0118(0.0059)	0.7667(0.0200)	Excluded	
1339	0.0289(0.0140)	0.0054(0.0029)	0.0068(0.0048)	0.5931(0.0157)		0.1734
1349	0.1006(0.0269)	-0.0105(0.0030)	-0.0254(0.0088)	1.0845(0.0292)	Excluded	
1478	0.0803(0.0189)	0.0006(0.0033)	-0.0136(0.0064)	0.7334(0.0204)	Excluded	
1518	0.0318(0.0084)	-0.0006(0.0012)	-0.0037(0.0027)	0.3324(0.0091)	Excluded	
1528	0.7688(0.1583)	0.1635(0.0367)	0.2872(0.0542)	5.8442(0.1708)		**2.45E-07**
1548	0.0222(0.0085)	0.0006(0.0014)	0.0012(0.0029)	0.3538(0.0094)		0.9130

1110	0.1398(0.0327)	0.0191(0.0062)	0.0634(0.0113)	1.5020(0.0368)	Excluded	
1130	0.0361(0.0189)	0.0031(0.0035)	-0.0057(0.0060)	0.6625(0.0206)		0.0558
2237	0.0120(0.0057)	-0.0012(0.0006)	-0.0016(0.0017)	0.2397(0.0063)	Excluded	
2139	1.0287(0.3688)	0.3196(0.0861)	0.3142(0.1295)	13.2980(0.4027)		**0.0001**
1160	0.0903(0.0262)	0.0302(0.0067)	0.0249(0.0095)	1.0139(0.0286)		**2.26E-07**
1170	0.0783(0.0152)	-0.0005(0.0024)	-0.0080(0.0051)	0.5683(0.0162)	Excluded	
1180	0.1367(0.0252)	-0.0025(0.0032)	-0.0089(0.0078)	0.8557(0.0265)	Excluded	
1190	0.0164(0.0076)	0.0006(0.0012)	0.0013(0.0025)	0.3145(0.0084)		0.8662
1200	0.0250(0.0117)	0.0002(0.0017)	-0.0000(0.0039)	0.4922(0.0130)		0.9858
1249	0.1332(0.0384)	-0.0064(0.0041)	-0.0053(0.0116)	1.3741(0.0413)	Excluded	
20116	0.0395(0.0114)	0.0031(0.0020)	-0.0018(0.0038)	0.4393(0.0124)		0.0186
1717	0.0653(0.0096)	0.0024(0.0020)	-0.0065(0.0033)	0.3243(0.0100)		0.0020
1687	0.0208(0.0029)	-0.0005(0.0003)	-0.0015(0.0009)	0.0970(0.0030)	Excluded	
1697	0.0292(0.0029)	0.0000(0.0004)	0.0004(0.0009)	0.0837(0.0029)	Excluded	
2714	0.5008(0.0998)	0.0003(0.0128)	-0.0142(0.0272)	2.0492(0.1016)	Excluded	
2734	0.1625(0.0517)	0.0164(0.0074)	0.0425(0.0144)	1.2231(0.0541)		0.0153
2744	0.2304(0.0878)	0.0110(0.0120)	0.0131(0.0215)	1.4189(0.0902)		0.6456
2375	0.0434(0.0138)	0.0004(0.0015)	-0.0016(0.0031)	0.1792(0.0139)		0.5309
2395	0.4938(0.0708)	-0.0002(0.0068)	0.0026(0.0155)	0.6658(0.0683)	Excluded	
1940	0.0104(0.0050)	-0.0005(0.0006)	-0.0007(0.0016)	0.2034(0.0055)	Excluded	
1960	0.0134(0.0053)	0.0004(0.0008)	0.0019(0.0018)	0.2153(0.0058)		0.5748
2000	0.0118(0.0057)	0.0005(0.0009)	0.0014(0.0019)	0.2327(0.0064)		0.7379
20127	0.5881(0.2963)	0.0198(0.0407)	0.0543(0.0914)	9.7560(0.3221)		0.8401
2070	0.0280(0.0106)	-0.0027(0.0016)	-0.0108(0.0033)	0.4255(0.0117)	Excluded	
2080	0.0357(0.0169)	0.0062(0.0031)	0.0078(0.0057)	0.6881(0.0187)		0.0739
404	10582.50(4798.56)	2663.16(877.46)	1545.96(1166.36)	73836.10(4949.27)		0.0004
2217	47.5311(7.7886)	-0.3026(0.8634)	2.2033(2.2680)	221.2215(8.0147)	Excluded	
2247	0.0054(0.0025)	-0.0001(0.0003)	0.0022(0.0008)	0.1429(0.0031)	Excluded	
2443	0.0037(0.0010)	0.0015(0.0003)	0.0006(0.0004)	0.0368(0.0011)		**1.15E-11**
6138	0.1356(0.0206)	0.0211(0.0049)	0.0230(0.0073)	0.6986(0.0216)		**9.21E-06**
699	3.3450(1.4937)	0.3436(0.2089)	1.9081(0.5026)	83.7636(1.7889)	Excluded	
738	0.0800(0.0358)	0.0043(0.0061)	-0.0168(0.0112)	1.2832(0.0390)		0.0239

Table S16. **The G×P interaction analysis results for 13 phenotypes across POP2+POP3.** The phenotypic values were adjusted by basic plus additional confounders of fixed effects and transformed by rank-based INT (in bold we marked the significant ones by P < 0.05/116 = 0.0004). The estimates which were not within the valid parameter space are excluded. SE denotes standard error. DF denotes degree of freedom.

UKBB data-field	Phenotypes	*P*-value by LRT (G×P RNM with PC1 vs. baseline model) (DF =2)	*P*-value by LRT (G×P RNM with PC2 vs. baseline model) (DF =2)
48	Waist circumference	**2.92E-06**	0.0004
49	Hip circumference	0.0070	0.0011
21002	Weight	**0.0002**	0.0252
78	Heel bone mineral density (BMD) T-score, automated	0.0852	0.8836
3144	Heel Broadband ultrasound attenuation, direct entry	0.0441	0.8292
3147	Heel quantitative ultrasound index (QUI), direct entry	0.0853	0.8836
3148	Heel bone mineral density (BMD)	0.0782	0.8718
1528	Water intake	0.7586	0.8449
1319	Dried fruit intake	Excluded	Excluded
1160	Sleep duration	0.0703	0.0840
2443	Diabetes diagnosed by doctor	**6.65E-11**	**3.73E-08**
2139	Age first had sexual intercourse	**7.86E-05**	0.0071
6138	Qualifications	**1.06E-15**	0.0162

Notes: the basic confounders are sex, year of birth, age at recruitment, genotype measurement batch, UKBB assessment centre and the first 20 ancestry PCs provided by UKBB.

For phenotypes data-fields 48, 49 and 21002, additional confounders are: “Townsend deprivation index at recruitment (data-field 189)”, “major dietary changes in the last 5 years (data-field 1538)”, “variation in diet (data-field 1548)”, “smoking status (data-field 20116)” and “alcohol drinker status (data-field 20117)”.

For phenotypes data-fields 78, 3144, 3147, 3148, additional confounders are: “Townsend deprivation index at recruitment (data-field 189)”, “ankle spacing width (data-field 3143)”, “heel ultrasound method (data-field 19)” and “foot measured for bone density (data-field 3081)”.

For phenotypes data-fields 1528 and 1319, additional confounders are: “Townsend deprivation index at recruitment (data-field: 189)”, “major dietary changes in the last 5 years (data-field: 1538)”, “variation in diet (data-field: 1548)” and “Vitamin and mineral supplements (data-field: 6155)”.

For phenotypes data-field 1160, additional confounders are: “residential noise pollution (data-field: 24020-24024)”, “type of accommodation lived in (data-field 670)”, “number in household (data-field 709)”, “current employment status (data-field 6142)”, “morning/evening person (chronotype) (data-field 1180)”, “sleeplessness / insomnia (data-field 1200)” and “daytime dozing/sleeping (narcolepsy) (data-field 1220)”.

For phenotypes data-fields 2443, additional confounders are: “Townsend deprivation index at recruitment (data-field 189)”, “major dietary changes in the last 5 years (data-field 1538)”, “variation in diet (data-field 1548)”, “smoking status (data-field 20116)” and “alcohol drinker status (data-field 20117)”.

For phenotype data-field 2139, additional confounders are: “Townsend deprivation index at recruitment (data-field 189)” and “qualifications (data-field 6138)”.

For phenotype data-field 6138, additional confounders are: “Townsend deprivation index at recruitment (data-field: 189)”, “current employment status (data-field: 6142)” and “average total household income before tax (data-field: 738)”.

Table S17. **Results of model comparisons (baseline, G×P/R×P and full) through LRT for two phenotypes across POP2+POP3 with the covariates PC1 and PC2**. The phenotypes were adjusted by basic plus additional confounders of fixed effects and transformed using rank-based INT. The estimates of full model for age first had sexual intercourse were not within the valid parameter space and thus were excluded. DF denotes degree of freedom.

UKBB data field	Phenotype	Covariate	Model comparison	DF	P-value by LRT
2139	Age first had sexual intercourse	PC1	Full versus R×P	2	Excluded
			Full versus G×P	2	Excluded
			Full versus GREML	4	Excluded
			R×P versus GREML	2	0.0002
			G×P versus GREML	2	7.86E-05
		PC2	Full versus R×P	2	0.4374
			Full versus G×P	2	0.8363
			Full versus GREML	4	0.0363
			R×P versus GREML	2	0.0135
			G×P versus GREML	2	0.0071
6138	Qualifications	PC1	Full versus R×P	2	0.0031
			Full versus G×P	2	0.2166
			Full versus GREML	4	8.53E-15
			R×P versus GREML	2	7.45E-14
			G×P versus GREML	2	1.06E-15
		PC2	Full versus R×P	2	0.0053
			Full versus G×P	2	0.0090
			Full versus GREML	4	0.0014
			R×P versus GREML	2	0.0273
			G×P versus GREML	2	0.0162

Table S18. **Genetic variance, interaction variance and their covariance component estimates for 70 UKBB phenotypes with significant heritability across POP1+POP3 with the covariate PC1.** The estimates which were not within the valid parameter space were excluded for follow-up analyses as shown in the column "Note" (in bold we marked the significant ones by P < 0.05/140 = 3.57E-4 after Bonferroni correction). SE denotes standard error. DF denotes degree of freedom.

UKBB data-field	$var(\mathbf{g_0})$ (SE)	$var(\mathbf{g_1})$ (SE)	$cov(\mathbf{g_0}, \mathbf{g_1})$ (SE)	$var(\mathbf{e_0})$ (SE)	Note	*P*-value by LRT comparing with null model (DF =2)
48	26.9732(3.4423)	-3.0381(1.0703)	-0.3651(0.8118)	123.2641(3.7130)	Excluded	
49	18.9254(2.0988)	-1.5711(0.6476)	0.1017(0.4952)	71.9214(2.2336)	Excluded	
50	21.3636(0.9865)	-0.0227(0.2920)	-0.2788(0.2303)	17.6890(0.9035)	Excluded	
20015	5.0084(0.3254)	-0.0881(0.0982)	-0.2779(0.0776)	8.3251(0.3215)	Excluded	
21001	5.0703(0.5535)	-0.5280(0.1655)	-0.1188(0.1288)	18.9252(0.5864)	Excluded	
21002	49.9582(4.9475)	-4.6811(1.4725)	-1.4562(1.1500)	162.5982(5.1971)	Excluded	
102	19.3238(3.1377)	-1.1396(1.0436)	-0.4463(0.7590)	112.5584(3.4496)	Excluded	
4079	15.4216(2.6480)	-0.0335(0.8990)	-0.7379(0.6418)	93.5503(2.9202)	Excluded	
4080	44.1031(7.8598)	1.9094(2.7565)	-1.6888(1.9399)	277.0338(8.7000)		0.6187
78	0.4320(0.0586)	0.0259(0.0167)	-0.0013(0.0116)	0.9662(0.0597)		0.2742
3143	3.4326(0.6153)	-0.4373(0.1524)	0.0428(0.1118)	12.0823(0.6441)	Excluded	
3144	105.0637(13.91)	6.8594(4.03)	0.9229(2.78)	229.8112(14.19)		0.1690
3147	135.5514(18.39)	8.1333(5.23)	-0.4124(3.63)	303.1400(18.73)		0.2740
3148	0.0055(0.0007)	0.0004(0.0002)	0.0000(0.0001)	0.0118(0.0007)		0.1049
1558	0.2076(0.0490)	-0.0113(0.0178)	0.0099(0.0125)	2.0548(0.0561)	Excluded	
1309	0.1409(0.0531)	0.2320(0.0261)	0.1177(0.0160)	2.1870(0.0613)		**3.31E-47**
1319	0.2023(0.0722)	-0.2698(0.0118)	0.1448(0.0151)	3.7380(0.0821)	Excluded	
1329	0.0483(0.0172)	-0.0014(0.0064)	-0.0109(0.0046)	0.7691(0.0201)	Excluded	
1339	0.0278(0.0132)	-0.0032(0.0048)	-0.0030(0.0034)	0.5913(0.0154)	Excluded	
1349	0.0620(0.0229)	0.0154(0.0091)	0.0146(0.0061)	0.9958(0.0266)		0.0037
1359	0.0438(0.0174)	-0.0038(0.0065)	0.0146(0.0045)	0.7825(0.0204)	Excluded	
1369	0.0320(0.0152)	0.0030(0.0057)	-0.0015(0.0039)	0.6643(0.0176)		0.8446
1379	0.0228(0.0106)	-0.0036(0.0039)	0.0002(0.0028)	0.4814(0.0125)	Excluded	
1408	0.0565(0.0261)	0.0174(0.0102)	0.0082(0.0068)	1.1149(0.0305)		0.0534
1458	0.5171(0.1693)	0.0346(0.0648)	0.0042(0.0446)	7.3911(0.1974)		0.8447

1478	0.0627(0.0165)	-0.0003(0.0061)	0.0015(0.0042)	0.6861(0.0189)	Excluded	
1518	0.0301(0.0073)	0.0064(0.0029)	0.0002(0.0019)	0.2976(0.0083)		0.0544
1528	0.3581(0.1246)	0.0089(0.0441)	0.0939(0.0317)	5.3408(0.1427)	Excluded	
1110	0.1215(0.0381)	-0.0065(0.0139)	-0.0040(0.0097)	1.6230(0.0438)	Excluded	
2237	0.0175(0.0056)	-0.0004(0.0021)	-0.0037(0.0015)	0.2411(0.0065)	Excluded	
2139	2.1102(0.3636)	-0.0637(0.1198)	-0.1399(0.0873)	12.4525(0.3993)	Excluded	
1160	0.0885(0.0248)	-0.0125(0.0089)	-0.0169(0.0064)	1.0712(0.0286)	Excluded	
1170	0.0707(0.0134)	0.0013(0.0048)	0.0070(0.0034)	0.5228(0.0150)		0.0893
1180	0.1505(0.0234)	-0.0002(0.0079)	0.0053(0.0056)	0.7792(0.0255)	Excluded	
1190	0.0320(0.0076)	-0.0024(0.0027)	-0.0028(0.0019)	0.3108(0.0086)	Excluded	
1200	0.0499(0.0112)	-0.0008(0.0041)	-0.0028(0.0029)	0.4608(0.0128)	Excluded	
1210	0.0165(0.0052)	-0.0018(0.0018)	-0.0008(0.0013)	0.2037(0.0059)	Excluded	
1239	0.0098(0.0038)	0.0021(0.0015)	0.0018(0.0010)	0.1643(0.0044)		0.0322
1249	0.1658(0.0433)	-0.0255(0.0141)	-0.0368(0.0105)	1.6251(0.0489)	Excluded	
20116	0.0564(0.0109)	-0.0015(0.0038)	-0.0059(0.0027)	0.4305(0.0123)	Excluded	
20160	0.0214(0.0053)	-0.0000(0.0019)	-0.0036(0.0014)	0.2098(0.0060)	Excluded	
1717	0.0606(0.0078)	0.0020(0.0027)	0.0046(0.0019)	0.2723(0.0084)		0.0252
1687	0.0148(0.0026)	-0.0003(0.0009)	-0.0003(0.0006)	0.0992(0.0029)	Excluded	
1697	0.0318(0.0027)	-0.0005(0.0009)	-0.0000(0.0007)	0.0826(0.0028)	Excluded	
20022	0.0482(0.0160)	0.0091(0.0050)	-0.0099(0.0034)	0.3664(0.0172)		0.0052
2714	0.4597(0.0983)	-0.0166(0.0280)	-0.0154(0.0196)	2.1025(0.1048)	Excluded	
2734	0.1398(0.0520)	-0.0207(0.0151)	-0.0069(0.0101)	1.2594(0.0564)	Excluded	
2375	0.0424(0.0108)	-0.0003(0.0025)	0.0012(0.0018)	0.1605(0.0112)	Excluded	
2395	0.5203(0.0653)	-0.0161(0.0138)	-0.0100(0.0108)	0.6877(0.0641)	Excluded	
1920	0.0277(0.0054)	-0.0008(0.0019)	-0.0006(0.0014)	0.2103(0.0060)	Excluded	
1930	0.0141(0.0051)	-0.0002(0.0019)	-0.0003(0.0013)	0.2214(0.0059)	Excluded	
1940	0.0199(0.0046)	0.0015(0.0017)	0.0007(0.0012)	0.1801(0.0052)		0.4857
1950	0.0131(0.0052)	0.0008(0.0020)	0.0012(0.0014)	0.2258(0.0061)		0.5554
1960	0.0228(0.0051)	-0.0010(0.0019)	0.0001(0.0013)	0.2091(0.0058)	Excluded	
1970	0.0101(0.0037)	-0.0002(0.0014)	0.0028(0.0010)	0.1627(0.0044)	Excluded	
1980	0.0227(0.0053)	0.0004(0.0020)	-0.0001(0.0014)	0.2165(0.0061)		0.9829
1990	0.0170(0.0033)	0.0030(0.0013)	0.0027(0.0009)	0.1275(0.0037)		**4.17E-05**
2000	0.0187(0.0055)	0.0000(0.0020)	-0.0001(0.0014)	0.2248(0.0063)	Excluded	
20127	1.1105(0.2743)	0.0380(0.0951)	0.0126(0.0658)	9.1067(0.3058)		0.8802

2030	0.0140(0.0045)	0.0013(0.0017)	0.0002(0.0012)	0.1869(0.0051)		0.6694
2040	0.0146(0.0046)	0.0039(0.0018)	0.0032(0.0012)	0.1872(0.0053)		0.0005
2080	0.0550(0.0156)	-0.0074(0.0055)	-0.0010(0.0039)	0.6471(0.0179)	Excluded	
2090	0.0101(0.0046)	-0.0015(0.0017)	0.0000(0.0012)	0.2103(0.0055)	Excluded	
20023	799.12(238.06)	-175.87(84.96)	-191.32(60.37)	10396.08(275.15)	Excluded	
2217	38.2853(7.0677)	-0.6904(2.3170)	0.4299(1.6681)	234.4942(7.7601)	Excluded	
2443	0.0022(0.0009)	-0.0016(0.0003)	-0.0007(0.0002)	0.0437(0.0011)	Excluded	
2463	0.0046(0.0020)	0.0005(0.0007)	-0.0007(0.0005)	0.0864(0.0023)		0.3925
6138	0.1751(0.0218)	-0.0111(0.0072)	-0.0335(0.0057)	0.8129(0.0239)	Excluded	
738	0.0742(0.0300)	0.0068(0.0108)	0.0106(0.0074)	1.1533(0.0343)		0.2159
135	0.2616(0.0697)	-0.0234(0.0244)	-0.0641(0.0179)	2.9658(0.0800)	Excluded	

Table S19. Genetic variance, interaction variance and their covariance component estimates for 70 UKBB phenotypes with significant heritability across POP1+POP3 with the covariate PC2. The estimates which were not within the valid parameter space were excluded for follow-up analyses as shown in the column "Note" (in bold we marked the significant ones by P < 0.05/140 = 3.57E-4 after Bonferroni correction). SE denotes standard error. DF denotes degree of freedom.

UKB data-field	$var(\mathbf{g_0})$ (SE)	$var(\mathbf{g_1})$ (SE)	$cov(\mathbf{g_0}, \mathbf{g_1})$ (SE)	$var(\mathbf{e_0})$ (SE)	Note	P-value by LRT comparing with null model (DF =2)
48	26.7422(3.4436)	-0.1893(1.1229)	-0.1299(0.8292)	120.6369(3.7013)	Excluded	
49	18.8882(2.0995)	-0.1786(0.6798)	-0.0838(0.5078)	70.5667(2.2352)	Excluded	
50	21.4270(0.9869)	0.2401(0.2877)	-0.2296(0.2334)	17.3698(0.9027)		0.3257
20015	5.0044(0.3256)	-0.0912(0.0954)	0.1491(0.0767)	8.3308(0.3217)	Excluded	
21001	5.0436(0.5541)	0.0966(0.1820)	0.0217(0.1359)	18.3263(0.5879)		0.8630
21002	49.7739(4.9513)	0.8844(1.6148)	0.7226(1.2010)	157.2047(5.1951)		0.7480
102	19.2398(3.1360)	-0.6202(1.0479)	0.6924(0.7641)	112.1195(3.4616)	Excluded	
4079	15.4377(2.6475)	0.7679(0.8959)	0.1859(0.6480)	92.7287(2.9115)		0.6815
4080	43.9619(7.8596)	1.0961(2.6020)	0.4277(1.8981)	277.9730(8.6237)		0.9043
78	0.4317(0.0587)	0.0325(0.0164)	-0.0007(0.0115)	0.9599(0.0595)		0.1129
3143	3.4977(0.6161)	0.0292(0.1624)	0.0787(0.1188)	11.5526(0.6400)		0.7992
3144	104.8254(13.91)	7.4758(3.9089)	-1.1092(2.7636)	229.4276(14.09)		0.1078
3147	135.4496(18.40)	10.2016(5.1359)	-0.2191(3.6159)	301.1764(18.65)		0.1129
3148	0.0055(0.0007)	0.0005(0.0002)	-0.0000(0.0001)	0.0117(0.0007)	Excluded	
1558	0.2083(0.0490)	0.0071(0.0174)	0.0105(0.0127)	2.0358(0.0557)		0.6863
1309	0.1198(0.0539)	0.1287(0.0229)	-0.0185(0.0156)	2.3122(0.0626)		**3.55E-12**
1319	0.3045(0.0757)	-0.1335(0.0212)	-0.2401(0.0191)	3.5024(0.0849)	Excluded	
1329	0.0479(0.0172)	0.0015(0.0064)	0.0093(0.0046)	0.7665(0.0201)	Excluded	
1339	0.0275(0.0132)	-0.0010(0.0048)	0.0057(0.0034)	0.5893(0.0154)	Excluded	
1349	0.0655(0.0228)	0.0201(0.0089)	-0.0217(0.0061)	0.9877(0.0264)		**8.22E-06**
1359	0.0450(0.0175)	0.0036(0.0065)	-0.0034(0.0046)	0.7740(0.0203)		0.5708
1369	0.0330(0.0152)	-0.0036(0.0054)	-0.0053(0.0038)	0.6699(0.0176)	Excluded	
1379	0.0239(0.0106)	0.0031(0.0040)	-0.0049(0.0028)	0.4737(0.0124)		0.1112
1408	0.0589(0.0261)	0.0135(0.0098)	-0.0192(0.0068)	1.1164(0.0303)		0.0021
1458	0.5162(0.1692)	-0.0161(0.0610)	0.0156(0.0441)	7.4429(0.1962)	Excluded	

1478	0.0639(0.0164)	0.0147(0.0062)	-0.0104(0.0043)	0.6699(0.0187)		0.0016
1518	0.0302(0.0073)	-0.0008(0.0027)	-0.0007(0.0019)	0.3048(0.0084)	Excluded	
1528	0.3490(0.1250)	0.0833(0.0472)	0.0414(0.0326)	5.2760(0.1440)		0.1311
1110	0.1214(0.0381)	0.0006(0.0136)	0.0055(0.0097)	1.6160(0.0439)		0.8467
2237	0.0178(0.0056)	0.0011(0.0020)	0.0017(0.0014)	0.2393(0.0065)		0.4698
2139	2.1346(0.3641)	0.1096(0.1260)	-0.1346(0.0874)	12.2547(0.3983)		0.1593
1160	0.0873(0.0248)	-0.0057(0.0087)	0.0128(0.0064)	1.0656(0.0286)	Excluded	
1170	0.0702(0.0134)	-0.0016(0.0046)	-0.0068(0.0033)	0.5262(0.0150)	Excluded	
1180	0.1505(0.0234)	-0.0005(0.0076)	-0.0079(0.0055)	0.7795(0.0254)	Excluded	
1190	0.0317(0.0076)	-0.0017(0.0026)	-0.0001(0.0019)	0.3104(0.0086)	Excluded	
1200	0.0503(0.0112)	-0.0053(0.0038)	0.0002(0.0028)	0.4649(0.0127)	Excluded	
1210	0.0166(0.0052)	-0.0004(0.0018)	0.0015(0.0013)	0.2021(0.0059)	Excluded	
1239	0.0103(0.0038)	0.0016(0.0014)	-0.0041(0.0010)	0.1644(0.0044)	Excluded	
1249	0.1658(0.0433)	-0.0182(0.0140)	0.0322(0.0105)	1.6177(0.0487)	Excluded	
20116	0.0565(0.0109)	0.0042(0.0039)	0.0006(0.0028)	0.4246(0.0122)		0.5405
20160	0.0214(0.0053)	-0.0009(0.0018)	0.0021(0.0013)	0.2107(0.0060)	Excluded	
1717	0.0608(0.0078)	0.0027(0.0026)	0.0055(0.0020)	0.2714(0.0084)		0.0181
1687	0.0148(0.0026)	-0.0005(0.0009)	0.0000(0.0006)	0.0993(0.0029)	Excluded	
1697	0.0319(0.0027)	0.0012(0.0009)	-0.0001(0.0007)	0.0809(0.0028)		0.3524
20022	0.0494(0.0160)	0.0051(0.0045)	0.0035(0.0033)	0.3692(0.0172)		0.3576
2714	0.4658(0.0983)	-0.0174(0.0268)	0.0073(0.0194)	2.0974(0.1042)	Excluded	
2734	0.1358(0.0520)	-0.0088(0.0143)	0.0016(0.0102)	1.2515(0.0562)	Excluded	
2375	0.0427(0.0107)	0.0033(0.0026)	-0.0027(0.0019)	0.1566(0.0111)		0.0858
2395	0.5182(0.0654)	-0.0055(0.0146)	0.0025(0.0109)	0.6791(0.0649)	Excluded	
1920	0.0278(0.0054)	0.0013(0.0019)	0.0013(0.0014)	0.2082(0.0060)		0.5842
1930	0.0141(0.0051)	0.0012(0.0019)	0.0009(0.0013)	0.2199(0.0059)		0.7024
1940	0.0198(0.0046)	0.0023(0.0017)	-0.0001(0.0012)	0.1794(0.0052)		0.3347
1950	0.0129(0.0052)	0.0008(0.0019)	-0.0012(0.0013)	0.2260(0.0060)		0.5622
1960	0.0229(0.0051)	0.0008(0.0018)	0.0010(0.0013)	0.2072(0.0058)		0.6997
1970	0.0097(0.0037)	0.0005(0.0014)	-0.0008(0.0010)	0.1623(0.0044)		0.5919
1980	0.0226(0.0053)	0.0000(0.0019)	-0.0004(0.0014)	0.2168(0.0061)	Excluded	
1990	0.0167(0.0033)	0.0014(0.0012)	-0.0027(0.0009)	0.1294(0.0037)		0.0011
2000	0.0186(0.0055)	0.0007(0.0020)	-0.0003(0.0014)	0.2242(0.0063)		0.9084
20127	1.1075(0.2742)	0.0482(0.0926)	0.0165(0.0652)	9.0996(0.3043)		0.8651

2030	0.0140(0.0045)	0.0001(0.0016)	-0.0007(0.0011)	0.1881(0.0051)		0.8209
2040	0.0144(0.0047)	0.0032(0.0017)	-0.0019(0.0012)	0.1881(0.0053)		0.0249
2080	0.0541(0.0156)	0.0020(0.0053)	0.0092(0.0040)	0.6386(0.0178)		0.0659
2090	0.0102(0.0046)	-0.0009(0.0017)	0.0002(0.0012)	0.2096(0.0054)	Excluded	
20023	772.3533(238.59)	-280.1147(75.61)	-74.0616(57.83)	10523.65(274.88)	Excluded	
2217	38.3644(7.0684)	0.9720(2.3483)	-0.5732(1.6921)	232.7557(7.7706)		0.8279
2443	0.0024(0.0008)	-0.0016(0.0002)	0.0005(0.0002)	0.0418(0.0009)	Excluded	
2463	0.0045(0.0020)	-0.0020(0.0007)	-0.0002(0.0005)	0.0890(0.0023)	Excluded	
6138	0.1678(0.0218)	-0.0061(0.0073)	0.0226(0.0056)	0.8148(0.0239)	Excluded	
738	0.0758(0.0300)	0.0187(0.0108)	-0.0200(0.0075)	1.1398(0.0342)		0.0015
135	0.2529(0.0698)	-0.0129(0.0244)	0.0502(0.0178)	2.9637(0.0800)	Excluded	

Table S20. Genetic variance, interaction variance and their covariance component estimates for the two phenotypes with significant interaction for the covariate PC1 across POP1+POP3 as shown in Table S18. Here the estimates were based on phenotypes adjusted by basic plus additional confounders of fixed effects and transformed by rank-based INT. The significant level is by P < 0.05/140 = 3.57E-4 after Bonferroni correction (as shown in Table S18). SE denotes standard error. DF denotes degree of freedom.

UKBB data-field	Phenotype	$var(\mathbf{g_0})$ (SE)	$var(\mathbf{g_1})$ (SE)	$cov(\mathbf{g_0}, \mathbf{g_1})$ (SE)	$var(\mathbf{e_0})$ (SE)	P-value by LRT (G×P RNM with PC1 vs. null model) (DF=2) for phenotypes adjusted by basic confounders	P-value by LRT (G×P RNM with PC1 vs. null model) (DF=2) for phenotypes adjusted by basic and additional confounders	P-value by LRT (G×P RNM with PC1 vs. null model) (DF=2) for phenotypes adjusted by basic and additional confounders as well as rank-based INT
1309	Fresh fruit intake	0.0326(0.0590)	-0.0111(0.0147)	0.0158(0.0095)	0.9784(0.0634)	**3.31E-47**	**3.33E-10**	0.2505
1990	Tense / highly strung	0.1383(0.0532)	0.0099(0.0141)	0.0034(0.0096)	0.8514(0.0561)	**4.17E-05**	0.0266	0.6467

Notes: the basic confounders are sex, year of birth, age at recruitment, genotype measurement batch, UKBB assessment centre and the first 20 ancestry PCs provided by UKBB.

For phenotypes data-field 1309, additional confounders are: “Townsend deprivation index at recruitment (data-field 189)”, “major dietary changes in the last 5 years (data-field 1538)”, “variation in diet (data-field 1548)” and “Vitamin and mineral supplements (data-field 6155)”.

For phenotypes data-field 1990, additional confounders are: “Townsend deprivation index at recruitment (data-field 189)”, “seen doctor (GP) for nerves, anxiety, tension or depression (data-field 2090)”, “seen a psychiatrist for nerves, anxiety, tension or depression (data-field 2100)”, “Illness, injury, bereavement, stress in last 2 years (data-field 6145)”, “current employment status (data-field: 6142)” and “average total household income before tax (data-field: 738)”.

Table S21. Genetic variance, interaction variance and their covariance component estimates for the two phenotypes with significant interaction for the covariate PC2 across POP1+POP3 as shown in Table S19. Here the estimates were based on phenotypes by basic plus additional confounders and rank-based INT. The significant level is by P < 0.05/140 = 3.57E-4 after Bonferroni correction (as shown in Table S18). SE denotes standard error. DF denotes degree of freedom.

UKB data-field	Phenotype	$var(\mathbf{g_0})$ (SE)	$var(\mathbf{g_1})$ (SE)	$cov(\mathbf{g_0},\mathbf{g_1})$ (SE)	$var(\mathbf{e_0})$ (SE)	P-value by LRT (G×P RNM with PC1 vs. null model) (DF=2) for phenotypes adjusted by basic confounders	P-value by LRT (G×P RNM with PC1 vs. null model) (DF=2) for phenotypes adjusted by basic and additional confounders	P-value by LRT (G×P RNM with PC1 vs. null model) (DF=2) for phenotypes adjusted by basic and additional confounders as well as rank-based INT
1309	Fresh fruit intake	0.0239(0.0592)	0.0251(0.0152)	-0.0118(0.0101)	0.9507(0.0632)	**3.55E-12**	**1.86E-14**	0.0467
1349	Processed meat intake	0.0459(0.0567)	0.0209(0.0150)	-0.0233(0.0100)	0.9330(0.0608)	**8.22E-06**	0.0016	0.0065

Notes: the basic confounders are sex, year of birth, age at recruitment, genotype measurement batch, UKBB assessment centre and the first 20 ancestry PCs provided by UKBB.

For phenotypes data-fields 1309 and 1349, additional confounders are: “Townsend deprivation index at recruitment (data-field 189)”, “major dietary changes in the last 5 years (data-field 1538)”, “variation in diet (data-field 1548)” and “Vitamin and mineral supplements (data-field 6155)”.

Table S22. **Phenotypic measure of the UKBB variable qualifications by Okbay et al.'s education year mapping.** Subjects who selected several response categories were assigned the higher qualification (data fields: 6138.0.0 to 6138.0.5).

UKBB coding	UKBB category	Education years
1	College or University degree	20
2	A levels/AS levels or equivalent	13
3	O levels/GCSEs or equivalent	10
4	CSEs or equivalent	10
5	NVQ or HND or HNC or equivalent	19
6	Other professional qualifications eg: nursing, teaching	15
-7	None of the above	7
-3	Prefer not to answer	Excluded

Table S23. Genetic variance, interaction variance and their covariance component estimates for qualifications (mapping by Okbay et al.'s education years) across three data designs with the covariates PC1 and PC2. The phenotypic values were adjusted by basic plus additional confounders of fixed effects and transformed by rank-based INT. SE denotes standard error. DF denotes degree of freedom.

Phenotype (continuous)	Data design	Covariate	$var(\mathbf{g_0})$ (SE)	$var(\mathbf{g_1})$ (SE)	$cov(\mathbf{g_0}, \mathbf{g_1})$ (SE)	$var(\boldsymbol{e_0})$ (SE)	P-value by LRT comparing with baseline model (DF = 2)
Qualifications	POP1+POP2	PC1	0.1149(0.0195)	0.0833(0.0107)	-0.0881(0.0090)	0.8040(0.0230)	5.19E-23
		PC2	0.1746(0.0201)	0.0394(0.0062)	0.0855(0.0083)	0.7880(0.0224)	1.38E-25
	POP2+POP3	PC1	0.0740(0.0242)	0.0397(0.0100)	0.0285(0.0090)	0.8813(0.0268)	8.76E-29
		PC2	0.0984(0.0249)	0.0186(0.0062)	0.0084(0.0084)	0.8822(0.0271)	0.0007
	POP1+POP3	PC1	0.1060(0.0229)	-0.0320(0.0073)	-0.0311(0.0059)	0.9257(0.0257)	Excluded
		PC2	0.0959(0.0228)	-0.0032(0.0079)	0.0313(0.0061)	0.9075(0.0254)	Excluded

Table S24. **Genetic variance, interaction variance and their covariance component estimates for qualifications (binary mapping by Gazal et al.) across three data designs with the covariates PC1 and PC2**. The phenotypic values were adjusted by basic plus additional confounders of fixed effects and transformed by rank-based INT. SE denotes standard error. DF denotes degree of freedom.

Phenotype (binary)	Data	Covariate	$var(\mathbf{g_0})$ (SE)	$var(\mathbf{g_1})$ (SE)	$cov(\mathbf{g_0}, \mathbf{g_1})$ (SE)	$var(\mathbf{e_0})$ (SE)	P-value by LRT comparing with baseline model (DF = 2)
Qualifications “college or university degree” (1) versus other six categories (0)	POP1+POP2	PC1	0.1467(0.0242)	-0.0026(0.0068)	0.0242(0.0084)	0.8500(0.0260)	Excluded
		PC2	0.1462(0.0242)	0.0068(0.0053)	-0.0276(0.0085)	0.8411(0.0258)	2.71E-07
	POP2+POP3	PC1	0.1045(0.0258)	0.0170(0.0078)	0.0048(0.0088)	0.8758(0.0280)	1.66E-05
		PC2	0.1184(0.0257)	0.0108(0.0056)	-0.0041(0.0084)	0.8696(0.0276)	0.0043
	POP1+POP3	PC1	0.0928(0.0240)	0.0207(0.0092)	0.0272(0.0062)	0.8855(0.0270)	8.82E-08
		PC2	0.0957(0.0242)	0.0108(0.0088)	-0.0171(0.0060)	0.8929(0.0271)	0.0031
Qualifications “none of the above” (1) versus other six categories (0)	POP1+POP2	PC1	0.2032(0.0216)	0.0637(0.0098)	-0.0808(0.0089)	0.7227(0.0236)	7.77E-22
		PC2	0.2509(0.0221)	0.0379(0.0063)	0.0837(0.0086)	0.7032(0.0232)	1.91E-22
	POP2+POP3	PC1	0.1552(0.0234)	0.0350(0.0100)	0.0277(0.0089)	0.7882(0.0256)	5.67E-26
		PC2	0.2114(0.0235)	0.0104(0.0053)	0.0019(0.0078)	0.7623(0.0246)	0.0449
	POP1+POP3	PC1	0.2336(0.0233)	-0.0062(0.0078)	-0.0444(0.0063)	0.7709(0.0245)	Excluded
		PC2	0.2123(0.0228)	-0.0085(0.0078)	0.0303(0.0060)	0.7934(0.0243)	Excluded

Table S25. **The distributions of original phenotypic values across POP1, POP2 and POP3 for the trait "age first had sexual intercourse".**

	Min.	1st Quartile	Median	Mean	3rd Quartile	Max.	Variance	*N*
POP1	5.00	17.00	18.00	19.03	21.00	66.00	15.6130	6647
POP2	3.00	17.00	19.00	19.27	21.00	62.00	15.5495	5924
POP3	3.00	17.00	18.00	19.11	21.00	63.00	14.6723	7102

Table S26. **The distributions of original phenotypic values across POP1, POP2 and POP3 for the trait "qualifications".** Level 1: none; Level 2: O-levels or CSEs; Level 3: A-levels, NVQ, HND, HNC or other professional qualification; Level 4: college or university degree.

	Level 1	Level 2	Level 3	Level 4	Mean	Variance	*N*
POP1	1303	2148	1769	2216	2.6587	1.1715	7436
POP2	636	807	1394	3927	3.2732	1.0015	6764
POP3	536	992	1719	4518	3.3160	0.8859	7765

Table S27. The results of linear regressions including 1 (representing POP1) and 2 (representing POP2) as independent variable across POP1+POP2. For real data of the trait qualifications, we used educational levels as dependent variable, which was adjusted by basic and additional confounders and rank-based INT (but not adjusted by PCs). For 100 replicates simulated with different selection odds ratio combinations, we used the simulated phenotypes as dependant variable, which was adjusted by rank-based INT.

	R-squared		Adjusted R-squared		-log10(P-value)	
Real data of qualifications	0.0045		0.0044		12.9914	
Selection scenarios for 100 simulation replicates	Mean	SE	Mean	SE	Mean	SE
$OR_{POP1,Y}= 2$, $OR_{POP2,Y}= 3$	0.0059	0.0004	0.0058	0.0004	10.1497	0.6567
$OR_{POP1,Y}= 2$, $OR_{POP1,Z}= 2$, $OR_{POP2,Y}= 3$, $OR_{POP2,Z}= 2$	0.0056	0.0004	0.0055	0.0004	9.6210	0.6509
$OR_{POP1,Y}= 2$, $OR_{POP1,Z}= 2$, $OR_{POP2,Y}= 3$, $OR_{POP2,Z}= 3$	0.0035	0.0003	0.0034	0.0003	6.2513	0.5188
$OR_{POP1,Y}= 2$, $OR_{POP1,Z}= 3$, $OR_{POP2,Y}= 3$, $OR_{POP2,Z}= 3$	0.0052	0.0004	0.0051	0.0004	8.9677	0.6478

Table S28. **Genetic correlation estimates between population groups (POP1, POP2 and POP3) by bivariate GREML for qualifications.** Here the qualifications were reclassified into binary measures, adjusted by basic plus additional confounders of fixed effects and transformed by rank-based INT. SE denotes standard error. P-value was obtained through a Wald test under a null hypothesis that genetic correlation equals to 1.

Binary phenotype	Genetic correlation between POP1 and POP2			Genetic correlation between POP2 and POP3			Genetic correlation between POP1 and POP3		
	Estimate	SE	P value	Estimate	SE	P value	Estimate	SE	P value
"college or university degree" versus other six educational categories	0.2940	0.3353	0.0352	0.7111	0.4226	0.4942	0.9551	0.6020	0.9405
"none of the above" versus other six educational categories	-0.0232	0.2539	5.58E-05	0.3134	0.1735	7.59E-05	0.4315	0.3045	0.0619

Table S29. **SNP-based heritabilities estimated by GREML and G×P RNM methods for two traits across POP1+POP2, POP2+POP3 and POP1+POP3.** Here the phenotypes were adjusted by basic plus additional confounders of fixed effects and transformed by rank-based INT. SE denotes standard error.

Phenotype	Estimate method	POP1+POP2		POP2+POP3		POP1+POP3	
		h^2_{SNP}	SE	h^2_{SNP}	SE	h^2_{SNP}	SE
Qualifications	GREML	0.0998	0.0262	0.1156	0.0265	0.1031	0.0247
	G×P RNM with PC1	0.1281	0.0250	0.0971	0.0272	0.1151	0.0237
	G×P RNM with PC2	0.1840	0.0219	0.1156	0.0269	0.1090	0.0241
Age first had sexual intercourse	GREML	0.1000	0.0266	0.0933	0.0258	0.1582	0.0250
	G×P RNM with PC1	0.1015	0.0267	0.0962	0.0264	0.1574	0.0249
	G×P RNM with PC2	0.1023	0.0268	0.0947	0.0261	0.1591	0.0252
